# Identifying pathways regulating the oncogenic p53 family member ΔNp63 provides therapeutic avenues for squamous cell carcinoma

**DOI:** 10.1186/s11658-022-00323-x

**Published:** 2022-02-23

**Authors:** Zuzana Pokorna, Jan Vyslouzil, Borivoj Vojtesek, Philip J. Coates

**Affiliations:** Research Center of Applied Molecular Oncology (RECAMO), Masaryk Memorial Cancer Institute, Zluty kopec 7, 656 53 Brno, Czech Republic

**Keywords:** ΔNp63, Oncogene addiction, Squamous cell carcinoma, DNA damage, Histone deacetylase inhibitors, Growth factor signaling

## Abstract

**Background:**

ΔNp63 overexpression is a common event in squamous cell carcinoma (SCC) that contributes to tumorigenesis, making ΔNp63 a potential target for therapy.

**Methods:**

We created inducible *TP63*-shRNA cells to study the effects of p63-depletion in SCC cell lines and non-malignant HaCaT keratinocytes. DNA damaging agents, growth factors, signaling pathway inhibitors, histone deacetylase inhibitors, and metabolism-modifying drugs were also investigated for their ability to influence ΔNp63 protein and mRNA levels.

**Results:**

HaCaT keratinocytes, FaDu and SCC-25 cells express high levels of ΔNp63. HaCaT and FaDu inducible *TP63*-shRNA cells showed reduced proliferation after p63 depletion, with greater effects on FaDu than HaCaT cells, compatible with oncogene addiction in SCC. Genotoxic insults and histone deacetylase inhibitors variably reduced ΔNp63 levels in keratinocytes and SCC cells. Growth factors that regulate proliferation/survival of squamous cells (IGF-1, EGF, amphiregulin, KGF, and HGF) and PI3K, mTOR, MAPK/ERK or EGFR inhibitors showed lesser and inconsistent effects, with dual inhibition of PI3K and mTOR or EGFR inhibition selectively reducing ΔNp63 levels in HaCaT cells. In contrast, the antihyperlipidemic drug lovastatin selectively increased ΔNp63 in HaCaT cells.

**Conclusions:**

These data confirm that ΔNp63-positive SCC cells require p63 for continued growth and provide proof of concept that p63 reduction is a therapeutic option for these tumors. Investigations of ΔNp63 regulation identified agent-specific and cell-specific pathways. In particular, dual inhibition of the PI3K and mTOR pathways reduced ΔNp63 more effectively than single pathway inhibition, and broad-spectrum histone deacetylase inhibitors showed a time-dependent biphasic response, with high level downregulation at the transcriptional level within 24 h. In addition to furthering our understanding of ΔNp63 regulation in squamous cells, these data identify novel drug combinations that may be useful for p63-based therapy of SCC.

**Supplementary Information:**

The online version contains supplementary material available at 10.1186/s11658-022-00323-x.

## Background

Squamous cell carcinoma (SCC) is one of the most prevalent forms of human cancer and is a major cause of mortality worldwide [1, 2]. The most common sites are skin, head and neck, esophagus, lung and anogenital regions, and SCC is classified and clinically managed according to anatomical location. Despite advances in mechanistic understanding of SCC, improvements in patient survival have been modest, indicating that effective targeted therapeutic approaches are still lacking [3, 4]. In particular, although SCCs are treated according to their site of origin, they share many biological and genetic characteristics, implying that targeting these common oncogenic pathways would be useful therapeutic approaches [1].

One common oncogenic event in SCC is overexpression of ΔNp63 [1, 5, 6], sometimes associated with *TP63* gene amplification [7, 8] although tumors without amplification also show overexpression [9]. ΔNp63 acts to maintain stem/progenitor cells, regulate differentiation and promote growth of normal squamous cells and SCCs (reviewed in [10–12]). In keeping with these roles, high ΔNp63 levels are associated with poor prognosis [13–15] and therapeutic resistance [14, 16–19]. Experimentally, ΔNp63 depletion enhances the effects of genotoxic agents on SCC cells in vitro, and ΔNp63 overexpression inhibits UV-radiation induced apoptosis of keratinocytes in vivo [16, 18, 20]. Thus, the ability to downregulate ΔNp63 would be therapeutically advantageous by the direct effects of p63 depletion on SCC cell proliferation, and/or by enhancing the effectiveness of conventional therapies. Achieving this aim in a clinical setting requires a fuller knowledge of the mechanisms involved in ΔNp63 regulation in tumor cells, which is known to involve multiple factors that induce positive and negative regulatory loops through cell-context dependent pathways [11, 12, 21].

To explore the potential for targeting ΔNp63 in SCC treatment and uncover its regulatory pathways in these cells, we first used genetic approaches to investigate the effects of ΔNp63 depletion, providing evidence for ΔNp63 oncogene addiction in SCC. To explore whether ΔNp63 depletion can be achieved using clinically available agents, we studied the effects of known squamous cell growth factors and inhibitors of their signaling pathways, histone deacetylase inhibitors (HDACi), genotoxic agents and metabolism-modifying drugs to examine in detail their effects on ΔNp63 levels in SCC cells and non-transformed keratinocytes. These data provide a comprehensive analysis of factors involved in ΔNp63 regulation and reveal complex responses to the same treatment in different cell lines, indicating that a personalized approach will be required for optimal ΔNp63 inhibition. Nonetheless, we identify genotoxicity, dual AKT/mTOR inhibition and HDACi as major downregulators of ΔNp63, and the cholesterol-lowering agent lovastation as a selective upregulator of ΔNp63 transcription in non-malignant squamous cells. Combinations of these agents dependent on tumor characteristics will be particularly useful for ΔNp63 inhibition therapy.

## Methods

All general chemicals and growth factors were obtained from Sigma-Aldrich (St Louis, MO, USA) unless stated otherwise. Drugs used in this study were of pharmaceutical grade or intended for cell culture experiments and were dissolved according to the manufacturer’s instructions (details in Additional file 1). Controls were performed using the highest volume of the corresponding solute.

### Cell culture

The SCC cell lines FaDu (human pharynx squamous cell carcinoma) [22] and SCC-25 (human squamous cell carcinoma of the tongue) [23] were obtained from ATCC (Manassas, VA, USA). HaCaT cells (spontaneously immortalized human keratinocytes that are non-tumorigenic and retain differentiation capacity) [24] were obtained from DKFZ (Heidelberg, Germany). FaDu and HaCaT cells were maintained in high glucose Dulbecco’s modified Eagle’s medium (DMEM) with 10% fetal bovine serum (FBS), 1% sodium pyruvate, and penicillin/streptomycin (Gibco, Thermo Fisher Scientific, MA, USA) at 37 °C in 5% CO_2_. SCC-25 cells were cultured in DMEM/Nutrient Mixture F-12 (DMEM/F-12) with 10% FBS, 0.4 μg/ml hydrocortisone (Lonza, Basel, Switzerland), 1% sodium pyruvate, and penicillin/streptomycin at 37 °C in 5% CO_2_.

### Inducible *TP63* knockdown cell lines

Tetracycline-inducible TRIPZ plasmids containing 3 individual shRNAs targeting *TP63* were obtained from Horizon Discovery (RHS4740-RG8626; Cambridge, UK). Lentiviral particles were produced in HEK293FT cells and used to transduce HaCaT, FaDu and SCC-25 cells (see Additional file 2 for details). Cells were selected with 1 µg/ml puromycin, resistant cells were expanded, and shRNAs were induced with 1 µg/ml doxycycline for 24 h for western blotting. Cell populations showing ΔNp63 downregulation were single-cell cloned (BD FACS Aria III, Berks., UK) and at least two individual clones were prepared for each cell line. Individual clones were re-tested by western blotting after doxycycline induction. Stable cell lines containing inducible *TP63*-shRNAs were routinely cultured in DMEM with 10% FBS and 1 µg/ml puromycin, and doxycycline was added at 1 µg/ml to induce *TP63*-shRNA.

To determine proliferation rates after depletion, cells were seeded onto sterile 8-well slides (Ibidi Gmbh, Grafelfing, Germany) for the time required for cell adhesion. Doxycycline or control medium was then added for 4 days. Cells were fixed with cold methanol/acetone (50/50) for 10 min, dried, and immunostained with mouse monoclonal anti-Ki67 antigen (MIB-1 M7240 Dako) diluted 1:250 (0.18 µg/ml) using Envision peroxidase-polymer labeled anti-mouse Ig (Dako) for 30 min and diaminobenzidine (DAB) as the chromogen. Cells were counterstained with hematoxylin. Ki67 was quantified using QuPath image analysis [25] with default settings for hematoxylin/DAB and a detection threshold of 0.25 for all images, with 3 to 5 images (more than 1200 cells) used for each clone.

For colony-forming ability measurements, 250 single *TP63*-shRNA cells/well were flow-sorted into six-well plates in triplicate. After adherence, cells were cultured with or without 1 µg/ml doxycycline for 4 days before further culture without doxycycline. Colonies were stained with crystal violet (0.5% w/v in 20% methanol) and colony numbers counted in each well. After photography, colonies were destained in 1% SDS and the amount of dissolved crystal violet from each well was determined by absorbance at 570 nm.

### Endogenous ΔNp63 regulation by DNA damage, growth factor signaling, histone deacetylase inhibitors and metabolism modifying drugs

Based on previous evidence for their ability to regulate p63 [11, 12, 21], parental HaCaT, FaDu and SCC-25 cells were treated with insulin, insulin-like growth factor 1 (IGF-1), amphiregulin, epidermal growth factor (EGF), hepatocyte growth factor (HGF) or keratinocyte growth factor (KGF) at varying doses and varying times up to 24 h. According to the type of experiment and length of exposure, cells were grown to 30–70% confluence before treatment. Signaling inhibition employed the pan-phoshatidyl-inositol-3-kinase (PI3K) inhibitor wortmannin and the PI3K p110δ subunit inhibitor CAL-101, the mTOR1/2 inhibitor (rapamycin), the dual inhibitor of PI3K/mTOR (BEZ235), the p38 mitogen-activated protein kinase (MAPK) inhibitor (SB202190) and the EGF receptor (EGFR) inhibitor (cetuximab). In some experiments, basal signaling was reduced by incubating cells overnight in medium with reduced serum (0, 0.5 or 1% FBS) before treatment for 24 h in the same medium. These cells were also compared with those grown in medium with 10% FBS throughout to investigate the effects of serum reduction on basal ΔNp63 levels. Ultraviolet C (UVC; 254 nm), cisplatin, doxorubicin and etoposide were used as DNA damaging agents. Trichostatin A, valproic acid, sodium butyrate and SAHA (vorinostat) were used as inhibitors of class I and II HDACs, and nicotinamide was used to inhibit sirtuins (class III HDACi). Metabolism was modified using lovastatin that inhibits 3-hydroxy-3-methyl-glutaryl-coenzyme A (HMG-CoA) reductase and the mevalonate pathway, or metformin that acts through AMP-activated protein kinase (AMPK)-dependent and AMPK-independent mitochondrial pathways to regulate cellular energy metabolism. Experiments employing metformin were performed in medium with low glucose (1 g/L) for 16 h before treatment in the same medium for 24 h.

### Western blotting

Protein lysates were separated on 10% polyacrylamide gels and transferred to nitrocellulose membranes (see Additional file 2 for details). Membranes were cut into upper and lower portions for ΔNp63 and β-actin detection, respectively. Blots were incubated overnight at 4 °C with 1 µg/ml mouse monoclonal antibody ΔNp63-1.1 that recognizes the unique ΔNp63 N-terminal peptide region and does not cross-react with TAp63, p53 or p73 isoforms [26, 27], or with 0.2 µg/ml β-actin (1:500, clone C4, sc-47778, Santa Cruz Biotechnology, Dallas, TX, USA) as loading control. Rabbit monoclonal antibodies to phospho-Akt (Ser473) (1:1000, 3787), phosphorylated extracellular signal-regulated protein kinase (phospho-Erk1/2) (Thr202/Tyr204) (1:1000, 4376), and phospho-70-kDa ribosomal protein S6 kinase (p70-S6 kinase) (Thr389) (1:1000, 9234) were used as controls of growth factor or inhibitor treatment, and rabbit monoclonal anti-phospho-histone H2AX (Ser139) (γH2AX, 1:1000, 9718; Cell Signaling Technology, Danvers, MA, USA) was used as a control for genotoxic agents. After incubation with primary antibodies, proteins were detected with peroxidase-coupled goat anti-mouse IgG or goat anti-rabbit IgG (Jackson Immunoresearch, West Grove, PA, USA) and bands were visualized using enhanced chemiluminescence (ECL, Amersham Pharmacia Biotech, Bucks, UK). Densitometry was performed using ImageJ (imageJ.nih.gov/ij/index.html), comparing peak areas of ΔNp63 bands to the corresponding β-actin bands.

### RNA isolation and reverse transcription quantitative PCR

Reverse transcription and quantitative PCR (RT-qPCR) was performed in specific situations where ΔNp63 protein levels were altered and where mechanistic data was not available previously. Total RNA was isolated using TRIzol reagent and 500 ng were reverse transcribed using High Capacity cDNA Reverse Transcription Kit (Applied Biosystems, Thermo Fisher, USA). Primers for *ΔNp63* and *ACTB* (β-actin) (Additional file 3) were obtained from Generi Biotech (Hradec Kralove, Czech Republic). PCR was performed on a Fast Real-Time PCR System with Sybrgreen (Applied Biosystems): 95 °C for 3 min, 50 cycles of 95 °C for 5 s and 60 °C for 25 s. At least three biological replicates were performed, and each cDNA sample was analyzed in technical triplicates. Mean cycle threshold (Ct) values were transformed into relative mRNA levels [28] and *ΔNp63* levels were normalized to *ACTB*.

### Statistical analysis

Data are presented as mean ± SEM. Statistical significance (p < 0.05) was determined using unpaired 2-tailed t-tests against control values.

## Results

### Preparation of inducible *TP63*-shRNA HaCaT and FaDu cells

Western blotting and RT-qPCR for p63 isoforms showed that HaCaT, FaDu and SCC-25 cells contain ΔNp63 mRNA and protein at high levels, with undetectable levels of TAp63 protein by western blotting and low or undetectable levels of *TAp63* mRNA by RT-qPCR, in keeping with previous data [29–31]. Thus, we used a mono-specific mouse monoclonal antibody that recognizes only ΔNp63, showing a predominant band at 72 kDa (representing ΔNp63α, the major isoform in normal squamous cells and SCC cells [29, 30]) with a minor band at approximately 68 kDa seen mainly in HaCaT cells that may represent ΔNp63β.

**Fig. 1:**
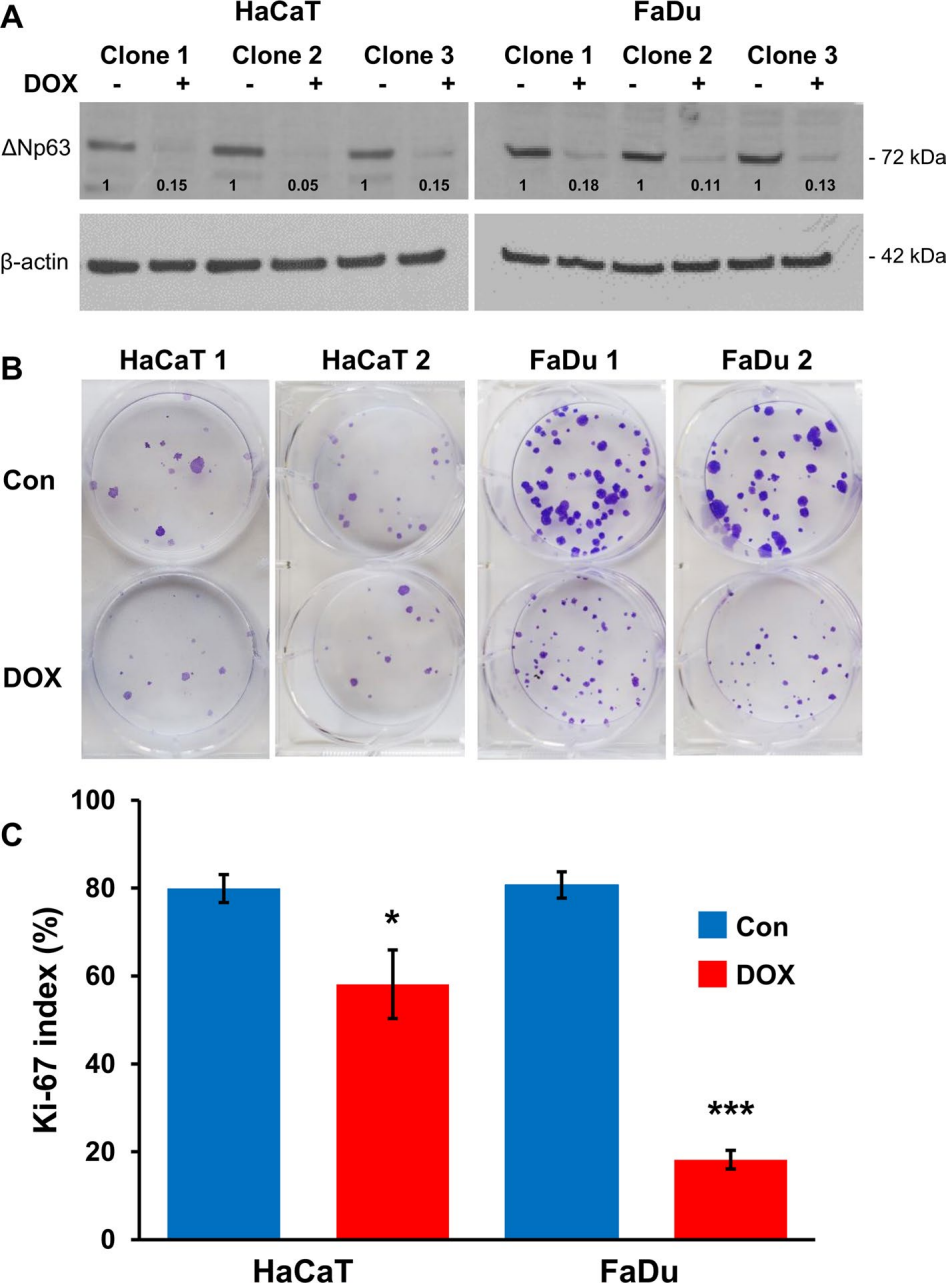
shRNA depletion of p63 reduces proliferation. **A** ΔNp63 western blotting in HaCaT or FaDu cells containing doxycycline-inducible p63 shRNAs with or without doxycycline (DOX) for 24 h. β-actin is shown as loading control. Relative densitometry measurements of ΔNp63 are shown after normalizing to β-actin; control cells are designated as 1 for each clone. **B** Crystal violet staining of colonies formed with or without doxycycline for 4 days from 2 stable clones of HaCaT or FaDu shRNA-p63 cells. **C** Percentages of Ki67 in HaCaT or FaDu cells growing with doxycycline (DOX) or control cells without doxycycline (Con) for 4 days. *p < 0.05;***p < 0.001 for doxycycline treated cells compared to the same cells growing without doxycycline

HaCaT, FaDu and SCC-25 cells were transduced with TRIPZ lentiviruses expressing TET-responsive *TP63-*shRNAs*.* Of the three shRNA sequences obtained, only #24246 (3’ untranslated region) showed effective ΔNp63 reduction after induction by doxycycline, producing 85% to 95% reduction in ΔNp63 protein levels after 24 h in three individual primary clones of HaCaT and FaDu cells (Fig. 1A). Two HaCaT and FaDu clones were subsequently single cell re-cloned and expanded for further experiments. We were unable to produce stable SCC-25 *TP63*-shRNA cells, which grew poorly after single-cell cloning and during puromycin selection and maintenance.

### ΔNp63 depletion selectively reduces colony formation and growth of SCC cells

To characterize the effects of depleting p63, stable HaCaT and FaDu *TP63-*shRNA cells were plated at cloning density and treated with doxycycline for 4 days after attachment. Clones were then allowed to grow and form colonies for 6 days (FaDu) or 10 days (HaCaT) in the absence of doxycycline. This transient p63 depletion at initial growth stages did not markedly influence the number of colonies, but significantly inhibited colony size, particularly in FaDu cells (Fig. 1B). Quantitation using crystal violet destaining (Additional file 4) confirmed that p63 depletion reduced cell numbers, with a greater effect on FaDu cells (38.2% reduction compared to 25.5% reduction in HaCaT; p = 0.0044). Similarly, Ki67 staining showed reduced percentages of proliferating cells after p63 depletion for both HaCaT and FaDu cells, with a larger effect on FaDu than HaCaT cells (77.4% reduction in FaDu compared to 27.3% reduction in HaCaT; p = 0.0056) (Fig. 1C).

### DNA damaging agents reduce ΔNp63

**Fig. 2:**
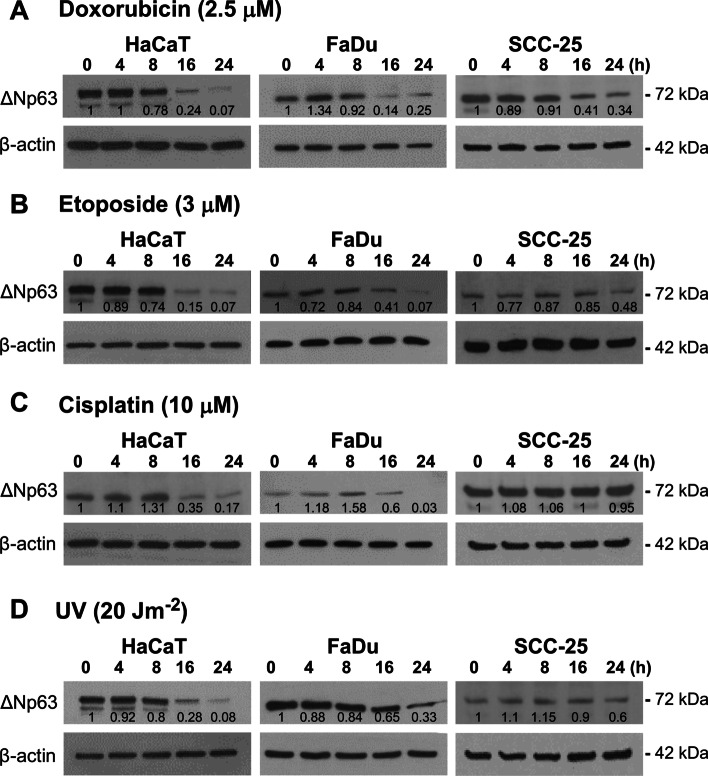
DNA damaging agents decrease ΔNp63. Representative western blots of ΔNp63 (72 kDa) in HaCaT, FaDu or SCC-25 cells taken at the indicated times after exposure to **A** doxorubicin, **B** etoposide, **C** cisplatin and **D** UVC light. β-actin (42 kDa) is shown as loading control. Relative ΔNp63 densitometry measurements normalized to β-actin are indicated

Previous studies have implicated DNA damage as a post-translational regulator of p63, where some forms of damage reduce ΔNp63 through a variety of proteasomal pathways [11, 21], although it is unclear whether different types of DNA damage have comparable effects and whether this is seen in all cells equally. To test the generability of DNA damage effects in squamous cells, HaCaT keratinocytes, FaDu and SCC cells were treated with doxorubicin, etoposide or cisplatin, or exposed to UVC radiation. Each agent was administered at varying doses and cells were collected at various times up to 24 h. The effectiveness of these treatments was demonstrated by western blotting for phospho-H2AX (Ser139) (γH2AX) as a marker of DNA damage, showing higher levels in all cells after all DNA-damaging agents (Additional file 5). Genotoxic agents caused a decrease in ΔNp63 protein 16 and 24 h after treatment, albeit with varying levels of downregulation with different agents in the different cell lines, with SCC-25 cells showing lesser effects than HaCaT or FaDu cells (Fig. 2A–D). In addition, these treatments variably increased ΔNp63 at earlier times (4 or 8 h), most notable in doxorubicin and cisplatin-treated HaCaT or FaDu cells (Fig. 2A, C). These results are in general agreement with previous data, but also indicate cell- and agent-specific responses, despite each agent causing a similar level of DNA damage in each cell line.

### Growth factor signaling regulation of ΔNp63

**Fig. 3:**
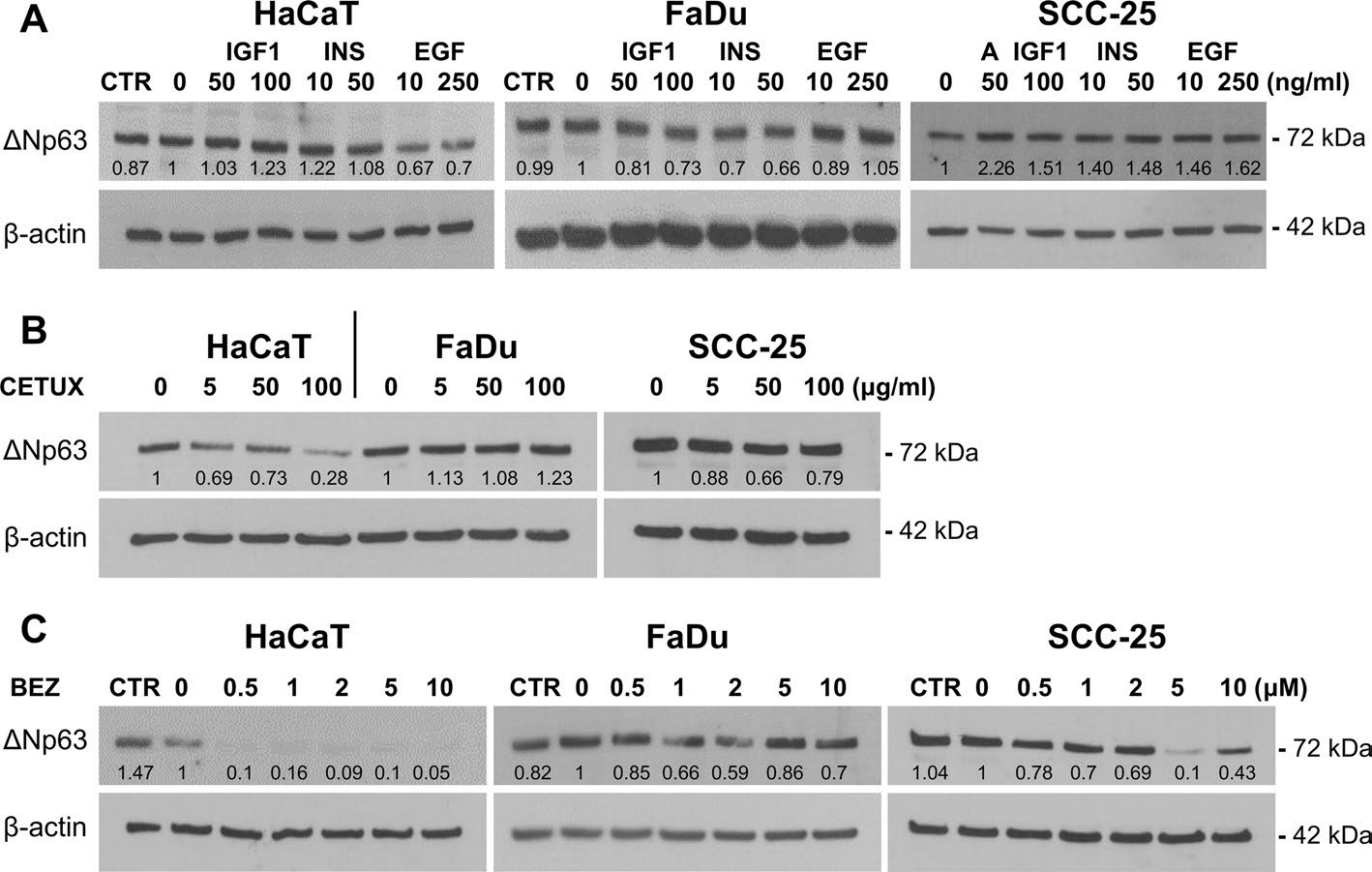
The effects of growth factor signaling pathways. Representative western blots of ΔNp63 (72 kDa) in HaCaT, FaDu or SCC-25 cells grown for 16 h in 1% FBS and collected after a further 24 h of treatment with **A** the indicated growth factors (IGF1, insulin like growth factor-1; INS, insulin; EGF, epidermal growth factor; A, amphiregulin), **B** Cetuximab (CETUX), **C** BEZ235 (BEZ). β-actin (42 kDa) is shown as the loading control. CTR represents growth in standard medium (10% FBS); 0 represents growth in 1% FBS with no addition of growth factor or inhibitor. Relative ΔNp63 densitometry measurements normalized to β-actin are indicated for the representative blots shown, with 0 used as control. See Additional file 6 for other growth factors and Additional file 7 for the effects of inhibitors on downstream signaling pathways

The effects of growth factor signaling pathways on ΔNp63 are controversial, with reports indicating both positive and negative effects for the same pathways [12]. In our experiments, addition of growth factors (insulin, IGF-1, EGF) did not alter ΔNp63 protein levels in cells under their normal growth conditions (10% FBS). Reducing the levels of endogenous growth factors by culture in medium with 1% FBS before and during growth factor treatment also did not change ΔNp63 levels compared to cells growing in 10% FBS (Fig. 3A). The addition of insulin/IGF-1 and the EGFR ligands amphiregulin and EGF to medium with 1% FBS increased the levels of ΔNp63 in SCC-25 cells but had minimal or no effects in HaCaT and FaDu cells (Fig. 3A). Cells cultured in serum-free medium before and during the 24 h treatments could not be analyzed due to extensive cell death. We also saw no effect of HGF or KGF on ΔNp63 levels under normal growth conditions or after serum depletion (see Additional file 6). Analysis of signaling activity after growth factor treatments showed minimal induction of phospho-AKT or phospho-ERK1/2 in FaDu cells, whilst phospho-ERK1/2 was increased by EGF in HaCaT cells and phospho-AKT was increased in SCC-25 cells after treatment with insulin or IGF-1 (Additional file 6).

To address the potential roles of growth factor signaling in p63 regulation further, we used small molecule or antibody inhibitors of specific pathways. Wortmannin (PI3K inhibitor) and CAL-101 (PI3K p110δ subunit inhibitor), rapamycin (mTOR1/2 inhibitor) and SB202190 (p38 MAPK inhibitor) did not cause significant changes in ΔNp63 levels, nor did they show appreciable reduction of phospho-Akt or phospho-p70 S6 kinase under the conditions used (Additional file 7). The EGFR-inhibitory antibody, cetuximab, decreased ΔNp63 levels in HaCaT cells to less than 30% of the control value, with a smaller effect in SCC-25 cells (Fig. 3B). The dual PI3K/mTOR inhibitor BEZ235 selectively downregulated ΔNp63 in HaCaT cells, with more than tenfold reduction at the lowest dose tested (0.5 µM) compared to no reduction in FaDu and SCC-25 cells at this dose, and a tenfold higher dose was required to reduce ΔNp63 in SCC-25 (Fig. 3C). All doses of BEZ235 abolished phospho-Akt in all three cell lines (Additional file 7). Taken together with the growth factor supplementation data, these results indicate that the PI3K/Akt pathway is hyperactive in FaDu cells and is therefore difficult to either inhibit or activate with growth factors or inhibitors, whereas EGFR signaling and insulin/IGF signaling contribute to ΔNp63 regulation. Importantly, combined Akt/mTOR targeting effectively reduces signaling in all cells yet reduces ΔNp63 selectively in HaCaT cells.

### Lovastatin selectively increases ΔNp63 in HaCaT cells and metformin selectively reduces ΔNp63 in FaDu cells

**Fig. 4:**
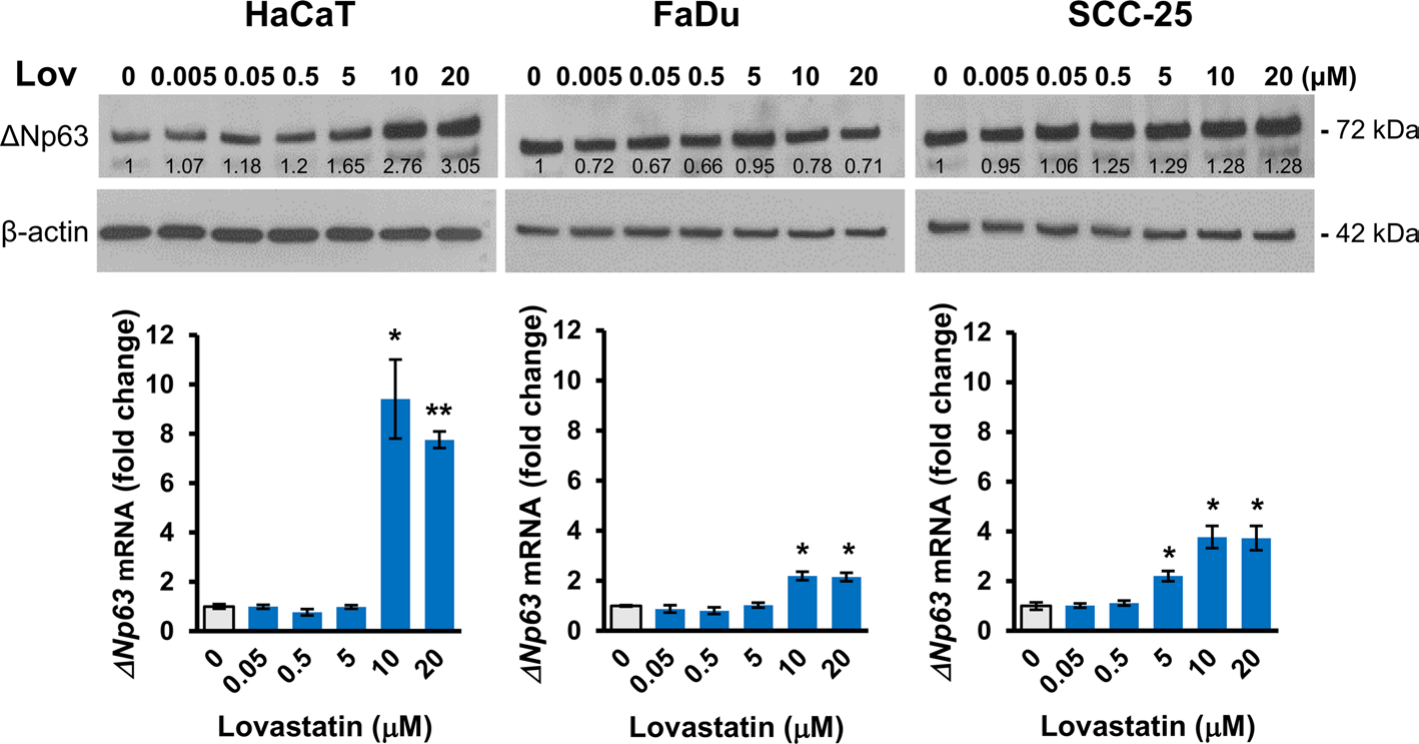
Lovastatin selectively induces ΔNp63 protein and mRNA in HaCaT cells. Top panels; Representative western blots of ΔNp63 (72 kDa) and β-actin (42 kDa) as loading control for HaCaT, FaDu and SCC-25 cells treated for 24 h with the indicated concentrations of lovastatin (Lov). Relative ΔNp63 densitometry measurements normalized to β-actin are indicated, with 0 used as control. Lower panels; RT-qPCR for *ΔNp63* mRNA shown as fold change after normalization to *ACTB* mRNA, with 0 used as control. *p < 0.05; **p < 0.01 compared to untreated cells

There is evidence that aspects of cellular metabolism are related to p63, with the glucose lowering agent metformin reportedly reducing ΔNp63 [32] or having no or minimal effects as a single agent in SCC cells [33]. Whether lipid metabolism is also a regulator is unknown. Treatment with lovastatin increased ΔNp63 protein levels in all three cell lines (Fig. 4). RT-qPCR was also performed to investigate whether this effect is at the protein or mRNA level. Induction by lovastatin was most pronounced in HaCaT cells, with a more than tenfold increase of mRNA and a correspondingly larger increase in ΔNp63 protein after 10 μM and 20 μM lovastatin for 24 h. This contrasts with the twofold and 3.5-fold increases in *ΔNp63* mRNA in FaDu and SCC-25 cells, respectively, with correspondingly smaller increases in ΔNp63 protein levels (Fig. 4). We also investigated whether metformin influenced ΔNp63 protein levels, showing a reduction of approximately 50% in FaDu cells compared to 15–20% reduction in SCC-25 and HaCaT cells, respectively, after treatment with high doses of metformin for 24 h under low glucose conditions (Additional file 8).

### HDAC inhibitors decrease ΔNp63 levels

**Fig. 5:**
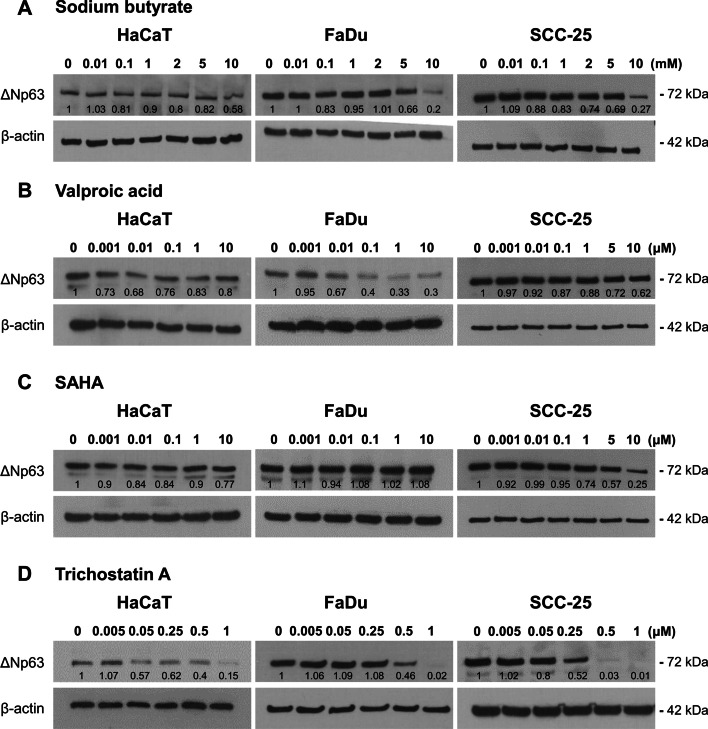
The effects of HDACi. Representative western blots of ΔNp63 (72 kDa) and β-actin (42 kDa) as loading control in HaCaT, FaDu or SCC-25 cells 24 h after treatment with **A** sodium butyrate, **B** valproic acid, **C** SAHA (vorinostat) or **D** trichostatin A. Relative ΔNp63 densitometry measurements normalized to β-actin are indicated, with 0 used as control. See Additional file 9 for nicotinamide treated cells

Previous studies have investigated acetylation as a regulator of p63 activity using HDACi treatment, with both positive and negative effects reported under different conditions [34, 35]. Whether these different effects are due to cell-type specific factors or differences in experimental conditions between studies is unclear. Moreover, whether HDACi regulation of ΔNp63 occurs at the transcriptional or protein level, and the effects of class-specific HDAci are unknown. Therefore, HaCaT, FaDu and SCC-25 cells were analyzed in dose response experiments after treatment with various HDACi for 24 h. All four broad spectrum HDACi (sodium butyrate, valproic acid, SAHA and trichostatin A) reduced ΔNp63 in these cells to varying extents (Fig. 5). Nicotinamide, an inhibitor of class III HDACs (sirtuins), reduced ΔNp63 only in FaDu cells (Additional file 9).

**Fig. 6:**
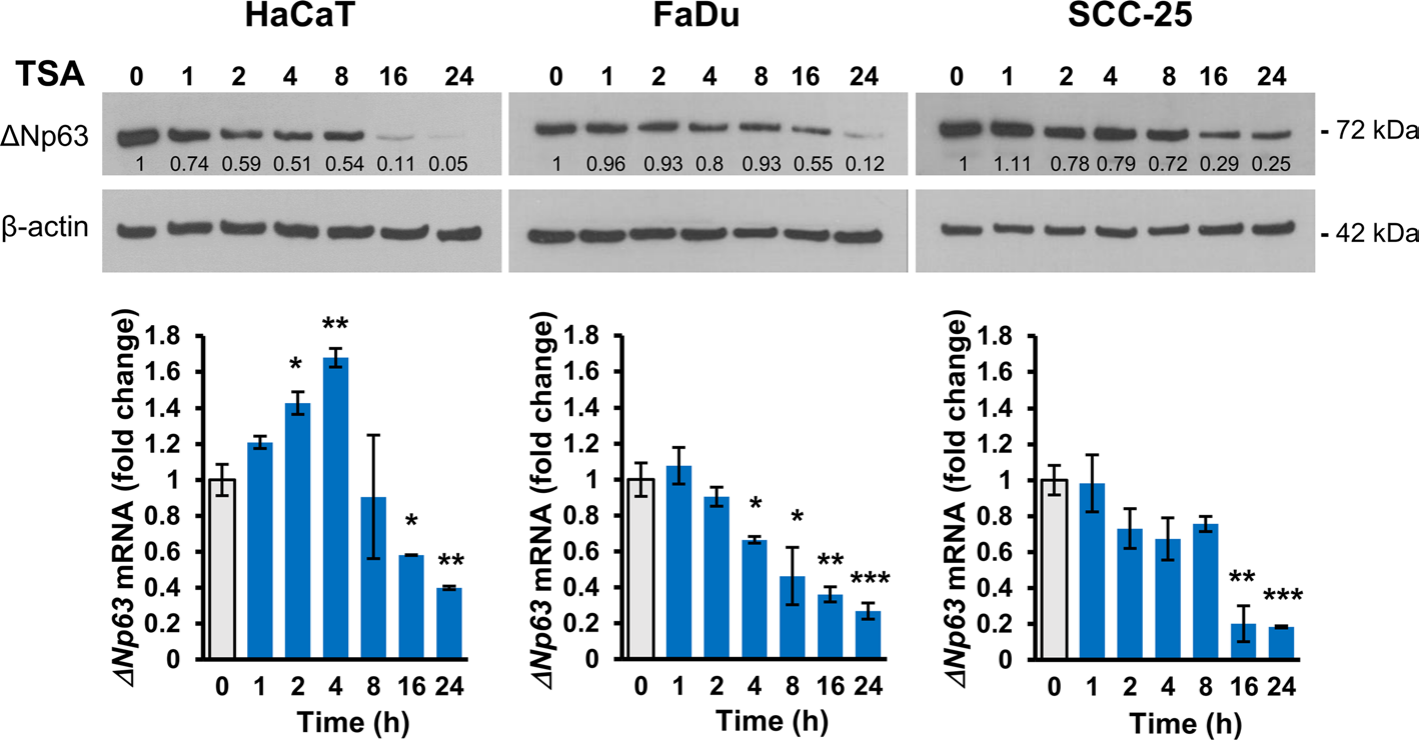
Time course of ΔNp63 response to trichostatin A Top panels; Representative western blots of ΔNp63 (72 kDa) and β-actin (42 kDa) as loading control for HaCaT, FaDu and SCC-25 cells treated with 1 µM trichostatin A (TSA) for the indicated times. Relative ΔNp63 densitometry measurements normalized to β-actin are indicated, with 0 used as control. Lower panels; RT-qPCR for *ΔNp63* mRNA shown as fold change in treated cells relative to untreated cells after normalization to *ACTB* mRNA. *p < 0.05; **p < 0.01; ***p < 0.001 compared to untreated control cells

Of the range of HDACi tested, the broad spectrum agent trichostatin A (TSA) showed the greatest effect and was therefore studied in more detail at both protein and mRNA levels. Time course experiments showed notable reduction of ΔNp63 protein from 16 h onwards (Fig. 6, upper panels), while RT-qPCR showed a biphasic time response of *ΔNp63* mRNA in HaCaT cells, with a transient increase at 4 h and 8 h before a reduction at 16 and 24 h (Fig. 6, lower panels). FaDu and SCC-25 cells did not show early induction of *ΔNp63* mRNA and showed greater downregulation at 24 h (fourfold and 5.5-fold, respectively, compared to 2.5-fold reduction in HaCaT cells at 24 h).

## Discussion

ΔNp63 overexpression is a characteristic feature of SCC, and the ability to pharmacologically reduce ΔNp63 levels would therefore be expected to have broad value for SCC patients, and for patients with other tumor types in which ΔNp63 plays an oncogenic role, such as bladder, breast and prostate cancer subtypes [11, 12, 36–38]. We confirmed that p63 knockdown inhibits the growth of HaCaT keratinocytes and malignant SCC cells, with an increased effect in the latter, providing proof of concept that reducing ΔNp63 would have value for SCC therapy. These findings are similar to observations that enhanced ΔNp63 expression causes hyperproliferation of squamous cells and leads to malignancy [39], with p63 being required for keratinocyte proliferation and for both tumor initiation and maintenance [40, 41]. Our finding that malignant FaDu cells show a more severe loss of growth potential than HaCaT keratinocytes after ΔNp63 depletion implies that SCC cells are addicted to ΔNp63 oncogenic activity, and indicates that a therapeutic window of opportunity exists for tumor-selective effects.

The regulation of ΔNp63 in normal and malignant squamous cells is multifaceted, involving cell-type dependent and interactive effects of numerous growth factors, signaling pathways, metabolism and chromatin modifications (reviewed in [11, 12, 21]). Using this knowledge, we investigated ΔNp63 regulation in SCC cells and non-tumorigenic keratinocytes to identify factors that modulate this oncogenic protein in these cells. We aimed to identify clinically available agents that modify ΔNp63 levels and are therefore potentially useful for patient therapy. We used two distinct SCC cell lines, which represent the mesenchymal (SCC-25) and the atypical molecular subtypes (FaDu), and are derived from tongue and hypopharynx, respectively [42]. These were compared to non-malignant epidermal keratinocytes that retain squamous cell differentiation potential (HaCaT). Whilst this introduces heterogeneity in terms of tissue of origin and cancer-specific genetics, which at least in part may underlie the variable effects observed, our aim was to identify agents that consistently influence ΔNp63 in SCC and would therefore be useful across sub-types by reflecting the common features of SCC, which includes ΔNp63 upregulation [1, 5]. In particular, examining three cell lines under the same conditions allowed us to make direct comparisons between cells, rather than comparing the results of previous studies that individually employed different drugs at different concentrations in different cell lines, sometimes providing different conclusions from each other.

It is known that DNA damage downregulates ΔNp63 through proteasome-mediated degradation [12, 21, 43], and we showed downregulation of ΔNp63 by multiple DNA damaging agents. Interestingly, a transient increase was observed prior to loss, which is attributed to the initial phosphorylation of ΔNp63 prior to degradation [21, 43, 44]. Thus, in addition to the direct cytotoxic action of DNA damaging agents, these therapies also decrease the pro-survival effects of ΔNp63, and ΔNp63 ablation enhances DNA damage cytotoxicity in SCC cells [16, 18, 45], whilst forced expression of ΔNp63 protects against UV-mediated keratinocyte cell death in vivo [20]. However, we also found variability in the ΔNp63 response of SCC cells (even though DNA damage levels were relatively consistent across cells as measured by phospho-H2AX), suggesting that factors such as differing levels of endogenous DNA damage response activities in SCC [46] and/or SCC-specific pathways of DNA repair [18] may be involved.

Favorable SCC growth conditions are mediated by the presence of tumor- or stromal-derived growth factors as well as activating mutations in signaling pathways such as PIK3CA or inactivation of the signaling inhibitor PTEN [1, 5, 6]. As a pro-survival factor for squamous cells, it is logical that ΔNp63 may be regulated through such pathways. Indeed, EGFR and/or PI3K activity are positively correlated with ΔNp63 [47–49] and dual activation of ΔNp63 and PI3K signaling is a hallmark of SCC [1, 6]. Whilst initial studies indicated that EGF increases ΔNp63 in SCC cells and keratinocytes through the PI3K pathway [50–52], other reports indicate that PI3K activation transcriptionally reduces ΔNp63 [53, 54]. Various other growth factors have also been linked to ΔNp63 regulation with similarly conflicting data reported [11, 12]. In our experiments, there were no consistent effects of the growth factors tested (insulin, IGF-1, EGF, KGF, and HGF) despite serum reduction before and during treatment, as used in some studies [51]. We also did not see consistent effects of wortmannin (pan PI3K inhibitor) or rapamycin (mTOR inhibitor) at concentrations that showed cytotoxicity. These results presumably reflect differing activation of these pathways in the cells used. For examples, mutation or amplification of *PIK3CA* or *EGFR*, or *PTEN* inactivation are all common in SCC [1, 6], but would be expected to show different sensitivities to exogenous stimulation or inhibition depending on the specific mutation and the combination of aberrations in growth factor signaling components [55]. Moreover, PI3K inhibitors may induce downstream targets due to feedback loops, again depending on the oncogenic status of the tumor cells [55]. On the other hand, our data confirm the potential role of EGF and identify insulin/IGF signaling in inducing or maintaining ΔNp63 levels in keratinocytes and SCC-25 cells. Most notably, the dual PI3K/mTOR inhibitor BEZ235 reduced ΔNp63 levels in all cells, confirming the involvement of PI3K/mTOR signaling. Thus, although growth factor signaling pathways play a role, the effects of pathway inhibitors on ΔNp63 are complex and depend on the cancer mutational status.

Cellular metabolism is an emerging target for cancer treatment, and there is evidence that p63 is a regulator of metabolic activity and may itself be regulated in response to metabolic imbalance [11, 12, 21]. The widely used lipid lowering drugs, statins, are also being investigated for their anti-cancer effects through their action as mevalonate pathway inhibitors [56]. Importantly, statins are not universally effective, and epithelial tumors (high E-cadherin) tend to be insensitive, whilst other tumor types are insensitive due to compensatory activation of SREBP that induces mevalonate pathway genes [56]. We found that lovastatin increased ΔNp63 protein and mRNA in HaCaT cells, with lesser effects in FaDu and SCC-25, which may relate to the findings that mevalonate pathway intermediates increase ΔNp63 in non-transformed oral squamous cells [57]. In addition, lovastatin promotes survival of oral squamous cells after genotoxic insults through inhibiting ATM and/or ATR activation [58], which are themselves responsible for DNA damage-mediated destruction of ΔNp63 [12, 21]. Cellular energy metabolism has also been linked to p63, and high doses of metformin reduce ΔNp63 in synergy with glycolysis inhibition or in combination with HDACi [32, 33]. In our experiments, metformin reduced ΔNp63 in FaDu cells under low glucose conditions, similar to previous observations in these cells [32], whereas HaCaT and SCC-25 cells showed a minimal response to metformin alone, similar to data reported in other SCC cells [33]. Taken together, these data indicate roles for both lipid metabolism and energy balance in differential regulation of ΔNp63 in keratinocytes and SCC cells. For metformin, reduction is caused by proteasomal degradation [32], whereas we show that lovastatin acts at the transcriptional level.

Acetylation of histones in gene regulatory regions is an important element of gene regulation in SCC [5, 59] and influences p63 [12]. In our experiments, inhibiting class I and II HDACs reduced ΔNp63 in a time- and dose-dependent manner. Importantly, we discovered a biphasic response, with an early increase of *ΔNp63* mRNA prior to its reduction, particularly noticeable in HaCaT cells (Fig. 6). These findings help to explain previous discrepancies that HDACi upregulated rather than downregulated ΔNp63 [34]—those experiments used short-term exposure compared to the longer exposures used here and elsewhere that decrease ΔNp63 [35, 48]. Thus, at least two pathways are in operation, with an early induction (perhaps related to direct acetylation of ΔNp63 [34]) followed by a prolonged inhibitory response that may be transcriptional by altering histone acetylation at the promoter or enhancer regions of the *ΔNp63* gene [12], or through altered regulation of other genes that influence ΔNp63 such as miRNAs [35] or PTEN [60].

## Conclusion

**Fig. 7:**
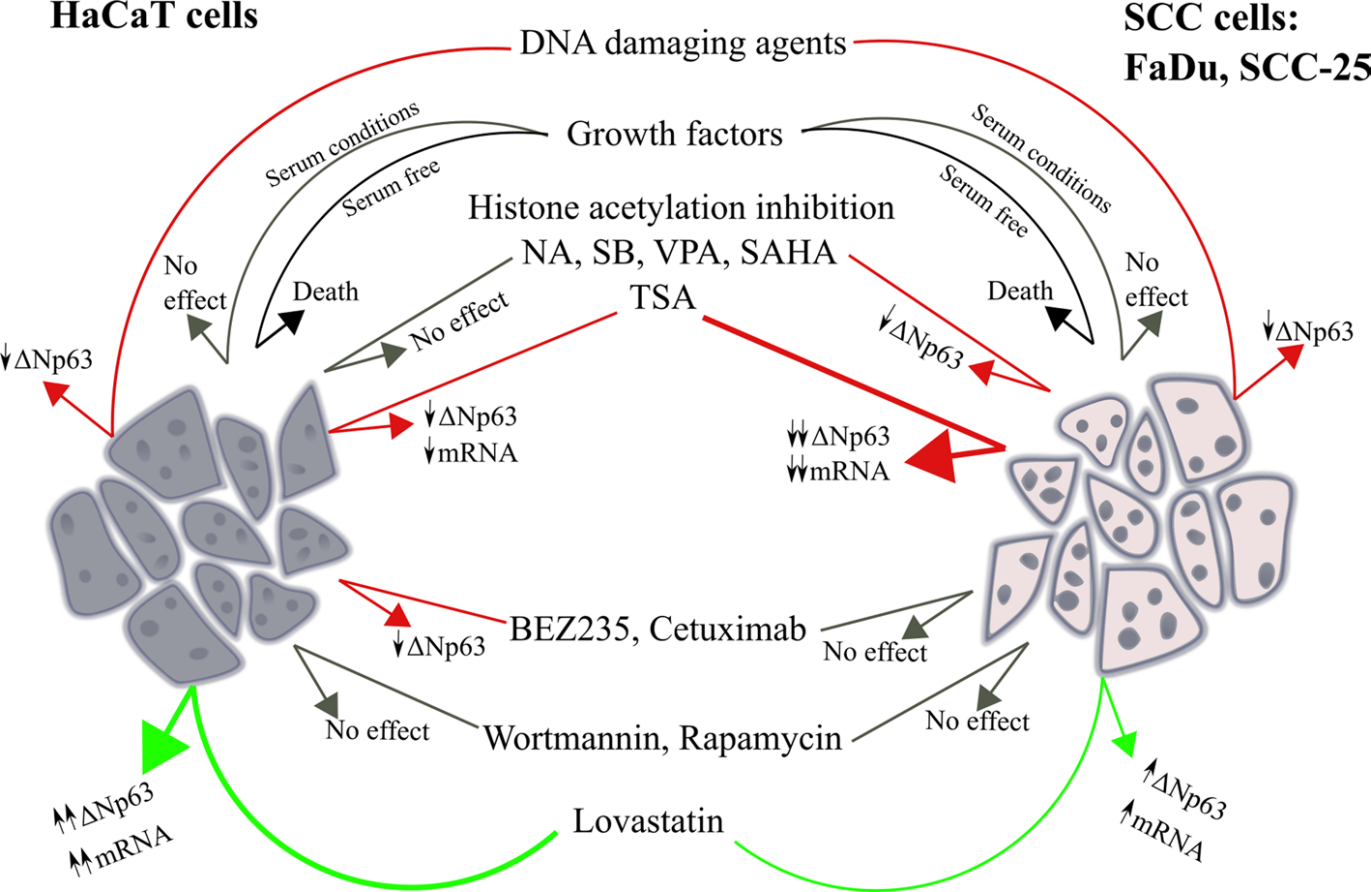
Schematic representation of ΔNp63 regulation in non-transformed HaCaT keratinocytes versus FaDu and SCC-25 SCC cell lines. Green arrows indicate positive effects, red arrows indicate negative effects and gray arrows indicate no effect on ΔNp63 levels. Note that different agents show differences in the magnitude of induction or repression of ΔNp63 between non-malignant HaCaT cells and SCC cells

SCC is a common malignancy that occurs at multiple anatomical locations and is often difficult to treat, with poor response and high mortality [1–4]. ΔNp63 has many oncogenic roles in SCC and its levels correlate with aggressiveness and therapy resistance. With respect to the latter, ΔNp63 protects cells from DNA damage-induced death and reducing ΔNp63 levels sensitizes to radiotherapy and chemotherapy [16, 18–20, 45, 61]. On the other hand, there is good clinical evidence that some chemotherapeutics are particularly effective in p63-positive tumors, indicating that ΔNp63 downregulation is a major component of their anti-cancer properties in these specific cancers [62, 63]. Thus, the ability to inhibit ΔNp63 is a logical approach to improve patient outcomes for these tumors, either as a single agent or as combination therapy. By screening known or suspected agents for their effects on ΔNp63, we identified selective cell-type dependent inducers and repressors (Fig. 7). Therefore, although these agents have many effects independent of ΔNp63 regulation, the data provide a valuable and practical framework for future investigations of pharmacologic p63 depletion, including indications for specific combination therapies dependent on tumor characteristics and mutational status reflected by molecular subtype. For example, lovastatin increased ΔNp63 levels predominantly in HaCaT cells and thus may be useful for p63-inhibition combination therapies by helping to maintain its levels in normal cells. That HDACi variably reduce ΔNp63 implies they may be useful for certain tumors and/or could be used in combination with EGFR inhibitor therapy or with metformin [33, 48]. In addition, our data show that dual AKT/mTOR inhibition is especially effective at reducing ΔNp63, but may cause more severe side-effects on normal tissues. Taken together, we demonstrate that ΔNp63 is an amenable target for manipulation in SCC by several commonly used and well-tolerated pharmaceuticals.

### Clinical implications

In summary, we have shown that ΔNp63 is required for the growth of SCC cells, confirming its’ reduction as a potential therapeutic approach for these tumors. We also show that drugs with divergent mechanisms influence ΔNp63 levels in keratinocytes and/or SCC cells. The different effects seen in different cells presumably reflect the mutational characteristics of signaling pathways in individual SCC tumors, many of which regulate and/or cooperate with ΔNp63 to promote carcinogenesis. Thus, although ΔNp63 is not their sole target, many commonly used drugs influence this protein and our data indicate that inhibiting the oncogenic p53 family member ΔNp63 is a therapeutic option for SCC.

## Supplementary Information

**Additional file 1.** Growth factors and inhibitors used in this study. Names, molecular targets, suppliers and catalog numbers of growth factors and signaling pathway inhibitors.

**Additional file 2.** Additional methods. Additional details of *TP63*-shRNA cell production and western blotting methods.

**Additional file 3.** Sequence of primers used for quantitative PCR.

**Additional file 4.** p63 depletion reduces colony cell numbers. See Fig. 1B for colony images. Quantitation of crystal violet staining of colonies of *TP63*-shRNA cells formed in the absence (Con) or presence of doxycycline (DOX) for the first four days of growth (absorbance at 570 nm after destaining). Three to six wells were assayed per clone, using two separate clones for each *TP63*-shRNA cell line. Control cells without doxycycline are designated as 100% for each clone. *p < 0.05; **p < 0.01; ***p < 0.001 comparing doxycycline treated with the corresponding control cells.

**Additional file 5.** DNA damage increases phosphorylated H2AX. To accompany Fig. 2. Western blotting of phospho-H2AX (Ser139) (15 kDa) in HaCaT, FaDu or SCC-25 cells exposed to the indicated agents. Cells were collected 24 h after treatment. β-actin (42 kDa) was used as loading control. Each cell line has individual non-treated control samples (CTR) for each genotoxic agent. Cispt, cisplatin; Etopo, etoposide; Doxo, doxorubicin.

**Additional file 6.** Downstream activation of Akt and ERK in growth factor treated cells. To accompany Fig. 3. The indicated cell lines were cultured in reduced serum medium (1% FBS) and treated with growth factors for 24 h for western blotting for phospho-Akt (Ser473) or phospho-ERK (p42/p44 MAPK (Thr202/Tyr204). β-actin was used as loading control on separate replicate gels. 0 represents untreated cells.

**Additional file 7.** The effect of inhibitors on signaling pathway activities. To accompany Fig. 3. The indicated cell lines were treated with the indicated pathway signaling inhibitors and collected 24 h later for western blotting for phospho-Akt (Ser473) or phospho-p70 S6K (Thr389). β-actin was used as loading control. 0 represents untreated cells. Wort, wortmannin; Rap, rapamycin; BEZ, BEZ-235.

**Additional file 8.** The effect of metformin on ΔNp63. To accompany Fig. 4. Western blotting of ΔNp63 in HaCaT, FaDu or SCC-25 cells exposed to the indicated concentrations of metformin. Cells were grown in low glucose medium for 16 h before treatment and collected 24 h after treatment in the same medium. β-actin was used as loading control. Relative ΔNp63 densitometry measurements normalized to β-actin are indicated, with 0 used as control.

**Additional file 9.** The effect of nicotinamide on ΔNp63. To accompany Fig. 5. Western blotting of ΔNp63 in HaCaT, FaDu or SCC-25 cells exposed to the indicated concentration of nicotinamide or without nicotinamide. Cells were collected 24 h after treatment. β-actin was used as loading control. Relative ΔNp63 densitometry measurements normalized to β-actin are indicated, with 0 used as control.

**Additional file 10.** Full western blot images.

## Data Availability

All data are contained within the article or Additional files, including raw images of western blots.

## Abbreviations

AMPK: AMP-activated kinase
Ct: Cycle threshold
DAB: Diaminobenzidine
DMEM: Dulbecco’s modified Eagle’s medium
DOX: Doxycycline
EGF: Epidermal growth factor
EGFR: EGF receptor
ERK1/2: Extracellular signal-regulated protein kinase
FBS: Fetal bovine serum
HDACi: Histone deacetylase inhibitor
HGF: Hepatocyte growth factor
HMG-CoA: 3-Hydroxy-3-methyl-glutaryl-coenzyme A
IGF: Insulin-like growth factor
KGF: Keratinocyte growth factor
MAPK: Mitogen-activated protein kinase
p70-S6 kinase: 70-KDa ribosomal protein S6 kinase
PI3K: Phoshatidyl-inositol-3-kinase
RT-qPCR: Reverse transcription and quantitative PCR
SCC: Squamous cell carcinoma
TSA: Trichostatin A
UVC: Ultraviolet light C (short wave UV)

## References

[1] Dotto GP, Rustgi AK (2016). Squamous cell cancers: a unified perspective on biology and genetics. Cancer Cell.

[2] Sung H, Ferlay J, Siegel RL, Laversanne M, Soerjomataram I, Jemal A, Bray F (2021). Global Cancer Statistics 2020: GLOBOCAN estimates of incidence and mortality worldwide for 36 cancers in 185 countries. CA Cancer J Clin.

[3] Sgaramella N, Gu X, Boldrup L, Coates PJ, Fahraeus R, Califano L, Tartaro G, Colella G, Spaak LN, Strom A (2018). Searching for new targets and treatments in the battle against squamous cell carcinoma of the head and neck, with specific focus on tumours of the tongue. Curr Top Med Chem.

[4] Johnson DE, Burtness B, Leemans CR, Lui VWY, Bauman JE, Grandis JR (2020). Head and neck squamous cell carcinoma. Nat Rev Dis Primer.

[5] Campbell JD, Yau C, Bowlby R, Liu Y, Brennan K, Fan H, Taylor AM, Wang C, Walter V, Akbani R (2018). Genomic, pathway network, and immunologic features distinguishing squamous carcinomas. Cell Rep.

[6] Cancer Genome Atlas Research Network (2012). Comprehensive genomic characterization of squamous cell lung cancers. Nature.

[7] Hibi K, Trink B, Patturajan M, Westra WH, Caballero OL, Hill DE, Ratovitski EA, Jen J, Sidransky D (2000). AIS is an oncogene amplified in squamous cell carcinoma. Proc Natl Acad Sci U S A.

[8] Massion PP, Taflan PM, Jamshedur Rahman SM, Yildiz P, Shyr Y, Edgerton ME, Westfall MD, Roberts JR, Pietenpol JA, Carbone DP (2003). Significance of P63 amplification and overexpression in lung cancer development and prognosis. Cancer Res.

[9] Redon R, Muller D, Caulee K, Wanherdrick K, Abecassis J, du Manoir S (2001). A simple specific pattern of chromosomal aberrations at early stages of head and neck squamous cell carcinomas: PIK3CA but not P63 gene as a likely target of 3q26-Qter gains. Cancer Res.

[10] Moses MA, George AL, Sakakibara N, Mahmood K, Ponnamperuma RM, King KE, Weinberg WC (2019). Molecular mechanisms of P63-mediated squamous cancer pathogenesis. Int J Mol Sci.

[11] Fisher ML, Balinth S, Mills AA (2020). P63-related signaling at a glance. J Cell Sci.

[12] Pokorná Z, Vysloužil J, Hrabal V, Vojtěšek B, Coates PJ (2021). The foggy world(s) of P63 isoform regulation in normal cells and cancer. J Pathol.

[13] Lo Muzio L, Santarelli A, Caltabiano R, Rubini C, Pieramici T, Trevisiol L, Carinci F, Leonardi R, De Lillo A, Lanzafame S (2005). P63 overexpression associates with poor prognosis in head and neck squamous cell carcinoma. Hum Pathol.

[14] Moergel M, Abt E, Stockinger M, Kunkel M (2010). Overexpression of P63 is associated with radiation resistance and prognosis in oral squamous cell carcinoma. Oral Oncol.

[15] Loljung L, Coates PJ, Nekulova M, Laurell G, Wahlgren M, Wilms T, Widlöf M, Hansel A, Nylander K (2014). High expression of P63 is correlated to poor prognosis in squamous cell carcinoma of the tongue. J Oral Pathol Med.

[16] Thurfjell N, Coates PJ, Vojtesek B, Benham-Motlagh P, Eisold M, Nylander K (2005). Endogenous P63 acts as a survival factor for tumour cells of SCCHN origin. Int J Mol Med.

[17] Rocco JW, Leong C-O, Kuperwasser N, DeYoung MP, Ellisen LW (2006). P63 mediates survival in squamous cell carcinoma by suppression of P73-dependent apoptosis. Cancer Cell.

[18] Bretz AC, Gittler MP, Charles JP, Gremke N, Eckhardt I, Mernberger M, Mandic R, Thomale J, Nist A, Wanzel M (2016). ΔNp63 activates the fanconi anemia DNA repair pathway and limits the efficacy of cisplatin treatment in squamous cell carcinoma. Nucleic Acids Res.

[19] Hao T, Gan Y-H (2020). ΔNp63α promotes the expression and nuclear translocation of PTEN, leading to cisplatin resistance in oral cancer cells. Am J Transl Res.

[20] Liefer KM, Koster MI, Wang XJ, Yang A, McKeon F, Roop DR (2000). Down-Regulation of P63 is required for epidermal UV-B-induced apoptosis. Cancer Res.

[21] Yoh K, Prywes R (2015). Pathway regulation of P63, a director of epithelial cell fate. Front Endocrinol.

[22] Rangan SR (1972). A new human cell line (FaDu) from a hypopharyngeal carcinoma. Cancer.

[23] Rheinwald JG, Beckett MA (1981). Tumorigenic keratinocyte lines requiring anchorage and fibroblast support cultured from human squamous cell carcinomas. Cancer Res.

[24] Boukamp P, Petrussevska RT, Breitkreutz D, Hornung J, Markham A, Fusenig NE (1988). Normal keratinization in a spontaneously immortalized aneuploid human keratinocyte cell line. J Cell Biol.

[25] Bankhead P, Loughrey MB, Fernández JA, Dombrowski Y, McArt DG, Dunne PD, McQuaid S, Gray RT, Murray LJ, Coleman HG (2017). QuPath: open source software for digital pathology image analysis. Sci Rep.

[26] Orzol P, Nekulova M, Holcakova J, Muller P, Votesek B, Coates PJ (2016). ΔNp63 regulates cell proliferation, differentiation, adhesion, and migration in the BL2 subtype of basal-like breast cancer. Tumour Biol J Int Soc Oncodev Biol Med..

[27] Liu Y, Nekulova M, Nenutil R, Horakova I, Appleyard MV, Murray K, Holcakova J, Galoczova M, Quinlan P, Jordan LB (2020). ∆Np63/P40 correlates with the location and phenotype of basal/mesenchymal cancer stem-like cells in human ER+ and HER2+ breast cancers. J Pathol Clin Res.

[28] Pfaffl MW (2001). A new mathematical model for relative quantification in real-time RT-PCR. Nucleic Acids Res.

[29] Nylander K, Coates PJ, Hall PA (2000). Characterization of the expression pattern of P63 alpha and delta Np63 alpha in benign and malignant oral epithelial lesions. Int J Cancer.

[30] Sethi I, Romano R-A, Gluck C, Smalley K, Vojtesek B, Buck MJ, Sinha S (2015). A Global analysis of the complex landscape of isoforms and regulatory networks of P63 in human cells and tissues. BMC Genomics.

[31] Abraham CG, Ludwig MP, Andrysik Z, Pandey A, Joshi M, Galbraith MD, Sullivan KD, Espinosa JM (2018). ΔNp63α suppresses TGFB2 expression and RHOA activity to drive cell proliferation in squamous cell carcinomas. Cell Rep.

[32] Yi Y, Chen D, Ao J, Sun S, Wu M, Li X, Bergholz J, Zhang Y, Xiao Z-X (2017). Metformin promotes AMP-activated protein kinase-independent suppression of ΔNp63α protein expression and inhibits cancer cell viability. J Biol Chem.

[33] He Y, Tai S, Deng M, Fan Z, Ping F, He L, Zhang C, Huang Y, Cheng B, Xia J (2019). Metformin and 4SC-202 synergistically promote intrinsic cell apoptosis by accelerating ΔNp63 ubiquitination and degradation in oral squamous cell carcinoma. Cancer Med.

[34] Restelli M, Molinari E, Marinari B, Conte D, Gnesutta N, Costanzo A, Merlo GR, Guerrini L (2015). FGF8, c-Abl and P300 participate in a pathway that controls stability and function of the ΔNp63α protein. Hum Mol Genet.

[35] Napoli M, Venkatanarayan A, Raulji P, Meyers BA, Norton W, Mangala LS, Sood AK, Rodriguez-Aguayo C, Lopez-Berestein G, Vin H (2016). ΔNp63/DGCR8-dependent micrornas mediate therapeutic efficacy of HDAC inhibitors in cancer. Cancer Cell.

[36] Nekulova M, Holcakova J, Coates P, Vojtesek B (2011). The role of P63 in cancer, stem cells and cancer stem cells. Cell Mol Biol Lett.

[37] Orzol P, Holcakova J, Nekulova M, Nenutil R, Vojtesek B, Coates PJ (2015). The diverse oncogenic and tumour suppressor roles of P63 and P73 in cancer: a review by cancer site. Histol Histopathol.

[38] Galoczova M, Coates P, Vojtesek B (2018). STAT3, Stem cells, cancer stem cells and P63. Cell Mol Biol Lett.

[39] Devos M, Gilbert B, Denecker G, Leurs K, Mc Guire C, Lemeire K, Hochepied T, Vuylsteke M, Lambert J, Van Den Broecke C (2017). Elevated ΔNp63α levels facilitate epidermal and biliary oncogenic transformation. J Invest Dermatol.

[40] Ramsey MR, Wilson C, Ory B, Rothenberg SM, Faquin W, Mills AA, Ellisen LW (2013). FGFR2 signaling underlies P63 oncogenic function in squamous cell carcinoma. J Clin Invest.

[41] Leonard MK, Kommagani R, Payal V, Mayo LD, Shamma HN, Kadakia MP (2011). ΔNp63α regulates keratinocyte proliferation by controlling PTEN expression and localization. Cell Death Differ.

[42] Walter V, Yin X, Wilkerson MD, Cabanski CR, Zhao N, Du Y, Ang MK, Hayward MC, Salazar AH, Hoadley KA (2013). Molecular subtypes in head and neck cancer exhibit distinct patterns of chromosomal gain and loss of canonical cancer genes. PLoS ONE.

[43] Armstrong SR, Wu H, Wang B, Abuetabh Y, Sergi C, Leng RP (2016). The regulation of tumor suppressor P63 by the ubiquitin-proteasome system. Int J Mol Sci.

[44] Bamberger C, Pankow S, Yates JR (2021). SMG1 and CDK12 Link ΔNp63α phosphorylation to RNA surveillance in keratinocytes. J Proteome Res.

[45] Prieto-Garcia C, Hartmann O, Reissland M, Fischer T, Maier CR, Rosenfeldt M, Schülein-Völk C, Klann K, Kalb R, Dikic I (2021). Inhibition of USP28 overcomes cisplatin-resistance of squamous tumors by suppression of the fanconi anemia pathway. Cell Death Differ.

[46] Rentoft M, Laurell G, Coates PJ, Sjöström B, Nylander K (2009). Gene expression profiling of archival tongue squamous cell carcinomas provides sub-classification based on DNA repair genes. Int J Oncol.

[47] Holcakova J, Nekulova M, Orzol P, Nenutil R, Podhorec J, Svoboda M, Dvorakova P, Pjechova M, Hernychova L, Vojtesek B (2017). ΔNp63 activates EGFR signaling to induce loss of adhesion in triple-negative basal-like breast cancer cells. Breast Cancer Res Treat.

[48] Citro S, Bellini A, Miccolo C, Ghiani L, Carey TE, Chiocca S (2019). Synergistic antitumour activity of HDAC inhibitor SAHA and EGFR inhibitor gefitinib in head and neck cancer: a key role for ΔNp63α. Br J Cancer.

[49] Segrelles C, Moral M, Lara MF, Ruiz S, Santos M, Leis H, García-Escudero R, Martínez-Cruz AB, Martínez-Palacio J, Hernández P (2006). Molecular determinants of Akt-induced keratinocyte transformation. Oncogene.

[50] Matheny KE, Barbieri CE, Sniezek JC, Arteaga CL, Pietenpol JA (2003). Inhibition of epidermal growth factor receptor signaling decreases P63 expression in head and neck squamous carcinoma cells. Laryngoscope.

[51] Barbieri CE, Barton CE, Pietenpol JA (2003). Delta Np63 alpha expression is regulated by the phosphoinositide 3-kinase pathway. J Biol Chem.

[52] Ripamonti F, Albano L, Rossini A, Borrelli S, Fabris S, Mantovani R, Neri A, Balsari A, Magnifico A, Tagliabue E (2013). EGFR through STAT3 modulates ΔN63α expression to sustain tumor-initiating cell proliferation in squamous cell carcinomas. J Cell Physiol.

[53] Hu L, Liang S, Chen H, Lv T, Wu J, Chen D, Wu M, Sun S, Zhang H, You H (2017). ΔNp63α is a common inhibitory target in oncogenic PI3K/Ras/Her2-induced cell motility and tumor metastasis. Proc Natl Acad Sci U S A.

[54] Yoh KE, Regunath K, Guzman A, Lee S-M, Pfister NT, Akanni O, Kaufman LJ, Prives C, Prywes R (2016). Repression of P63 and Induction of EMT by mutant Ras in mammary epithelial cells. Proc Natl Acad Sci U S A.

[55] Thorpe LM, Yuzugullu H, Zhao JJ (2015). PI3K in cancer: divergent roles of isoforms, modes of activation and therapeutic targeting. Nat Rev Cancer.

[56] Longo J, van Leeuwen JE, Elbaz M, Branchard E, Penn LZ (2020). Statins as anticancer agents in the era of precision medicine. Clin Cancer Res.

[57] Scheller EL, Baldwin CM, Kuo S, D’Silva NJ, Feinberg SE, Krebsbach PH, Edwards PC (2011). Bisphosphonates inhibit expression of P63 by oral keratinocytes. J Dent Res.

[58] Ziegler V, Albers A, Fritz G (2016). Lovastatin protects keratinocytes from DNA damage-related pro-apoptotic stress responses stimulated by anticancer therapeutics. Biochim Biophys Acta.

[59] Castilho RM, Squarize CH, Almeida LO (2017). Epigenetic modifications and head and neck cancer: implications for tumor progression and resistance to therapy. Int J Mol Sci.

[60] Pan L, Lu J, Wang X, Han L, Zhang Y, Han S, Huang B (2007). Histone deacetylase inhibitor trichostatin a potentiates doxorubicin-induced apoptosis by up-regulating PTEN expression. Cancer.

[61] Ogawa E, Okuyama R, Ikawa S, Nagoshi H, Egawa T, Kurihara A, Yabuki M, Tagami H, Obinata M, Aiba S (2008). P51/P63 inhibits ultraviolet B-induced apoptosis via Akt activation. Oncogene.

[62] Zangen R, Ratovitski E, Sidransky D (2005). DeltaNp63alpha levels correlate with clinical tumor response to cisplatin. Cell Cycle Georget Tex.

[63] Rocca A, Viale G, Gelber RD, Bottiglieri L, Gelber S, Pruneri G, Ghisini R, Balduzzi A, Pietri E, D’Alessandro C (2008). Pathologic complete remission rate after cisplatin-based primary chemotherapy in breast cancer: correlation with P63 expression. Cancer Chemother Pharmacol.

