# Supplementary material for: Identifying pathways regulating the oncogenic p53 family member ΔNp63 provides therapeutic avenues for squamous cell carcinoma
Source: Cell Mol Biol Lett. 2022 Feb 23;27:18. doi: 10.1186/s11658-022-00323-x (PMC8903560; doi:10.1186/s11658-022-00323-x)
Supplement: Supplementary file 1 — Additional file 1. Growth factors and inhibitors used in this study. Names, molecular targets, suppliers and catalog numbers of growth factors and signaling pathway inhibitors. [file 11658_2022_323_MOESM1_ESM.docx]

**Additional file 1.** Growth factors and inhibitors used in this study

| **Agent** | **Mechanism of action** | **Catalog number, Company** |
| --- | --- | --- |
| Amphiregulin | EGFR agonist | A7080, Sigma Aldrich |
| BEZ235 (dactolisib) | PI3K/mTOR inhibitor | S1009, Selleckchem |
| Cetuximab | EGFR inhibitor | Cetuximab 5mg/ml, Erbitux |
| CAL-101 (idelalisib) | PI3K-delta subunit inhibitor | HY-13026 Medchem Express |
| Cisplatin | DNA damage | Cisplatin 1mg/ml, Ebewe |
| Doxorubicin | DNA damage/ topoisomerase inhibitor | doxorubicin hydrochloride 2mg/ml, TEVA |
| EGF | EGFR agonist | AF-100-15, Peprotech |
| Etoposide | DNA damage/  topoisomerase 2 poison | Ebewe 200mg |
| HGF | c-Met agonist | 100-39H, Peprotech |
| IGF-1 | IGFR and IR agonist | 100-11, Peprotech |
| Insulin | IR and IGF1R agonist | Humulin R 100IU/ml, Lilly |
| KGF | FGFR2b agonist | 100-19, Peprotech |
| Lovastatin | HMG-CoA reductase | 1530/10, Tocris Biotechne |
| Nicotinamide | Sirtuin inhibitor | N0636, Sigma Aldrich |
| Rapamycin | mTOR1/2 inhibitor | BML-A275-005, Enzo life sciences |
| SAHA | HDACi | 4652/10, Tocris Biotechne |
| SB202190 | p38 MAP kinase inhibitor | Sigma Aldrich |
| Sodium butyrate | HDACi | TR-1008-6, Millipore |
| Trichostatin A | HDACi | T-8552, Sigma Aldrich |
| Valproic acid | HDACi | P4543, Sigma Aldrich |
| Wortmannin | PI3K inhibitor | HY-10197, Medchem Express |
