# Supplementary material for: Identifying pathways regulating the oncogenic p53 family member ΔNp63 provides therapeutic avenues for squamous cell carcinoma
Source: Cell Mol Biol Lett. 2022 Feb 23;27:18. doi: 10.1186/s11658-022-00323-x (PMC8903560; doi:10.1186/s11658-022-00323-x)
Supplement: Supplementary file 2 — Additional file 2. Additional methods. Additional details of TP63-shRNA cell production and western blotting methods. [file 11658_2022_323_MOESM2_ESM.docx]

Inducible *TP63* knockdown cell lines

Plasmid DNAs were isolated using a Plasmid Maxi Kit (12162, Qiagen, USA) and used to produce viral particles in HEK293FT cells according to the manufacturer’s protocol. Viruses were collected 2 days after transfection and used to transduce HaCaT, FaDu and SCC-25 cells. Medium was replaced after 24 h, and selection in puromycin (1µg/ml) was started after a further 24 h growth. Medium containing puromycin was replaced every three days for two to three weeks until resistant cells emerged. Puromycin-resistant cells were expanded, shRNAs were induced with 1 µg/ml doxycycline for 24 h and ΔNp63 was assessed by western blotting of each cell line containing one of the three shRNA sequences, comparing cells with and without doxycycline. Cell populations showing p63 downregulation were single-cell cloned (BD FACS Aria III, Berks., UK) and at least two individual clones were prepared for each cell line after growth in puromycin-containing medium. Individual clones were re-tested by western blotting for efficient p63 knockdown after doxycycline induction. Stable cell lines containing inducible *TP63*-shRNAs were routinely cultured in DMEM with 10% FBS and 1 µg/ml puromycin for shRNA construct maintenance. To induce shRNA-mediated depletion, doxycycline was added at 1 µg/ml final concentration and the medium was replaced with freshly prepared medium every 24 h.

To determine proliferation rates after depletion, cells were seeded onto sterile 8-well slides (Ibidi Gmbh, Grafelfing, Germany) for the time required for cell adhesion (10-16 h depending on cell type). Doxycycline or control medium was then added for 4 days. Cells were fixed with cold methanol/acetone (50/50) for 10 min, allowed to dry and incubated with blocking buffer (Dako Agilent, Santa Clara, CA, USA) for 30 min at room temperature, followed by overnight incubation at 4 °C with mouse monoclonal anti-Ki67 antigen (MIB-1 M7240 Dako) diluted 1:250 (0.18 µg/ml) in blocking buffer. After three washes in PBS, cells were incubated with Envision peroxidase-polymer labeled anti-mouse Ig (Dako) for 30 min and DAB was used as the chromogen. Cells were counterstained with hematoxylin, dehydrated, cleared, and mounted in Entellan for bright-field microscopy. Ki67 positivity was automatically quantified using QuPath image analysis with default settings for hematoxylin/DAB and a detection threshold of 0.25 for all images, with 3 to 5 images (more than 1200 cells) used for each clone.

For colony forming ability measurements, *TP63*-shRNA cells were detached by trypsin, resuspended in medium and 250 single cells/well were flow-sorted into six-well plates in triplicate. After adherence, cells were cultured with or without 1 µg/ml doxycycline for 4 days before culture without doxycycline. Colonies that formed after 10 days for FaDu or after 2 weeks for HaCaT were stained with crystal violet (0.5% w/v in 20% methanol) and the number of colonies was manually counted in each well. After counting and photography, colonies were destained in 1% SDS and the amount of dissolved crystal violet from each well was determined by absorbance at 570 nm.

Western blotting

Cells were washed three times with cold phosphate-buffered saline (PBS) before harvesting into NET lysis buffer (150 mM NaCl, 1% NP-40, 50 mM Tris pH 8.0, 50 mM NaF, 5 mM EDTA supplemented with protease and phosphatase inhibitors (Thermo Fisher Scientific, USA) and containing freshly prepared PMSF. Samples were diluted with 4x complete sample buffer (CSB, 62.5 mM Tris-HCl pH 6.8, 2% SDS, 0.4% glycerol, 0.04% bromophenol blue, 5% β-mercaptoethanol) and heated at 95 °C for 5 min before separation on 10% polyacrylamide gels and transfer to nitrocellulose membranes. Membranes were cut into upper and lower portions to be used for ΔNp63 and β-actin detection, respectively, and blocked with 5% non-fat milk in PBS with 0.1% Tween-20 (PBS/T) for 90 min at room temperature. After incubation with primary antibodies, membranes were washed three times in PBS/T and once in PBS and incubated with peroxidase-coupled goat anti-mouse IgG or goat anti-rabbit IgG (Jackson Immunoresearch, West Grove, PA, USA) diluted 1:5000 for 1 h at room temperature. After washing in PBS/T and PBS, bands were visualized on X-ray film using enhanced chemiluminescence (ECL, Amersham Pharmacia Biotech, Bucks, UK).
