# Supplementary material for: Identifying pathways regulating the oncogenic p53 family member ΔNp63 provides therapeutic avenues for squamous cell carcinoma
Source: Cell Mol Biol Lett. 2022 Feb 23;27:18. doi: 10.1186/s11658-022-00323-x (PMC8903560; doi:10.1186/s11658-022-00323-x)
Supplement: Supplementary file 3 — Additional file 3. Sequence of primers used for quantitative PCR. [file 11658_2022_323_MOESM3_ESM.docx]

**Additional file 3.** Sequence of primers used for quantitative PCR.

| Primer | Sequence |
| --- | --- |
| *ACTB* F | 5 ́-GCC GAC AGG ATG CAG AAG GAG-3 ́ |
| *ACTB* R | 5 ́-CTA GAA GCA TTT GCG GTG GAC-3 ́ |
| *ΔNp63* F | 5 ́-AGC CAG AAG AAA GGA CAG CA-3 ́ |
| *ΔNp63* R | 5 ́-TCA CTA AAT TGA GTC TGG GCA T-3 ́ |
