# Supplementary figures and images for: Identifying pathways regulating the oncogenic p53 family member ΔNp63 provides therapeutic avenues for squamous cell carcinoma
Source: Cell Mol Biol Lett. 2022 Feb 23;27:18. doi: 10.1186/s11658-022-00323-x (PMC8903560; doi:10.1186/s11658-022-00323-x)

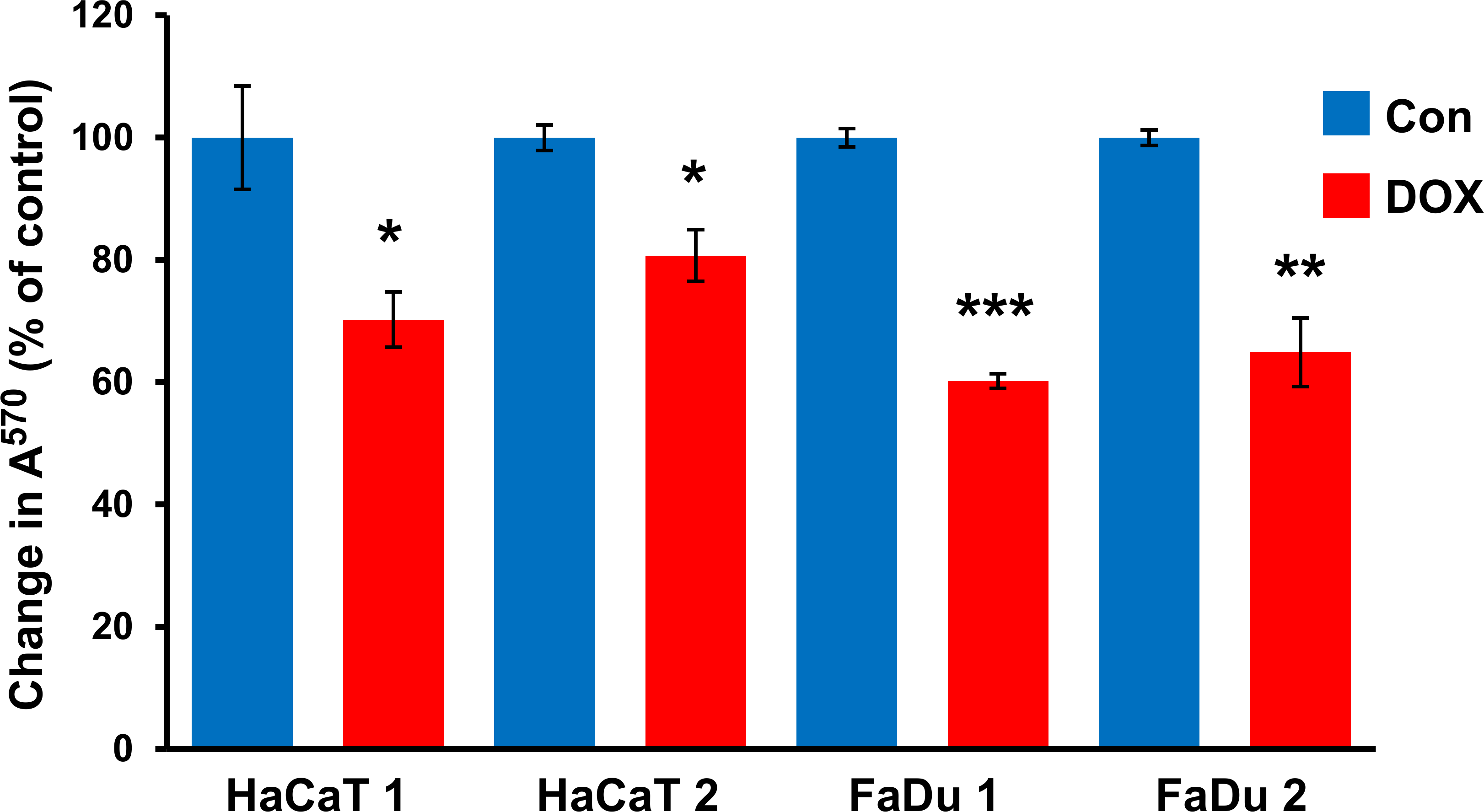

Supplement: Supplementary file 4 — Additional file 4. p63 depletion reduces colony cell numbers. See Fig. 1B for colony images. Quantitation of crystal violet staining of colonies of TP63-shRNA cells formed in the absence (Con) or presence of doxycycline (DOX) for the first four days of growth (absorbance at 570 nm after destaining). Three to six wells were assayed per clone, using two separate clones for each TP63-shRNA cell line. Control cells without doxycycline are designated as 100% for each clone. *p < 0.05; **p < 0.01; ***p < 0.001 comparing doxycycline treated with the corresponding control cells. [file 11658_2022_323_MOESM4_ESM.tif]

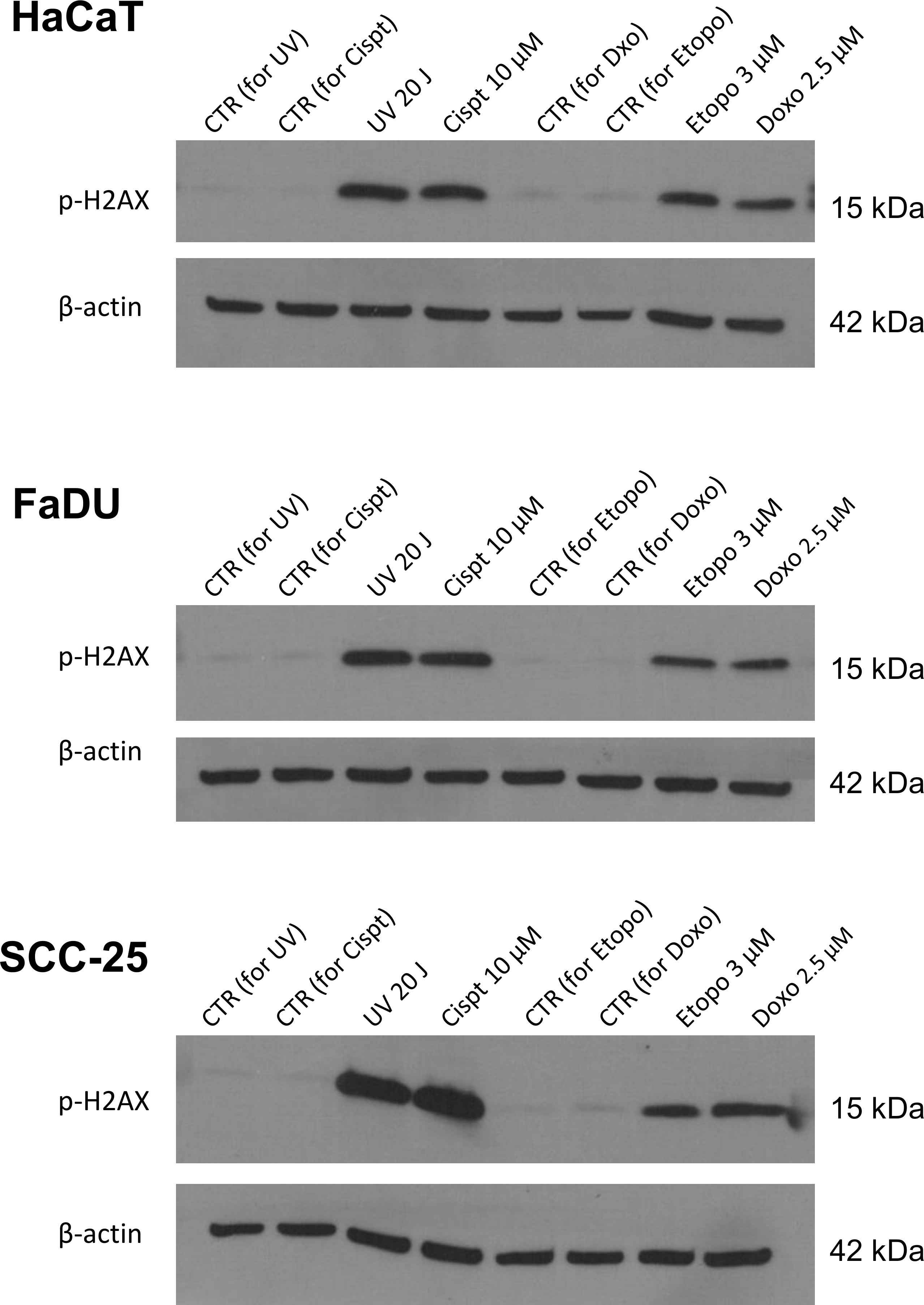

Supplement: Supplementary file 5 — Additional file 5. DNA damage increases phosphorylated H2AX. To accompany Fig. 2. Western blotting of phospho-H2AX (Ser139) (15 kDa) in HaCaT, FaDu or SCC-25 cells exposed to the indicated agents. Cells were collected 24 h after treatment. β-actin (42 kDa) was used as loading control. Each cell line has individual non-treated control samples (CTR) for each genotoxic agent. Cispt, cisplatin; Etopo, etoposide; Doxo, doxorubicin. [file 11658_2022_323_MOESM5_ESM.tif]

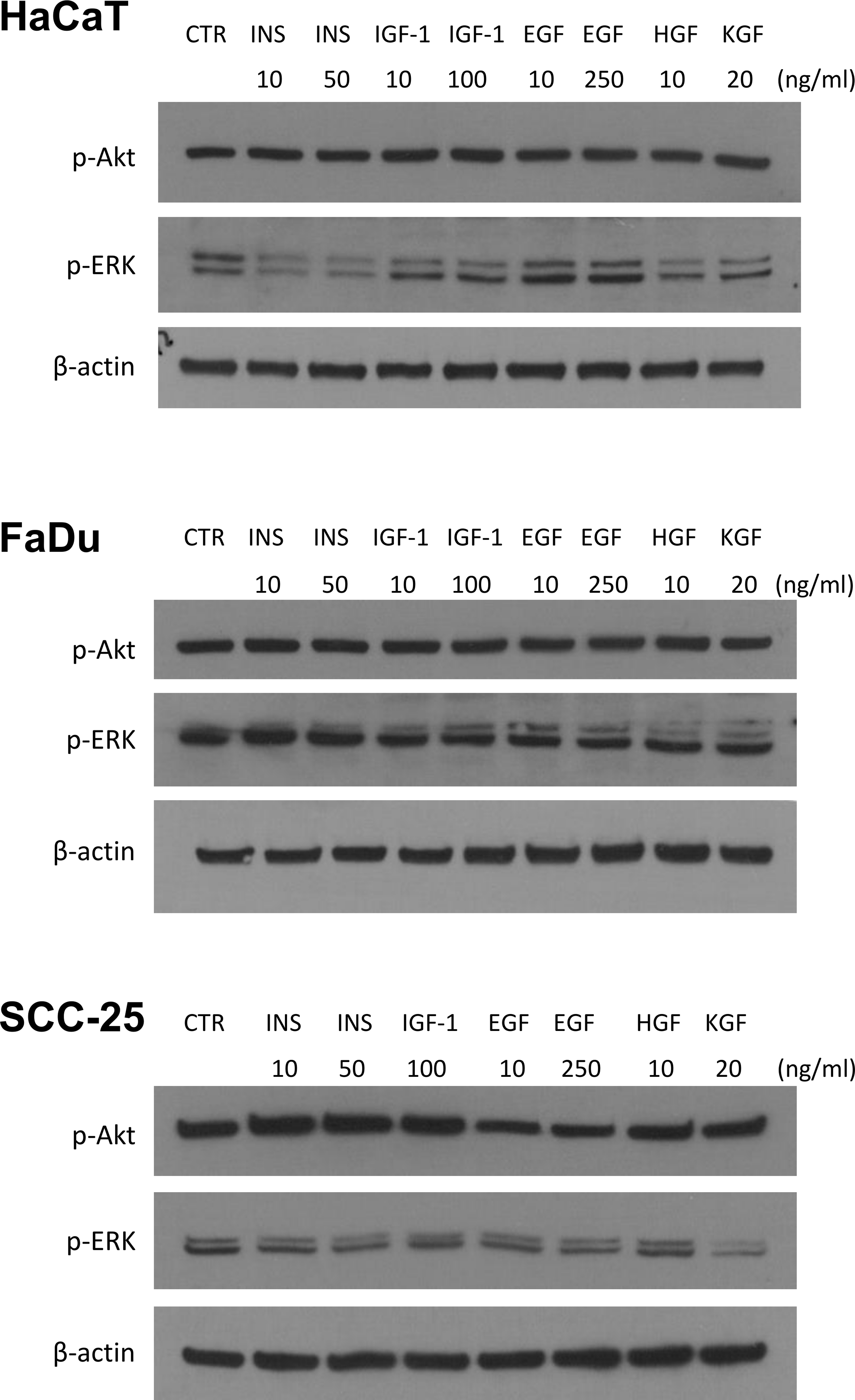

Supplement: Supplementary file 6 — Additional file 6. Downstream activation of Akt and ERK in growth factor treated cells. To accompany Fig. 3. The indicated cell lines were cultured in reduced serum medium (1% FBS) and treated with growth factors for 24 h for western blotting for phospho-Akt (Ser473) or phospho-ERK (p42/p44 MAPK (Thr202/Tyr204). β-actin was used as loading control on separate replicate gels. 0 represents untreated cells. [file 11658_2022_323_MOESM6_ESM.tif]

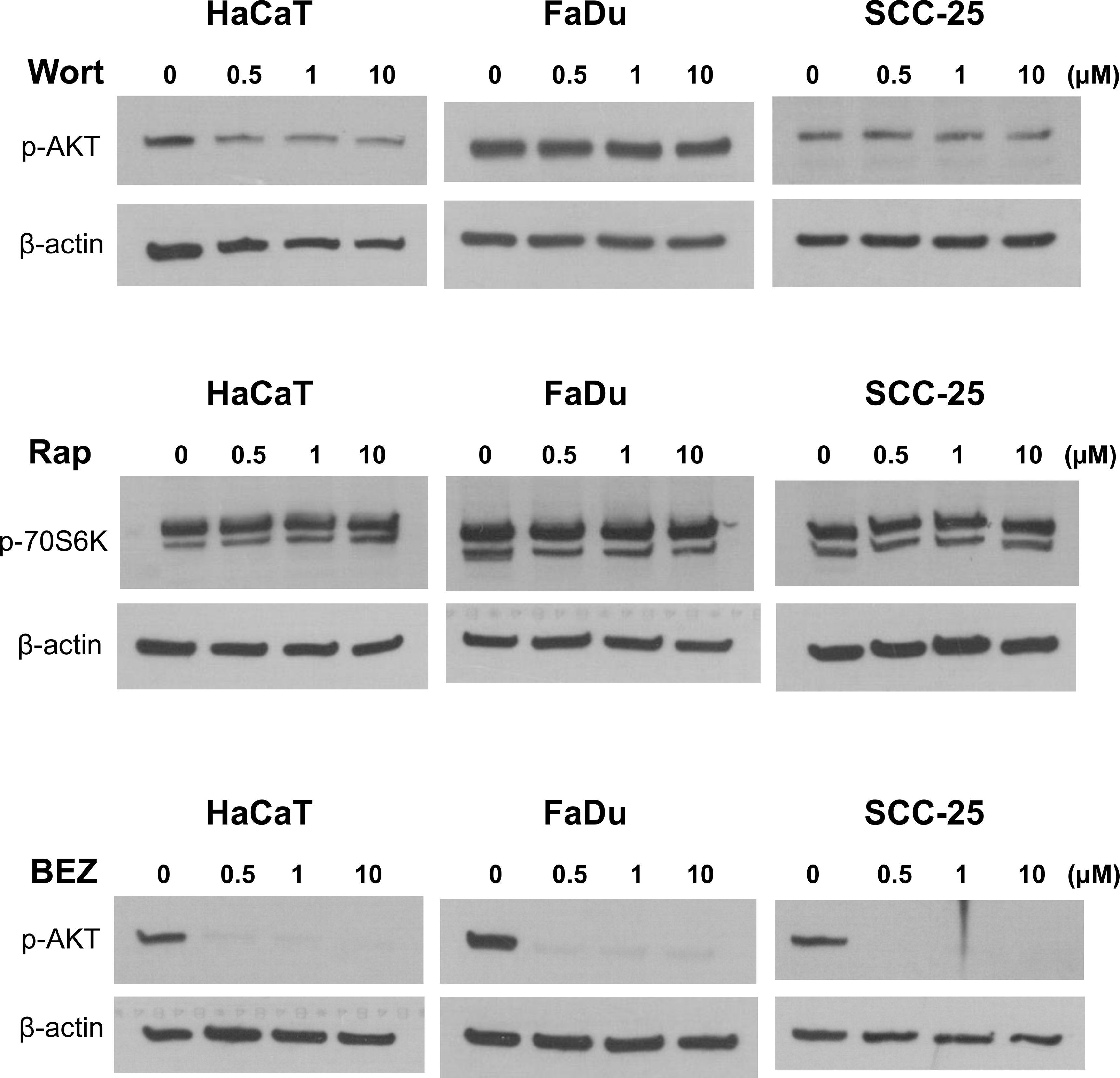

Supplement: Supplementary file 7 — Additional file 7. The effect of inhibitors on signaling pathway activities. To accompany Fig. 3. The indicated cell lines were treated with the indicated pathway signaling inhibitors and collected 24 h later for western blotting for phospho-Akt (Ser473) or phospho-p70 S6K (Thr389). β-actin was used as loading control. 0 represents untreated cells. Wort, wortmannin; Rap, rapamycin; BEZ, BEZ-235. [file 11658_2022_323_MOESM7_ESM.tif]

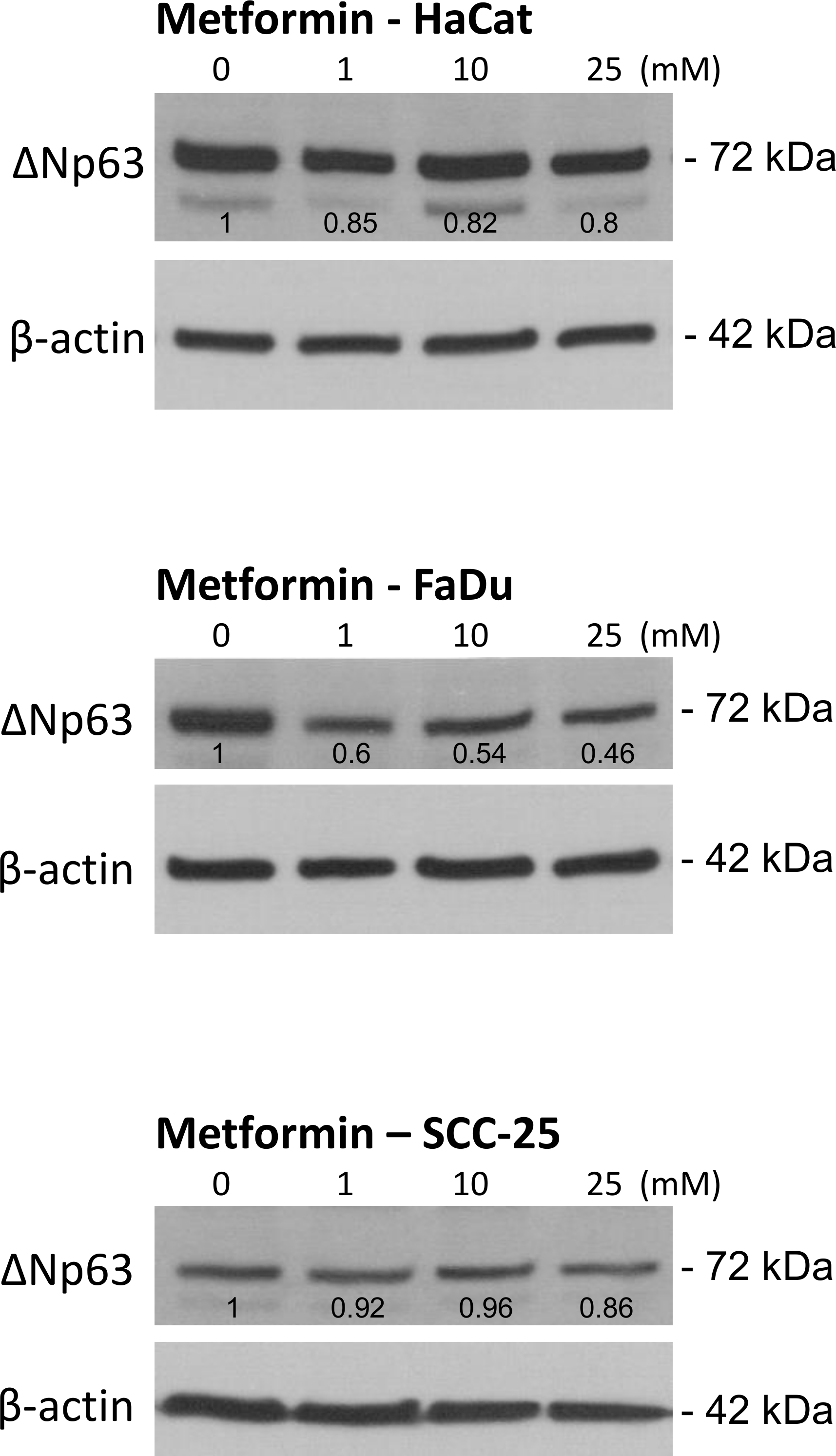

Supplement: Supplementary file 8 — Additional file 8. The effect of metformin on ΔNp63. To accompany Fig. 4. Western blotting of ΔNp63 in HaCaT, FaDu or SCC-25 cells exposed to the indicated concentrations of metformin. Cells were grown in low glucose medium for 16 h before treatment and collected 24 h after treatment in the same medium. β-actin was used as loading control. Relative ΔNp63 densitometry measurements normalized to β-actin are indicated, with 0 used as control. [file 11658_2022_323_MOESM8_ESM.tif]

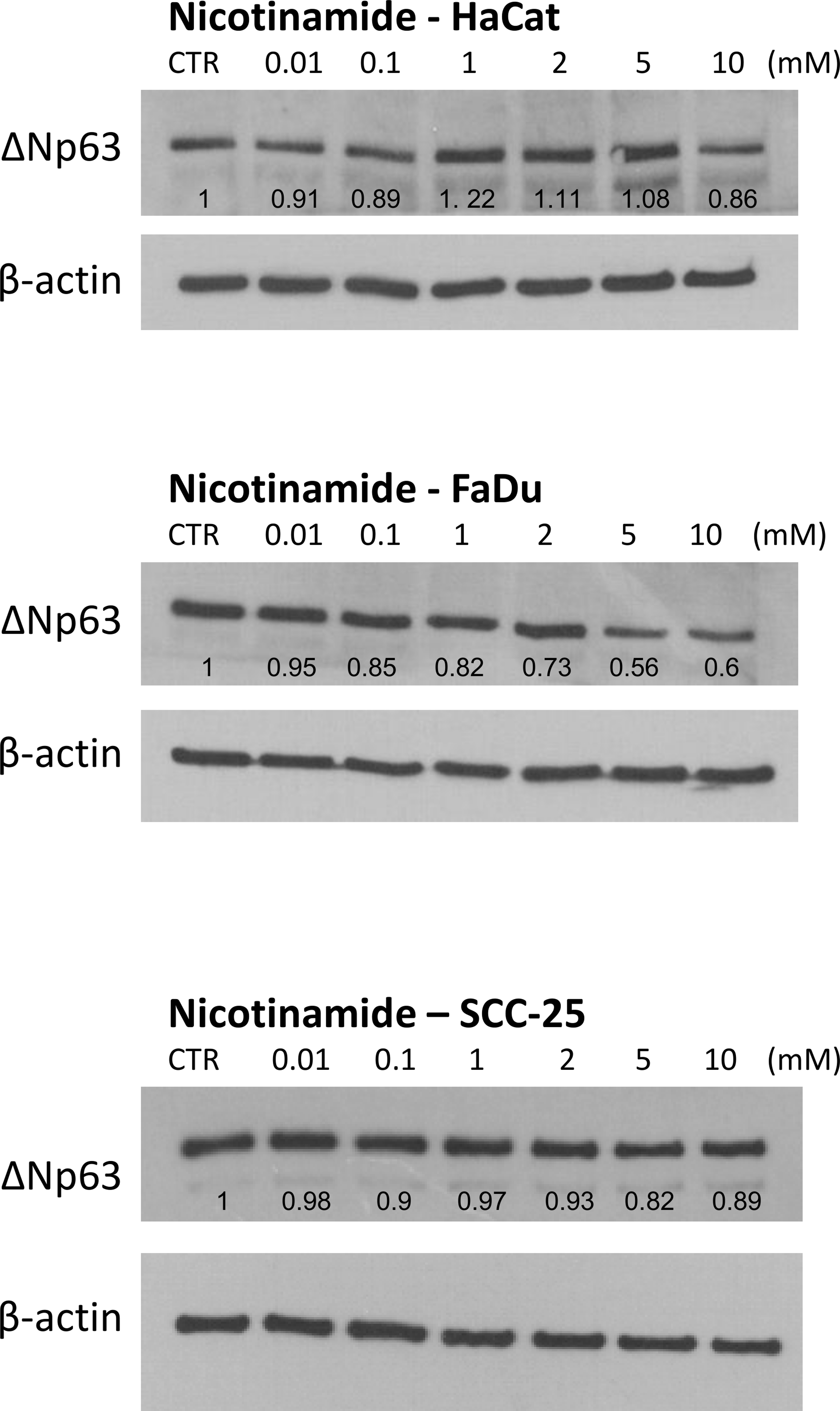

Supplement: Supplementary file 9 — Additional file 9. The effect of nicotinamide on ΔNp63. To accompany Fig. 5. Western blotting of ΔNp63 in HaCaT, FaDu or SCC-25 cells exposed to the indicated concentration of nicotinamide or without nicotinamide. Cells were collected 24 h after treatment. β-actin was used as loading control. Relative ΔNp63 densitometry measurements normalized to β-actin are indicated, with 0 used as control. [file 11658_2022_323_MOESM9_ESM.tif]
