# Supplementary material for: Identifying pathways regulating the oncogenic p53 family member ΔNp63 provides therapeutic avenues for squamous cell carcinoma
Source: Cell Mol Biol Lett. 2022 Feb 23;27:18. doi: 10.1186/s11658-022-00323-x (PMC8903560; doi:10.1186/s11658-022-00323-x)

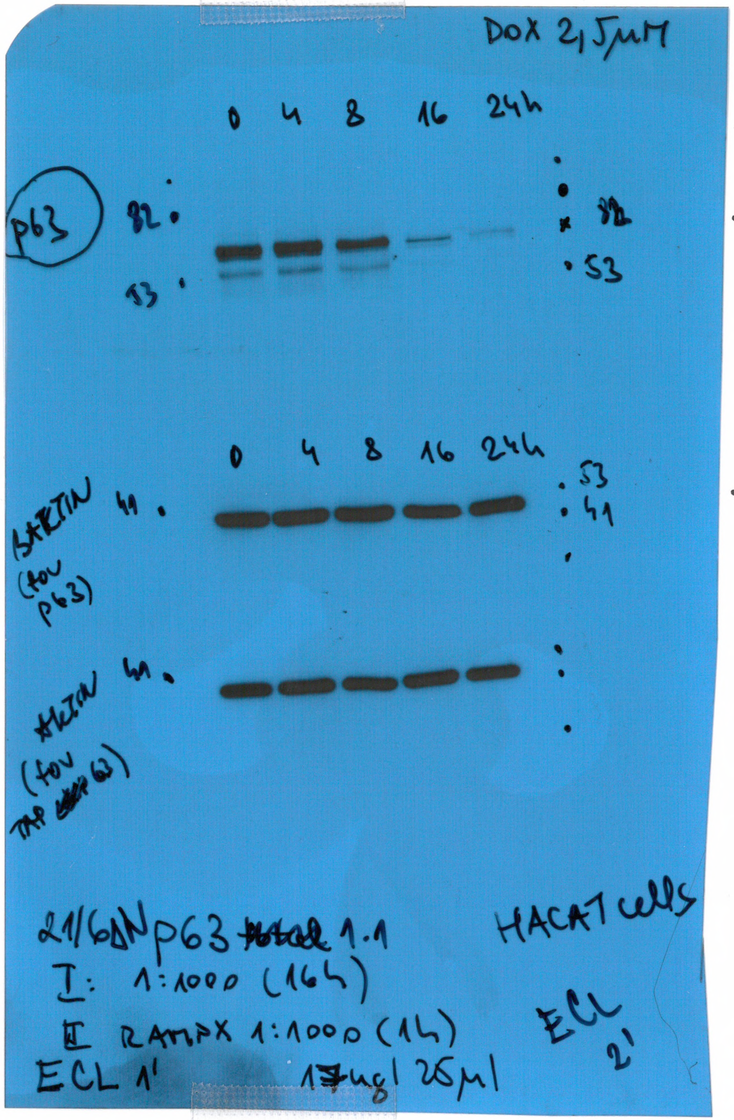

← HACAT, DOXORUBICIN (0-24h)
17µg/25µl
ΔNp63 1.1 1:1000

← β-actin 1:1000

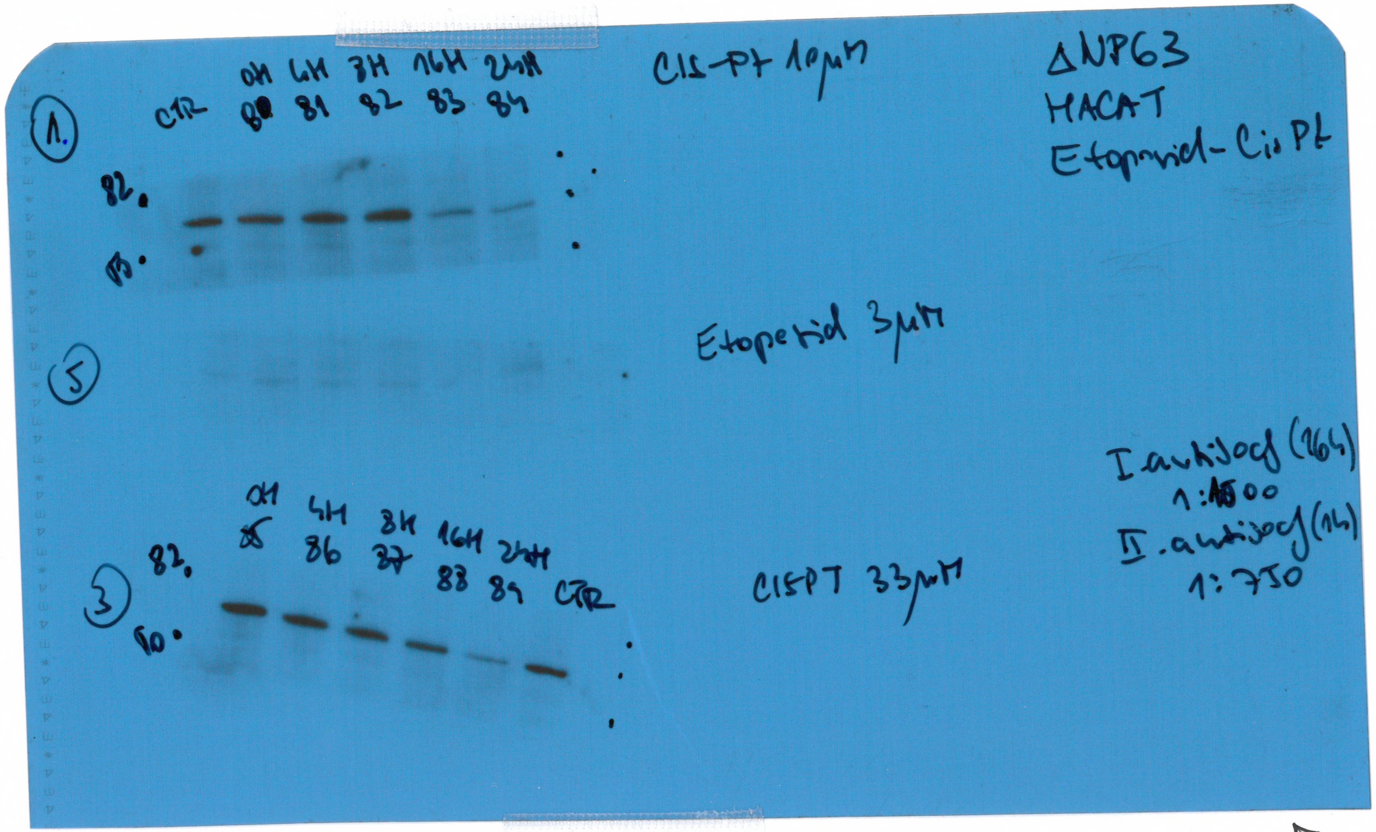

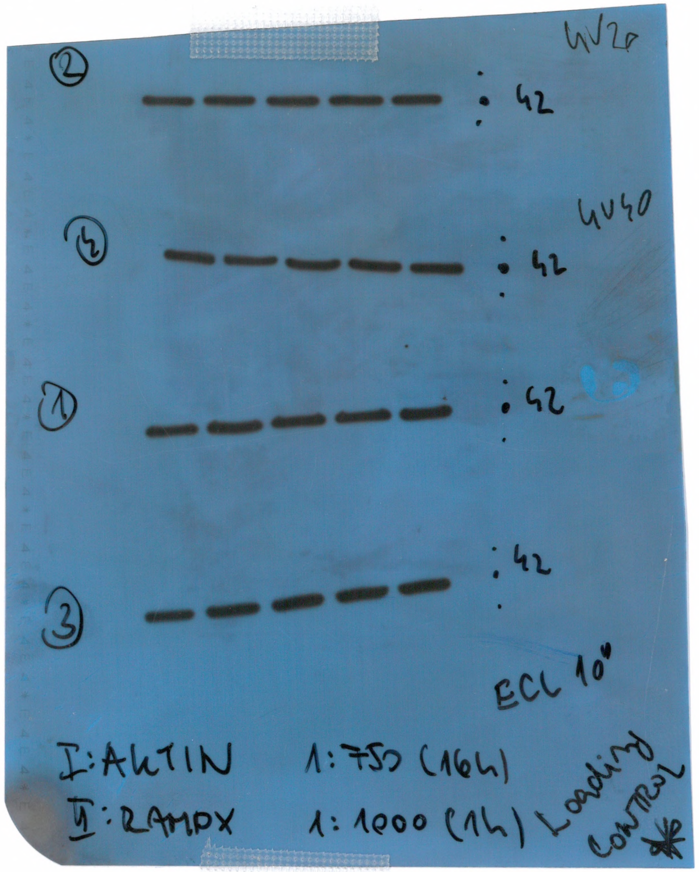

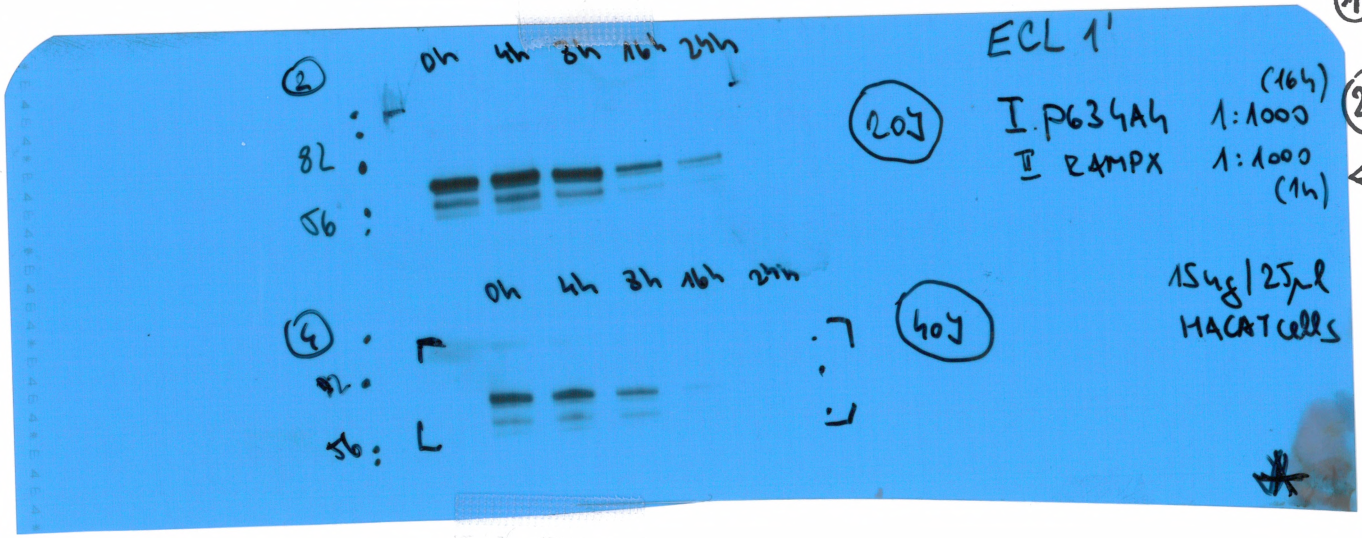

① HACAT, Cisplatin 10μM (0-24h)
ΔNp63 1.1 1:/1000

② HACAT, 20J (0-24h)
← ΔNp63 1.1 1:1000

8/4/2021

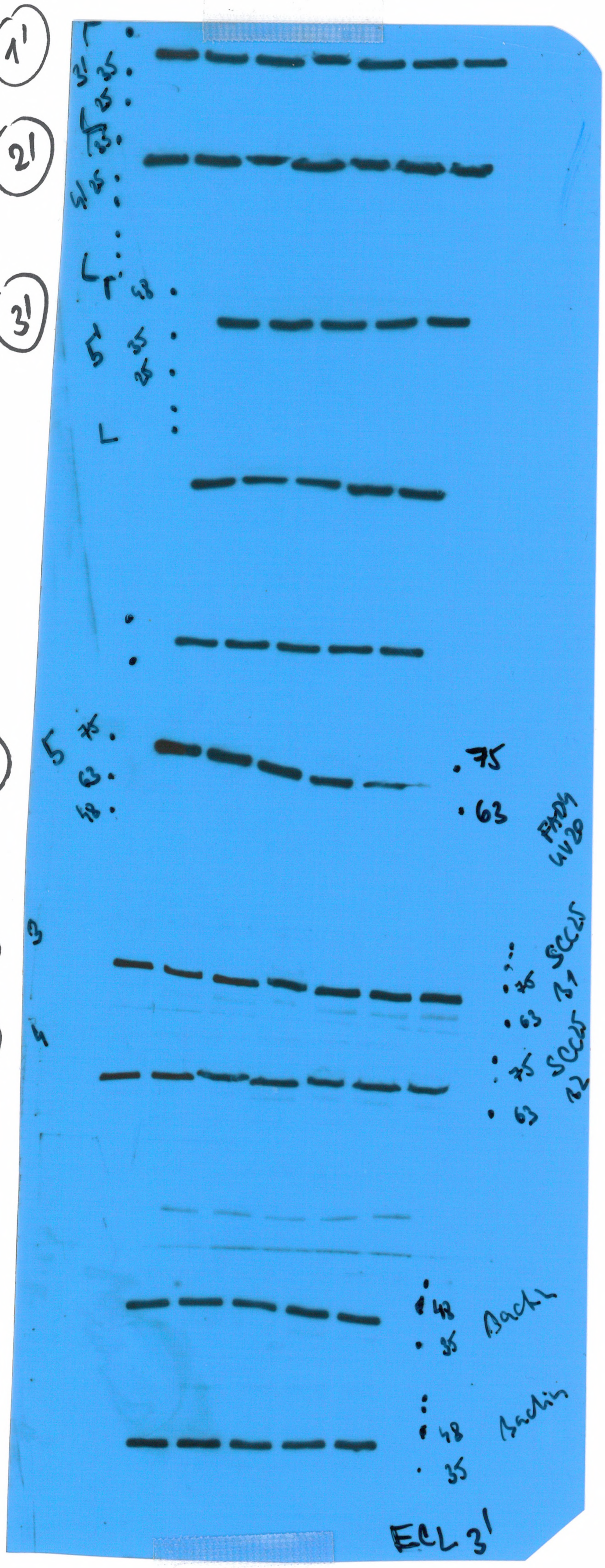

SCC-25 (TGFβ1), β-ACTIN

SCC-25 (TGFβ2), β-ACTIN

FADU (UV20), β-ACTIN

FADU (UV20) 30μg ΔNp63 1.1 1:1000

SCC-25 (TGFβ1), 25μg ΔNp63 1.1 1:1000

SCC-25 (TGFβ2), 25μg ΔNp63 1.1 1:1000

P-H2AX

B-ACTIN

30µg 33µl

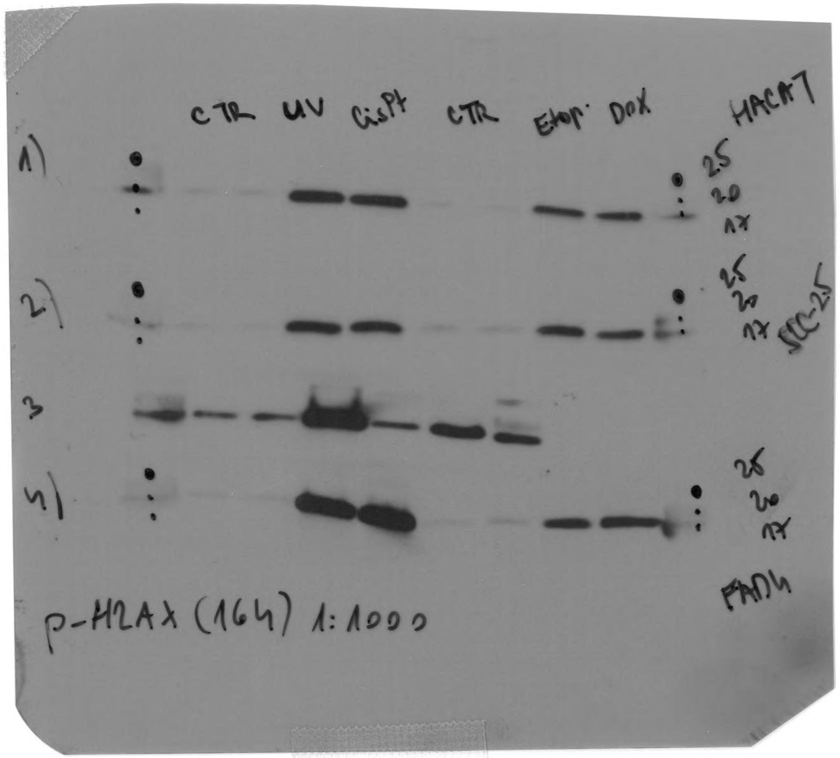

ECL1'

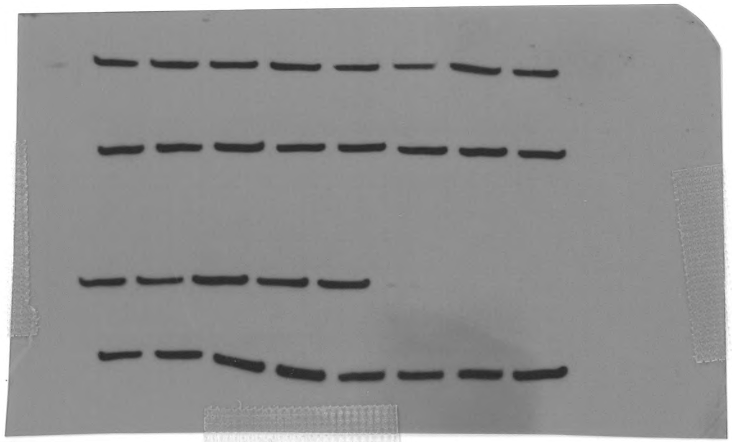

ECL1'

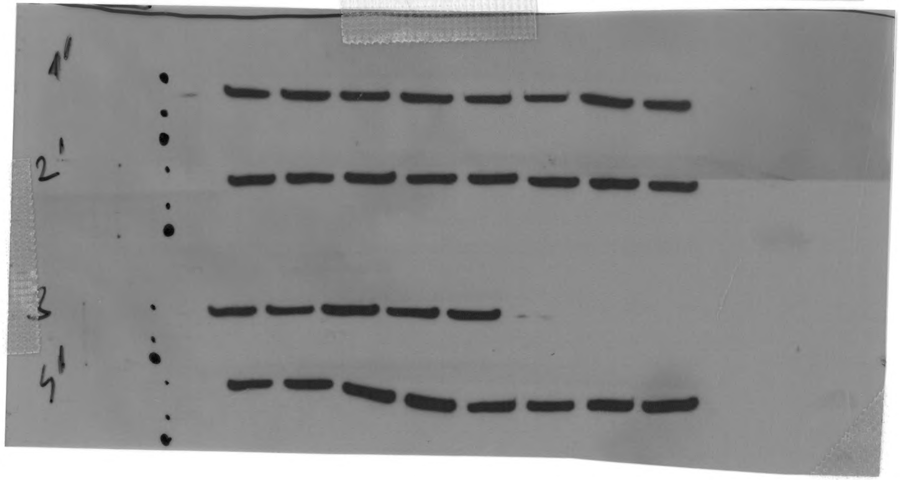

ECL3'

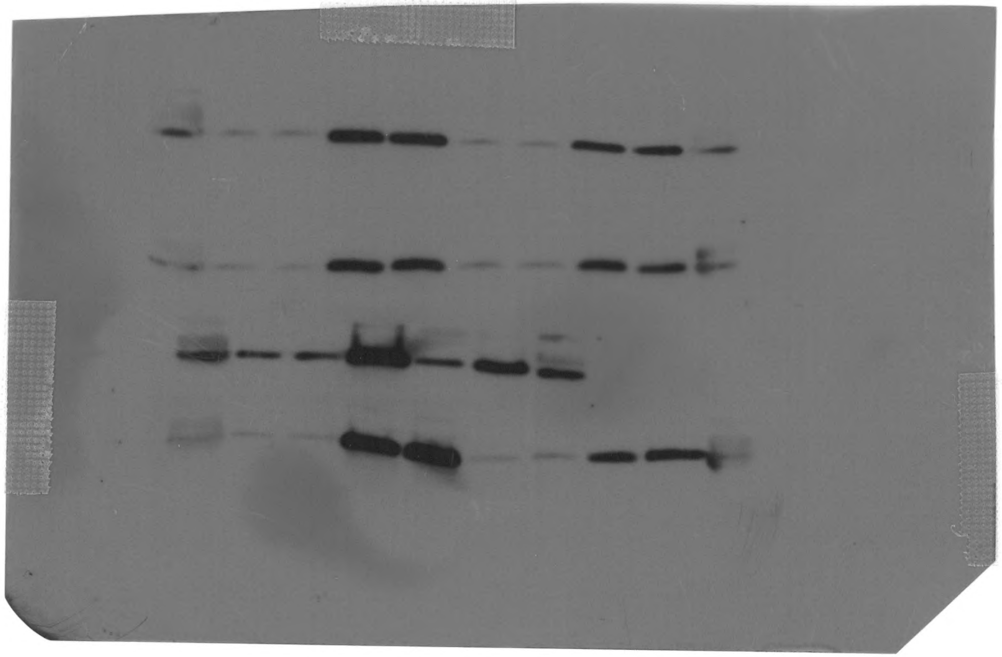

ECL3'

1,2,4
HACAT, FADU, SCC-25 genotoxic insult

1- SCC-25, WORTMANNIN
2, 3, 4 GENOTOXIC INSULT SCC-25

ΔNp63 1:1 1:500

7/9/21

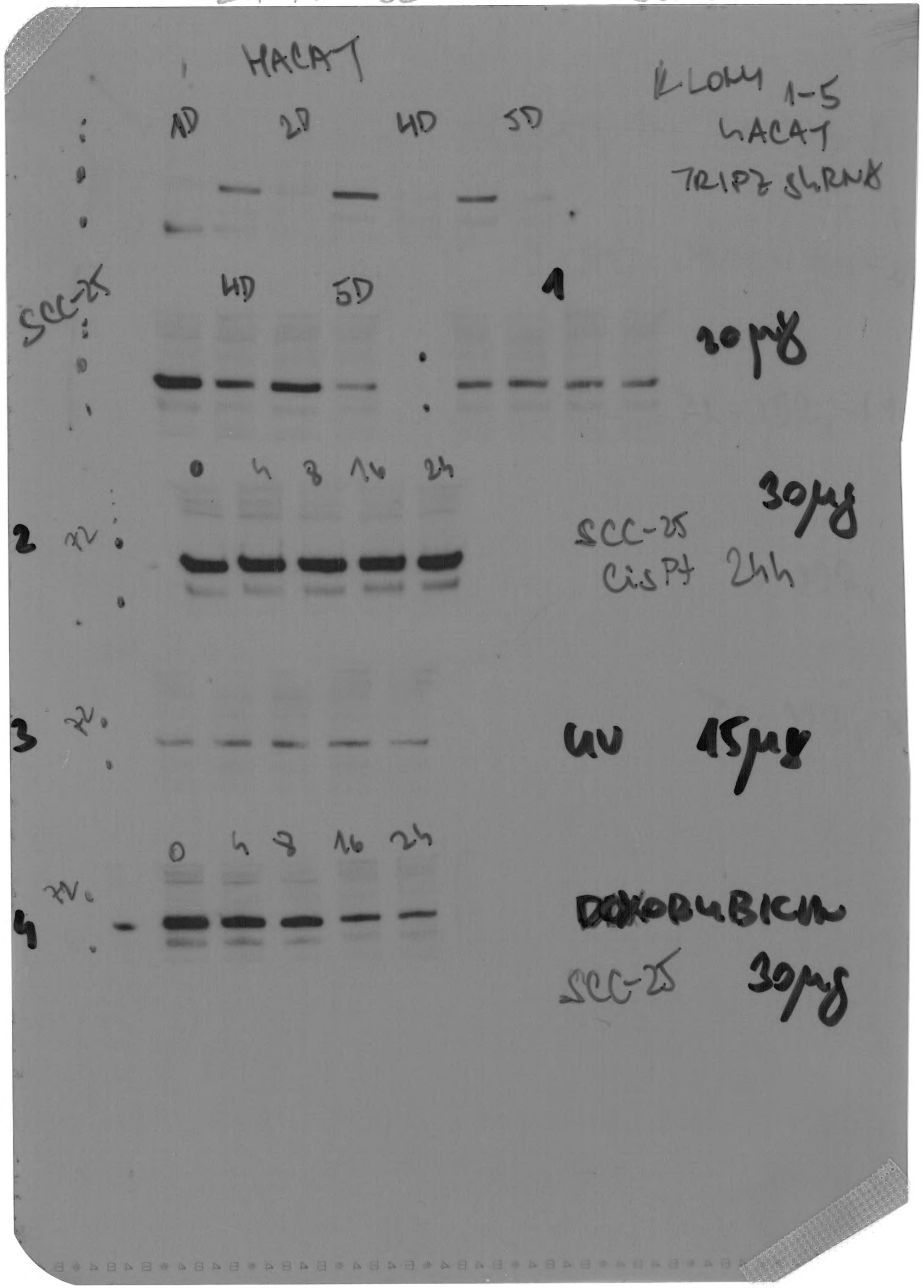

ECL 1'

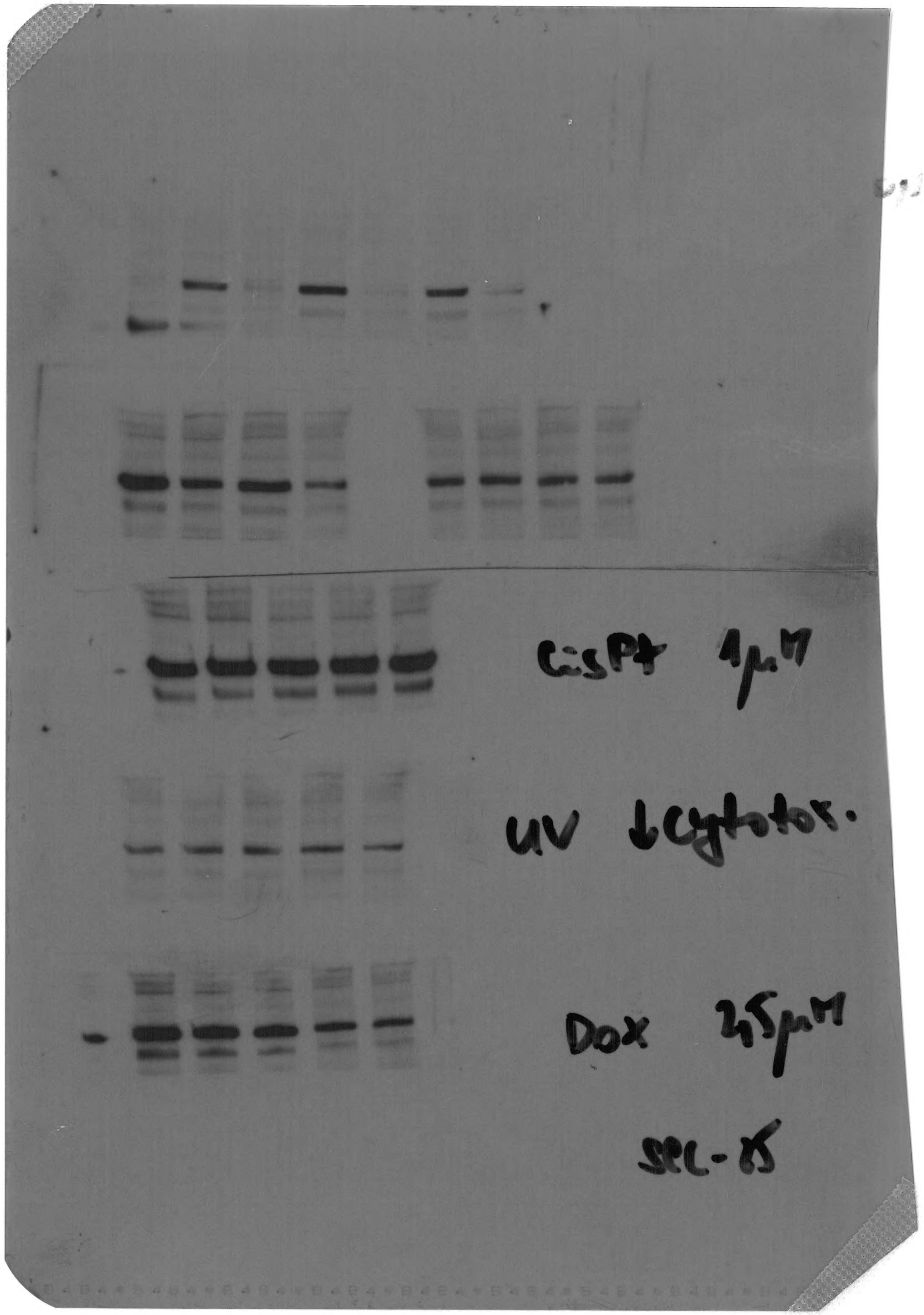

ECL 5'

7/9/21

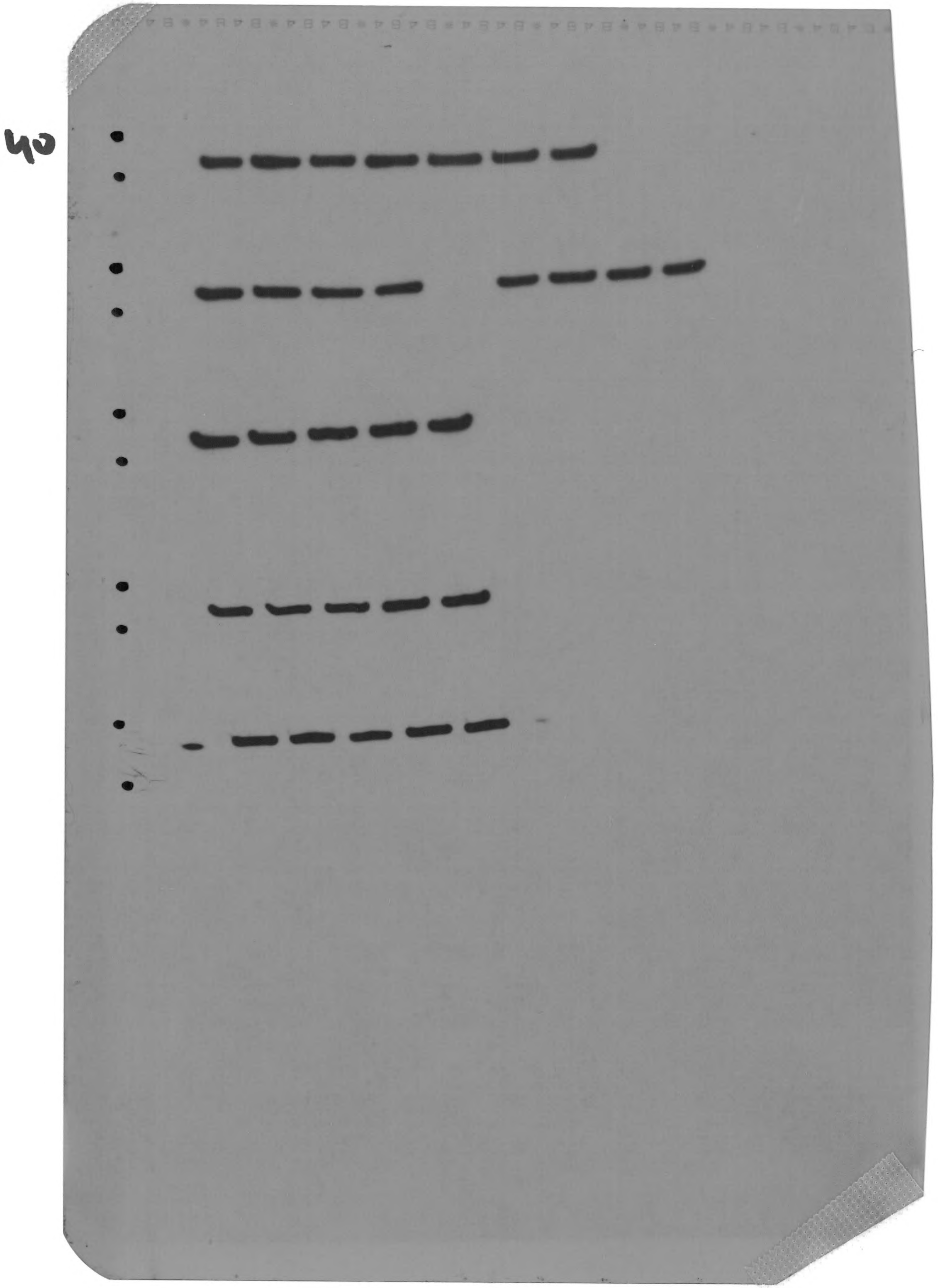

B-ACTIN 1:1000

WORTMANNIN, SCC-25

CisPt, SCC-25

UV, SCC-25

DOX, SCC-25

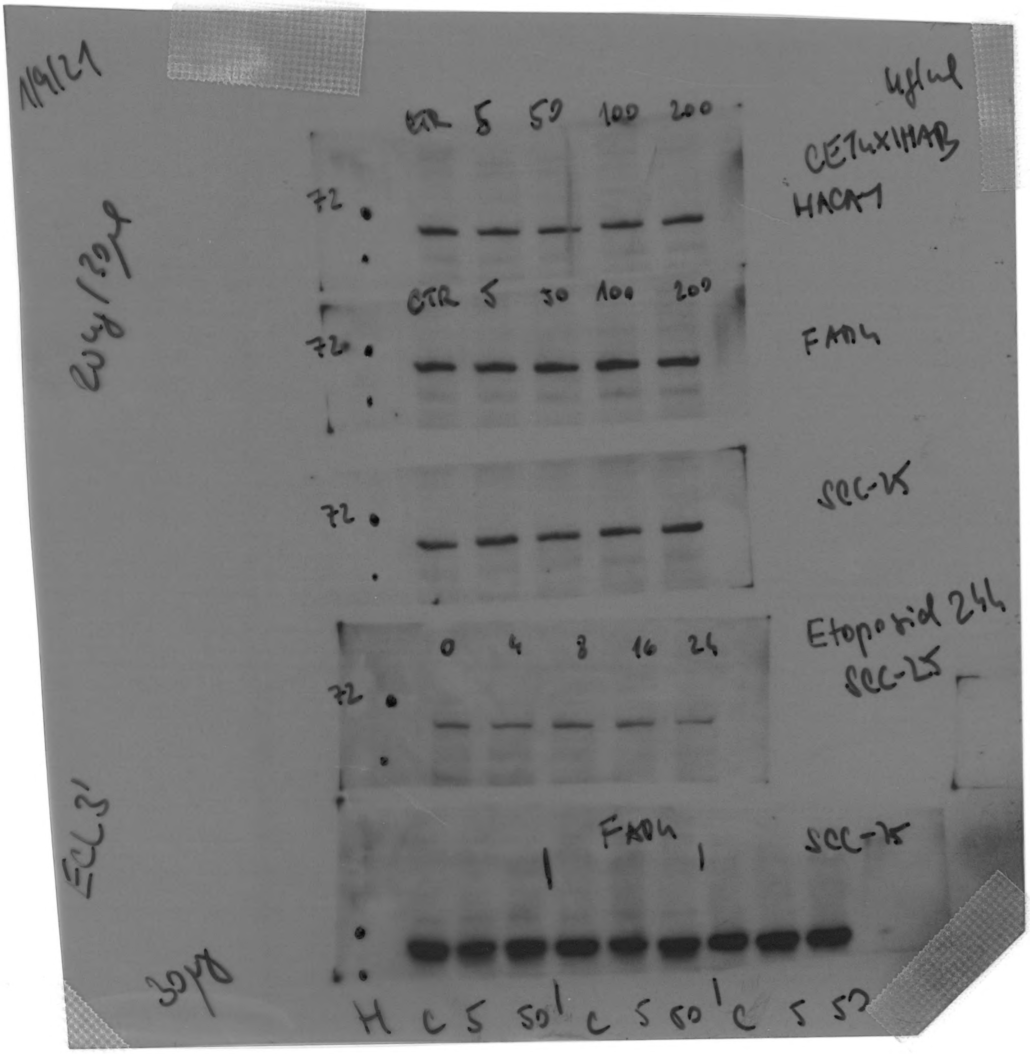
1/9/21
ug/ml
CTR 5 50 100 200
CETUXIMAB
HACAT
72
20µg/30µg
CTR 5 30 100 200
FADU
SCC-25
Etoposid 24h
SCC-25
0 4 8 16 24
FADU
SCC-25
ECL 3'
30µg
H C 5 50 C 5 50 C 5 50

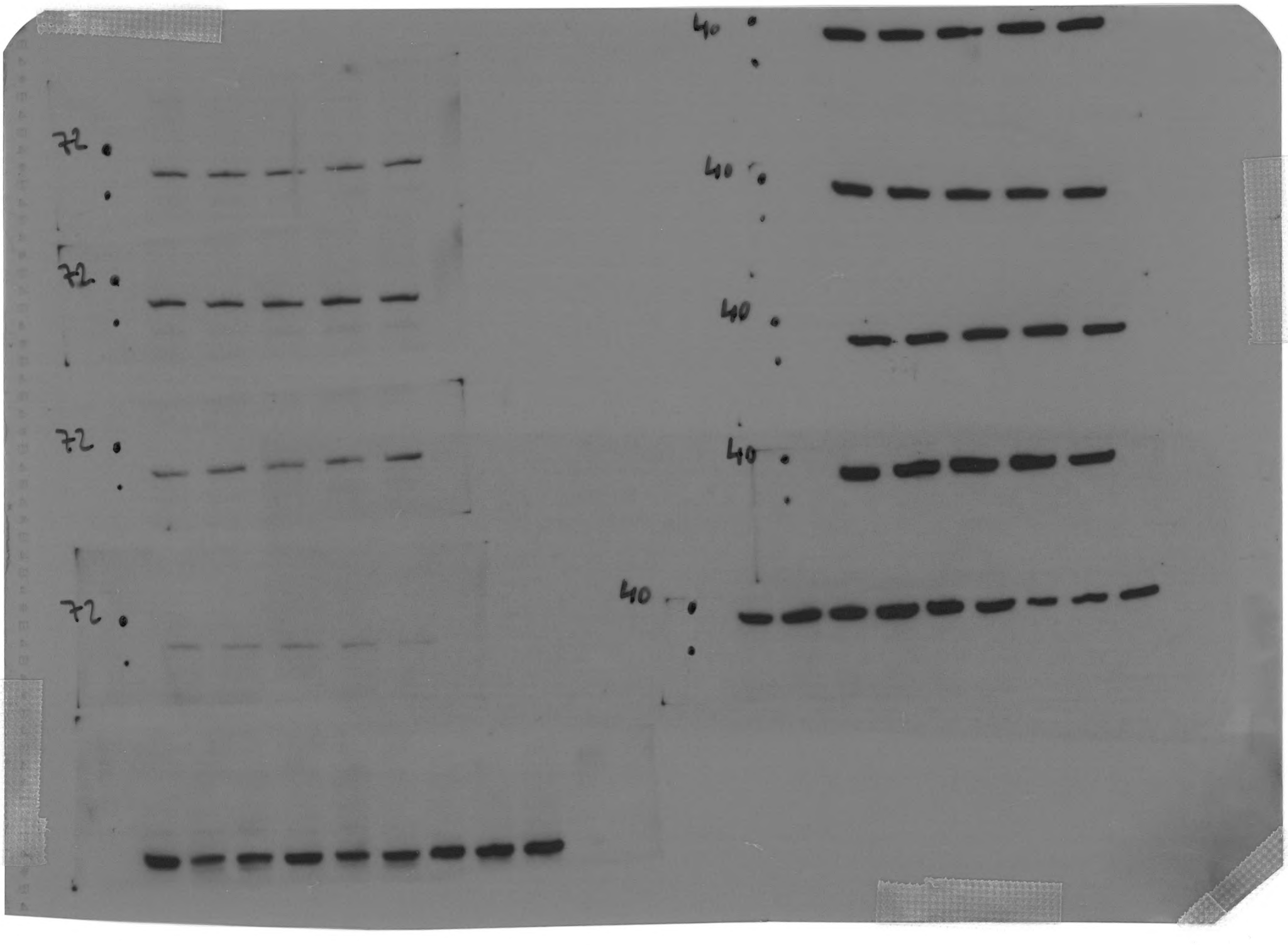
72
72
72
72
40
40
40
40
40

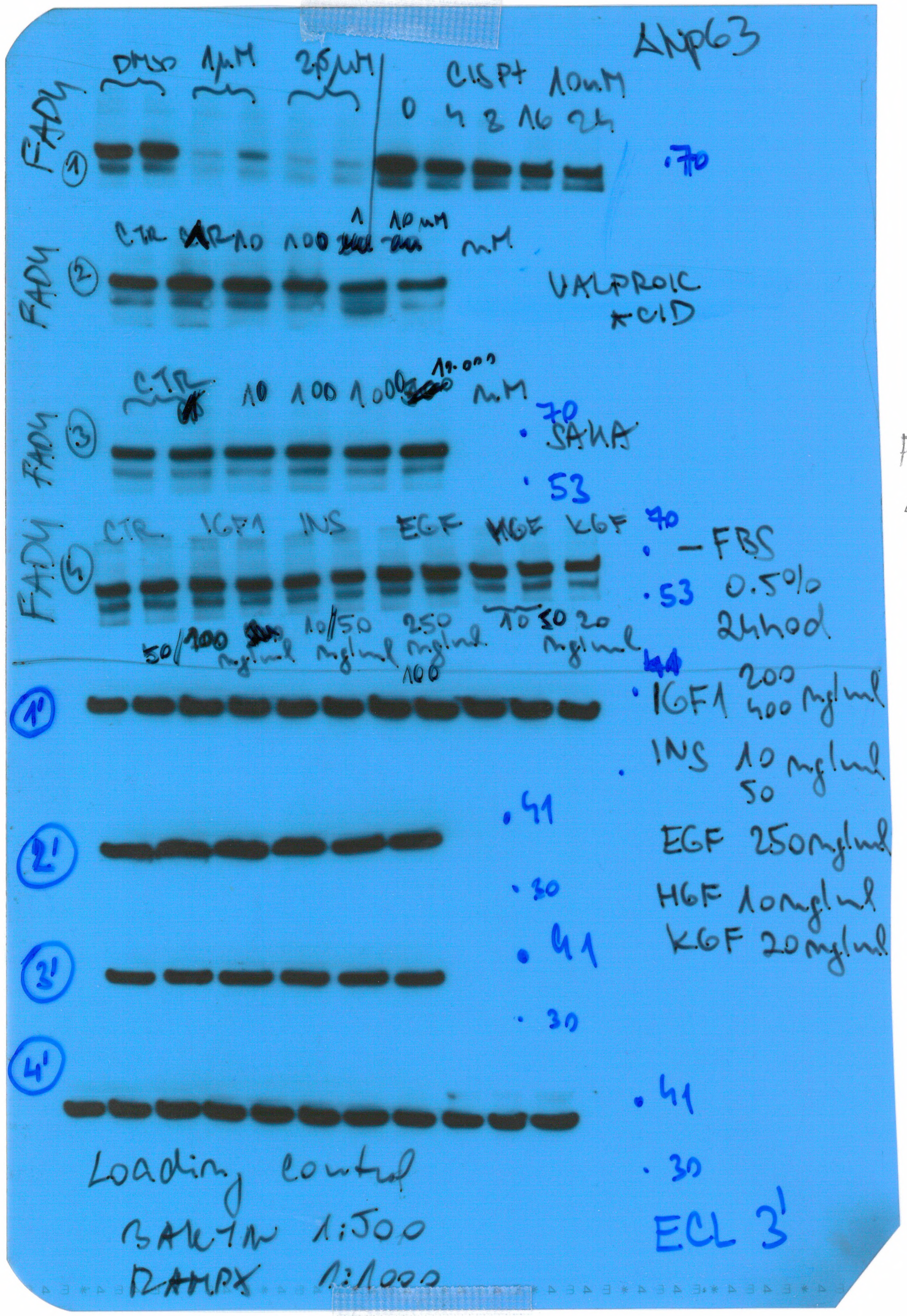

FADU, SAHA (24h) 25μg/30μl
ΔNp63 1.1 1:500

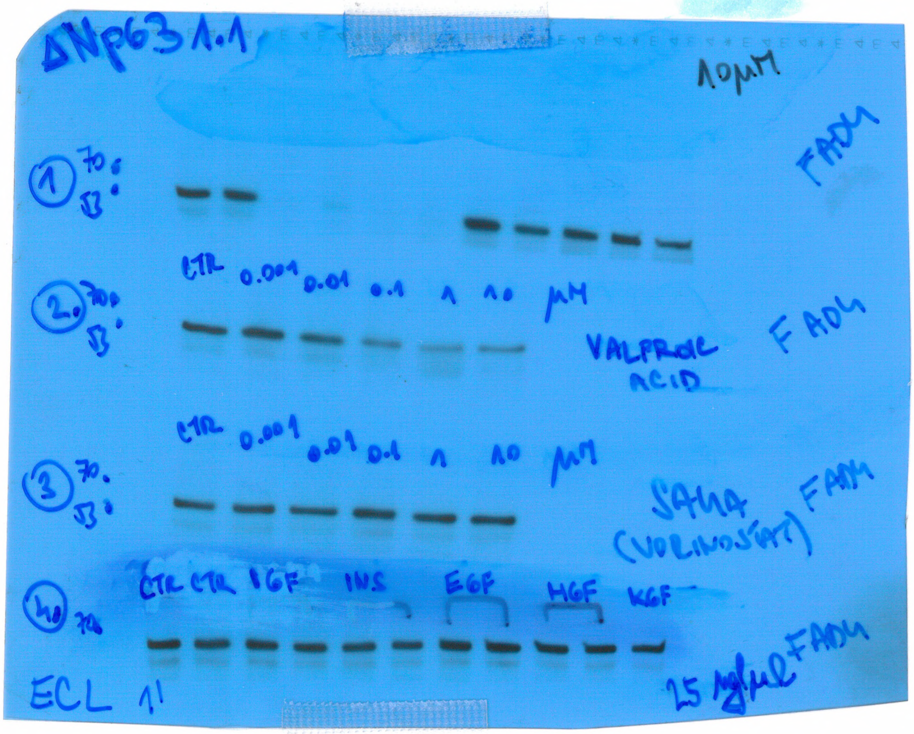

FADU, VALPROIC ACID (24h), 25μg/30μl
ΔNp63 1.1 1:500

FADU, GROWTH FACTORS (24h), 25μg/30μl
ΔNp63 1.1 1:500

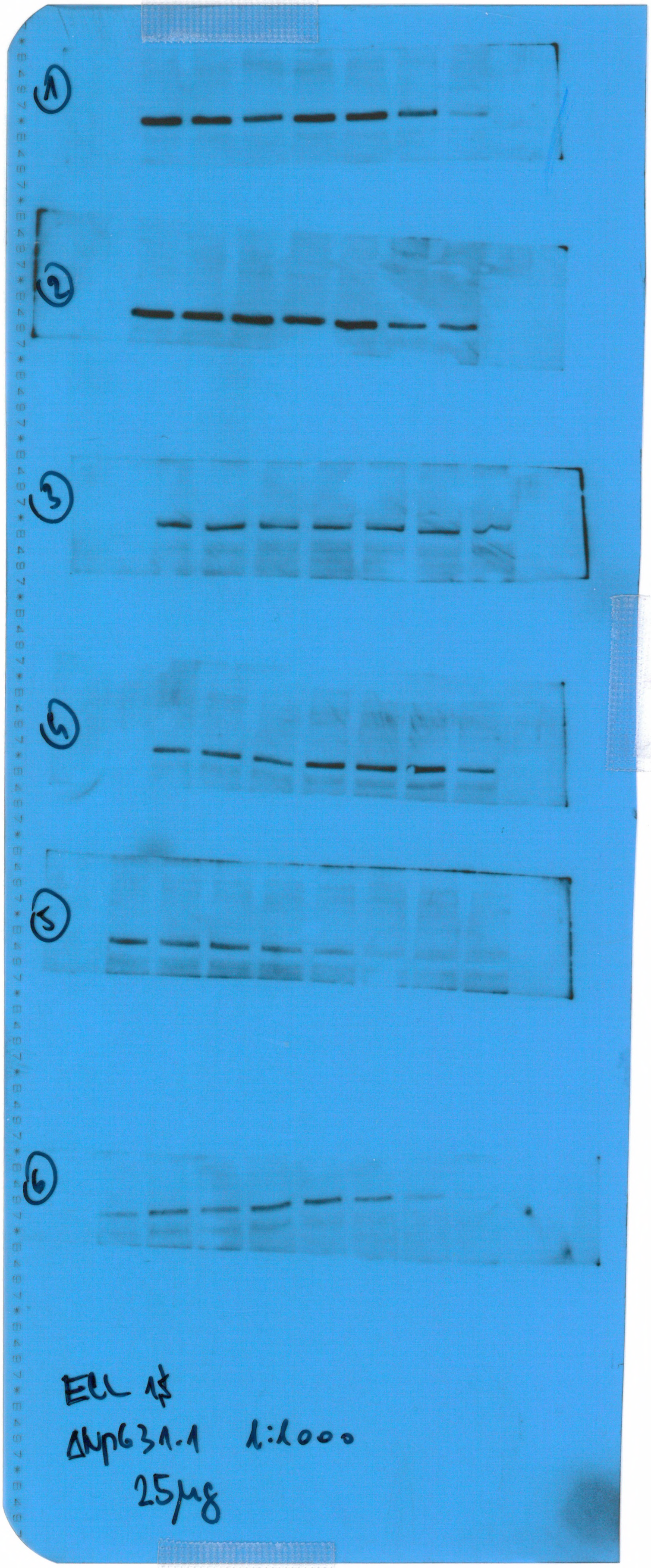

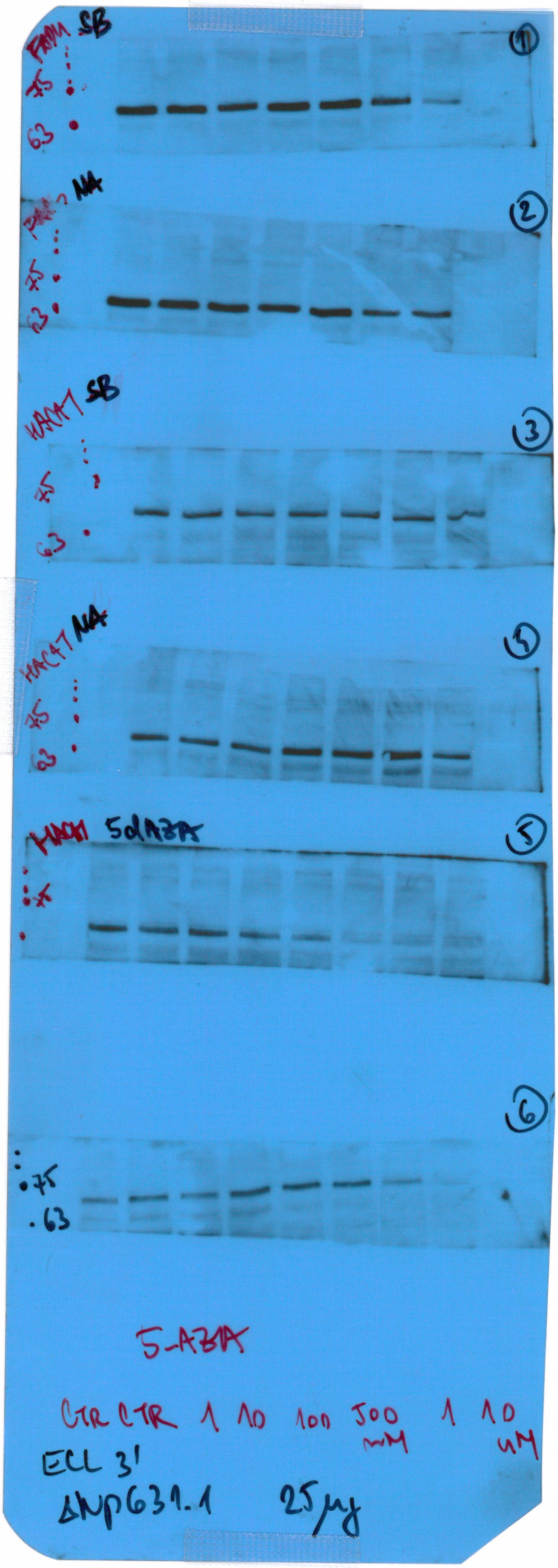

31/5/24

1 FADU SODIUMBUTYRATE 25μg
2 FADU NICOTINAMID 25μg
3 HaCaT SODIUMBUTYRATE 25μg
4 HaCaT NICOTINAMID 25μg
5 HaCaT 5-dAZA 4DAYS INCUBATION 25μg
6 FaDu 5-dAZA 4DAYS INCUBATION 25μg

} ΔNp63 1.1 1:1000

17/8/21

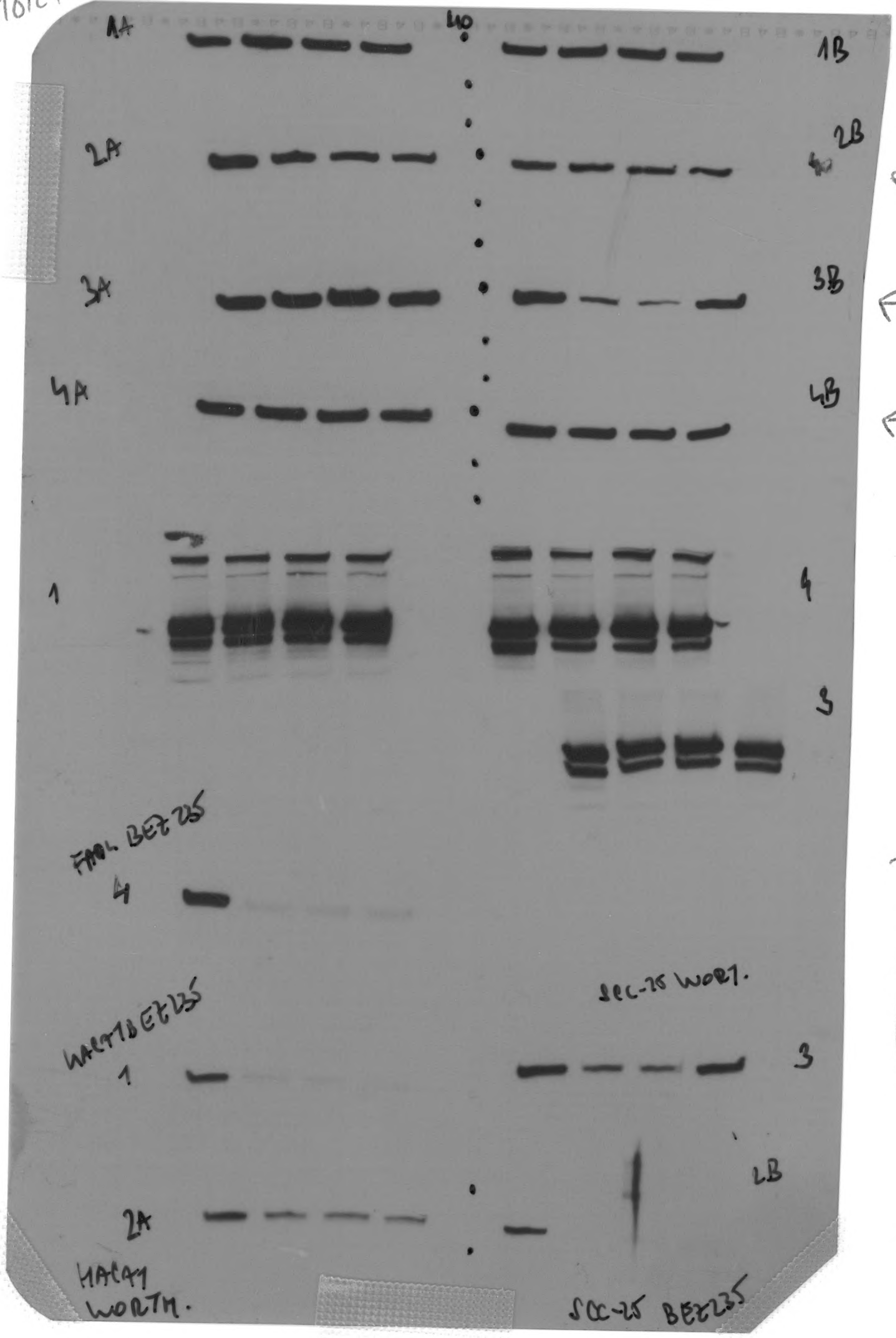

← HACAT 1A BEZ235 / 1B HACAT RAPAM.

← HACAT 2A WORT / SCC-25 2B BEZ235

← SCC-25 3A RAPAM. / SCC-25 3B WORT.

← FADU 4A BEZ235 / FADU 4B RAPAM.

β-ACTIN 1:1000 (16h)

All samples 25µg/30µl
24h treatments
(1% FBS 16h, then 24h treat.)

p-Akt 1:1000 (16h)
1 HACAT BEZ-235
2A HACAT WORTM.
2B SCC-25 BEZ235
3 SCC-25 WORTM.
4 FADU BEZ235
↓ downregulation

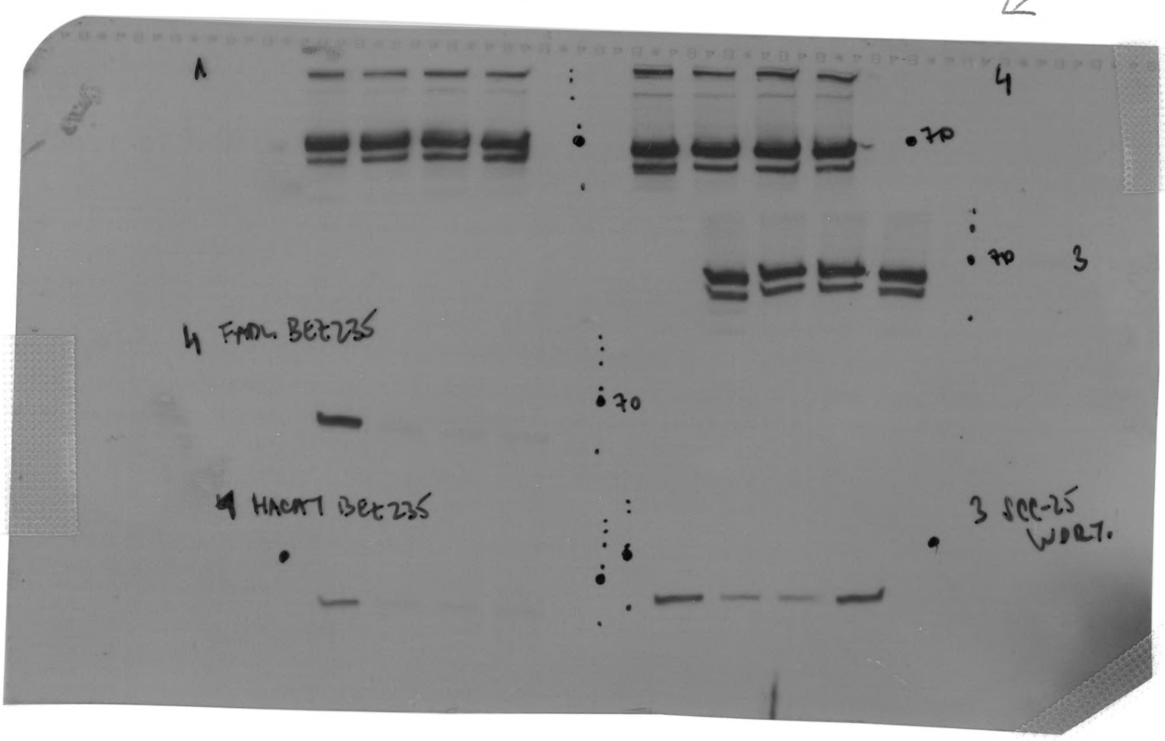

P70S6KINASE
1:1000
1 HACAT ~~BEZ235~~ RAPAMYCIN
4 FADU RAPAMYCIN
3 SCC-25 RAPAMICIN
∅ effect

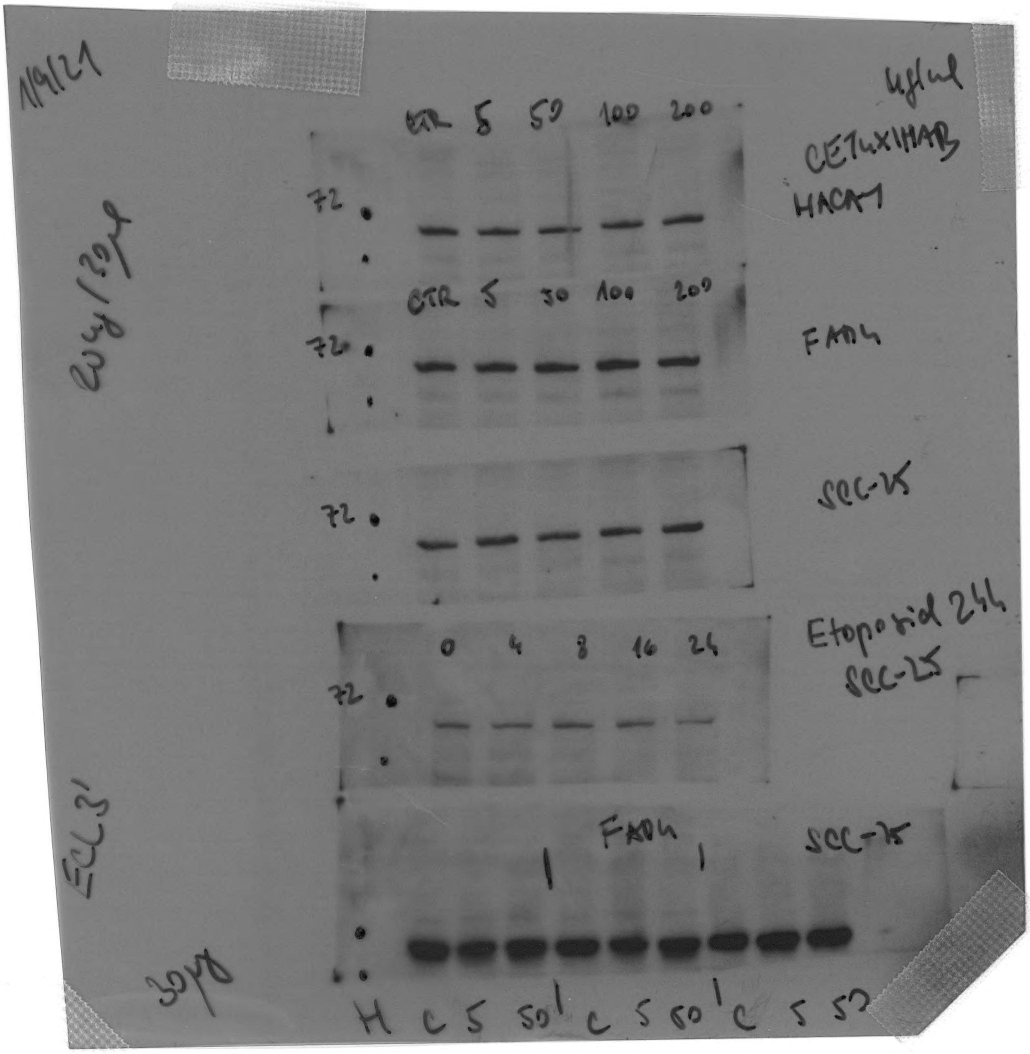
1/9/21
ug/ml
CTR 5 50 100 200
CETUXIMAB
HACAT
72
CTR 5 50 100 200
FADU
72
SCC-25
72
Etoposid 24h
SCC-25
0 4 8 16 24
72
ECL 3'
FADU
SCC-25
30ug
C 5 50 C 5 50 C 5 50

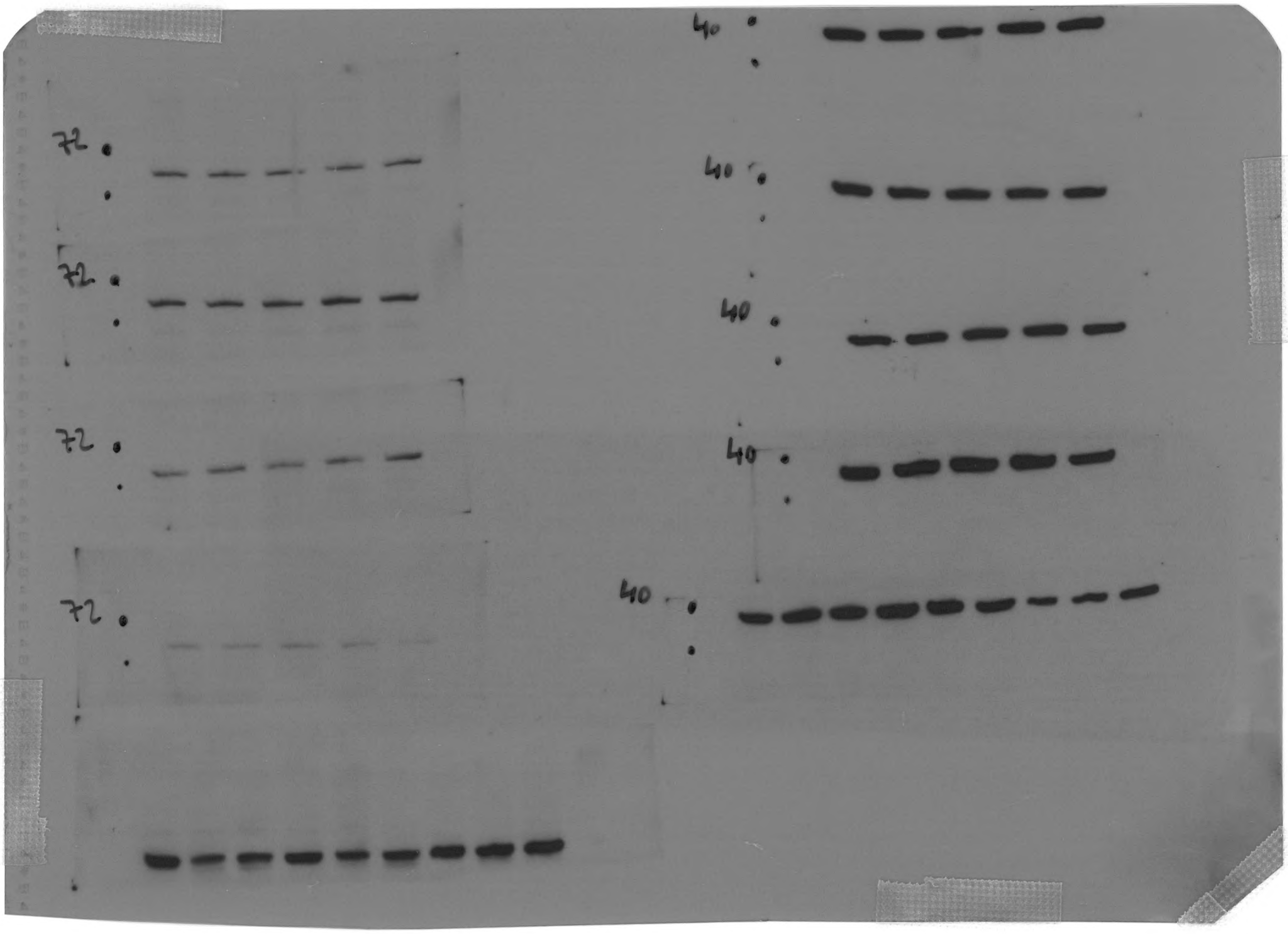
72
72
72
72
40
40
40
40
40

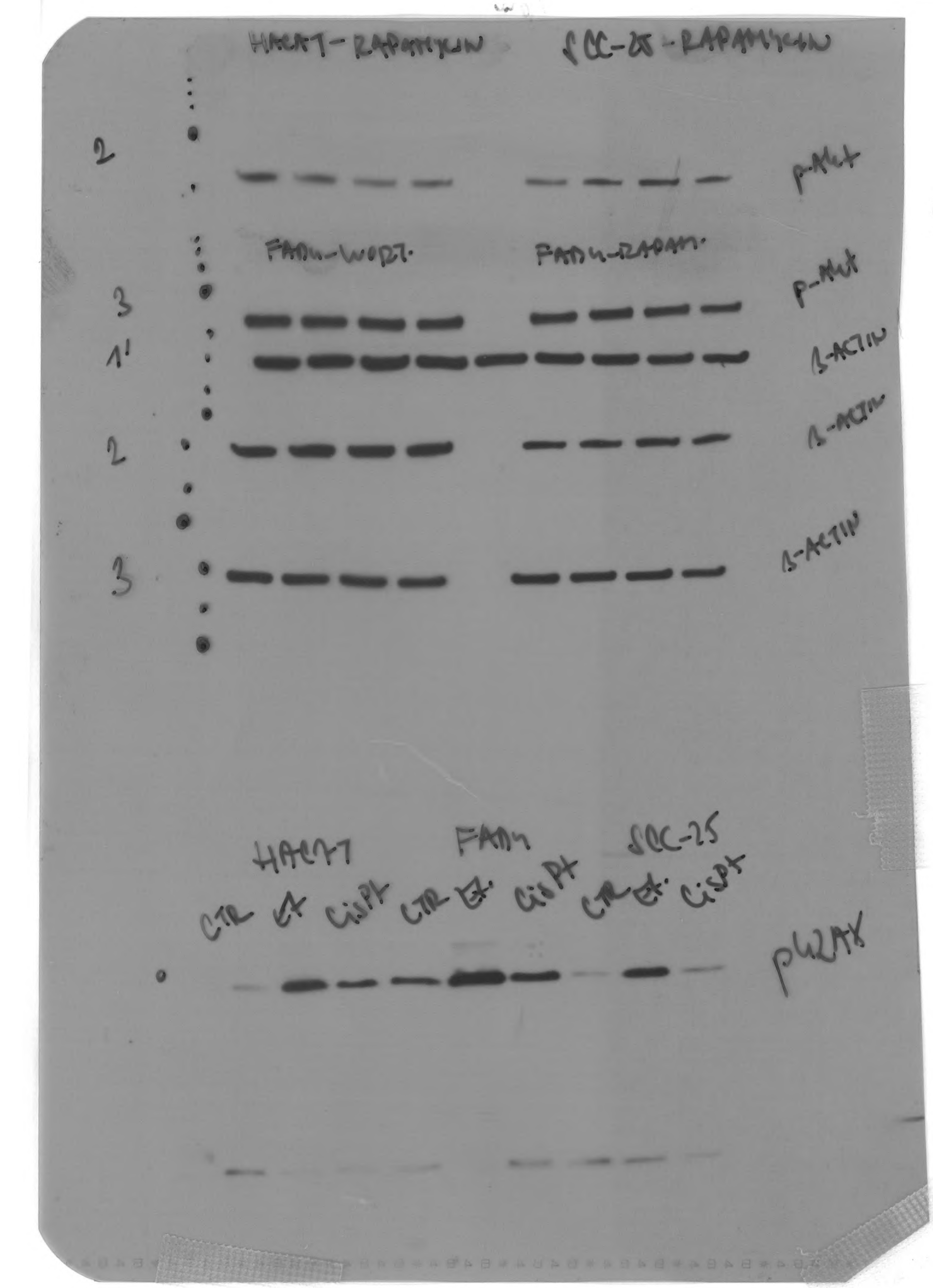

HACAT - RAPAMYCIN
~~FADU - WORTMANNIN~~
SCC-25 - RAPAMYCIN
30µg FADU - WORTMANNIN
FADU - RAPAMYCIN

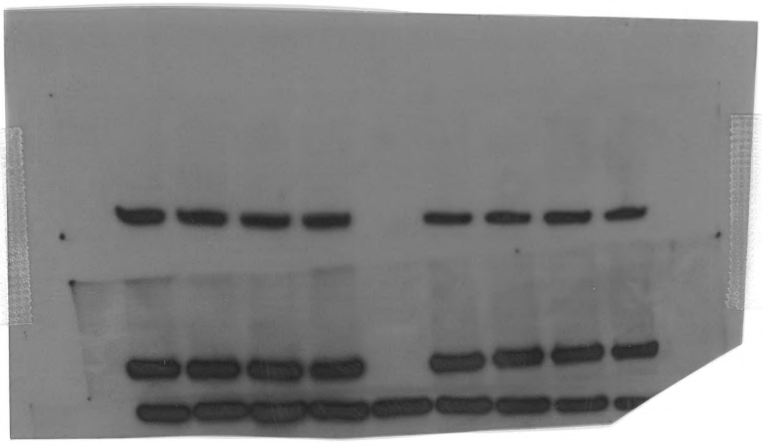

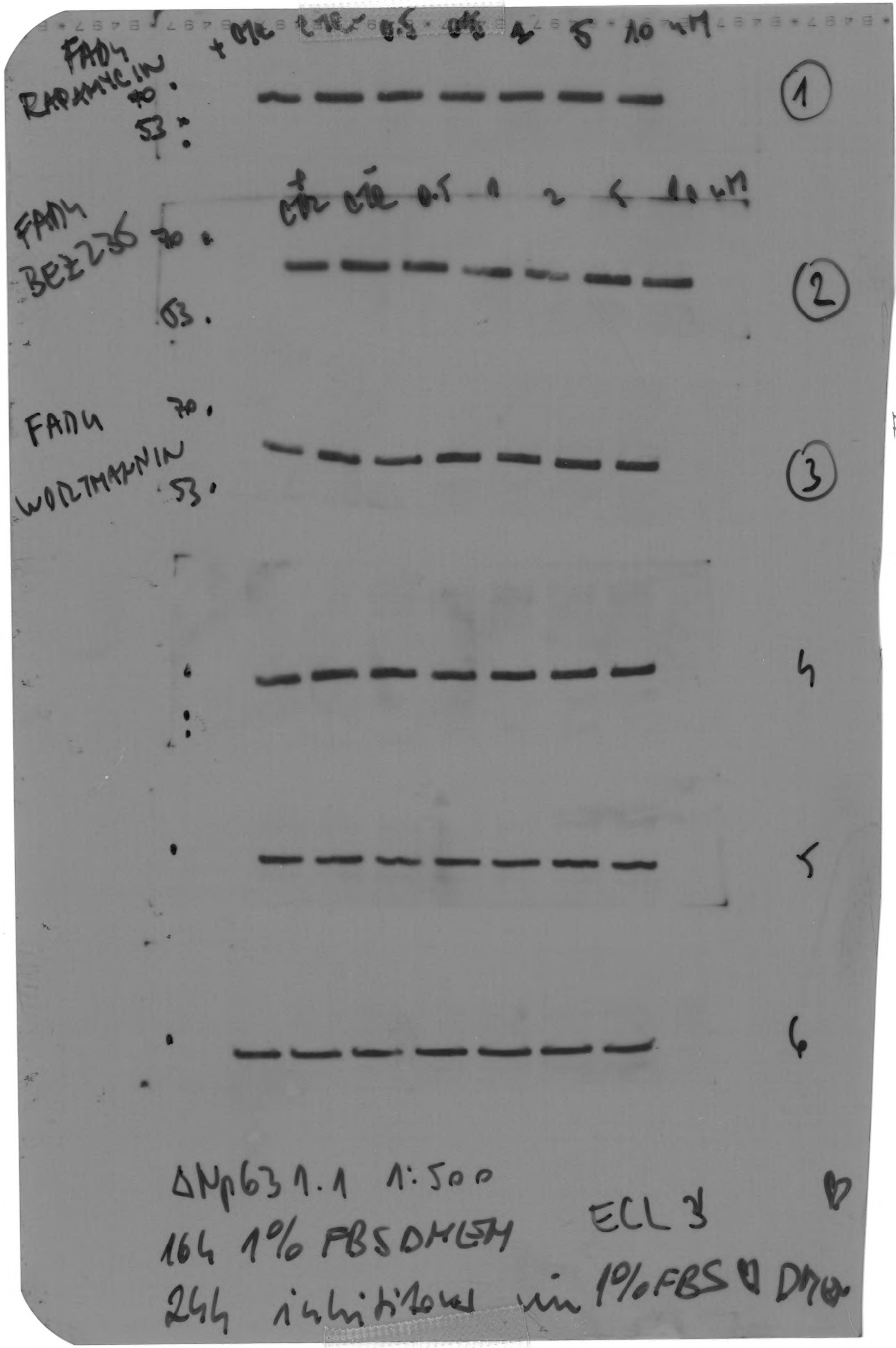

FADU, RAPAMYCIN (24h), 25µg/30µl
ΔNp63 1.1 1:500

FADU, BEZ-235 (24h), 25ug/30µl
ΔNp63 1.1 1:500

FADU, WORTMANNIN
(24h)
25ug/30µl
ΔNp63 1.1 1:500

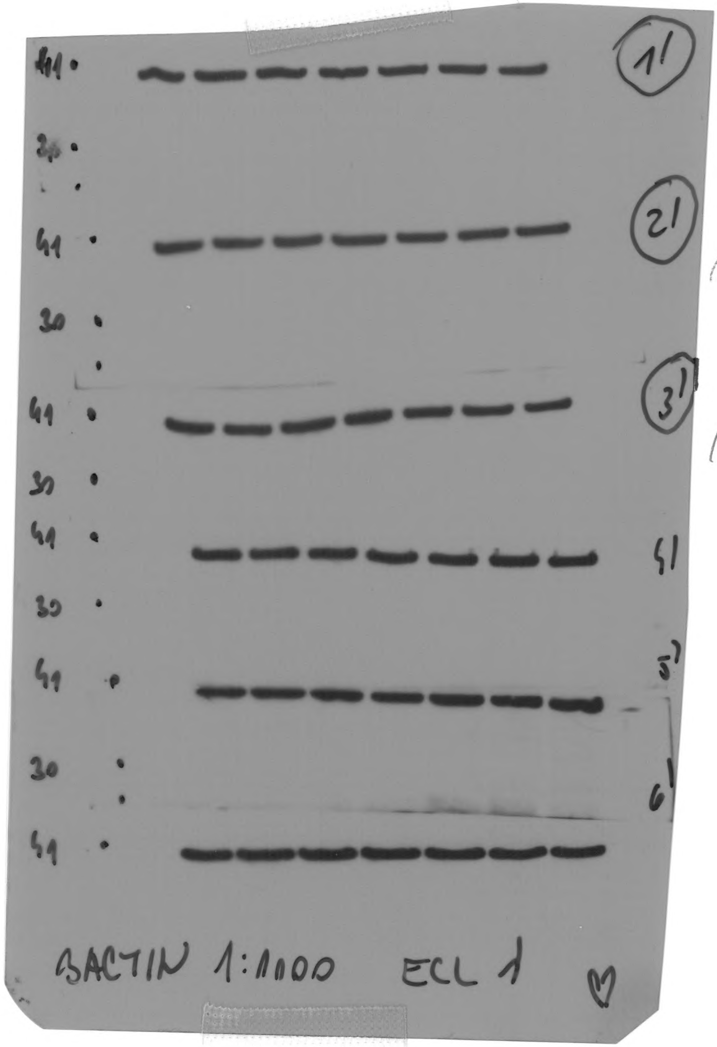

FADU, RAPAMYCIN
BACTIN 1:1000

FADU, BEZ-235
BACTIN 1:1000

FADU, WORTMANNIN
BACTIN 1:1000

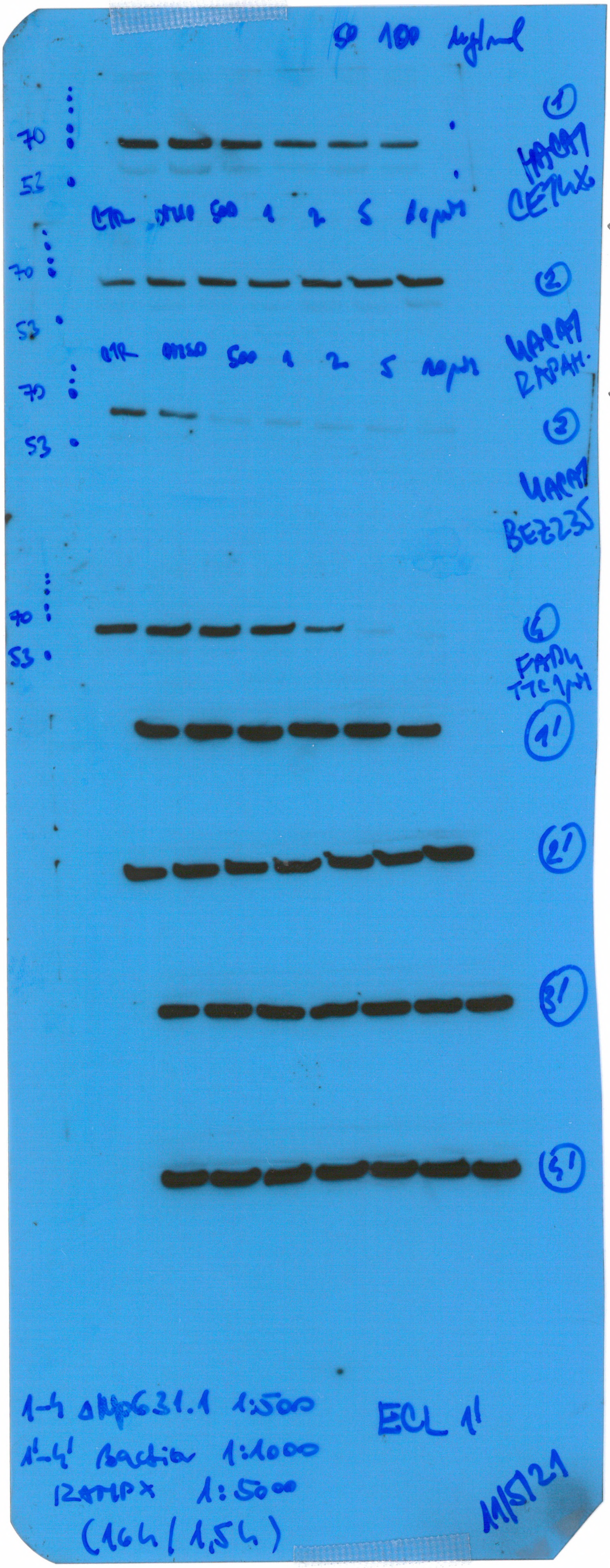

(2) HACAT, RAPAMYCIN (24h), 25µg/30µl
ΔNp63 1.1 1:500

(3) HACAT, BEZ-235 (24h), 25µg/30µl
ΔNp63 1.1 1:500

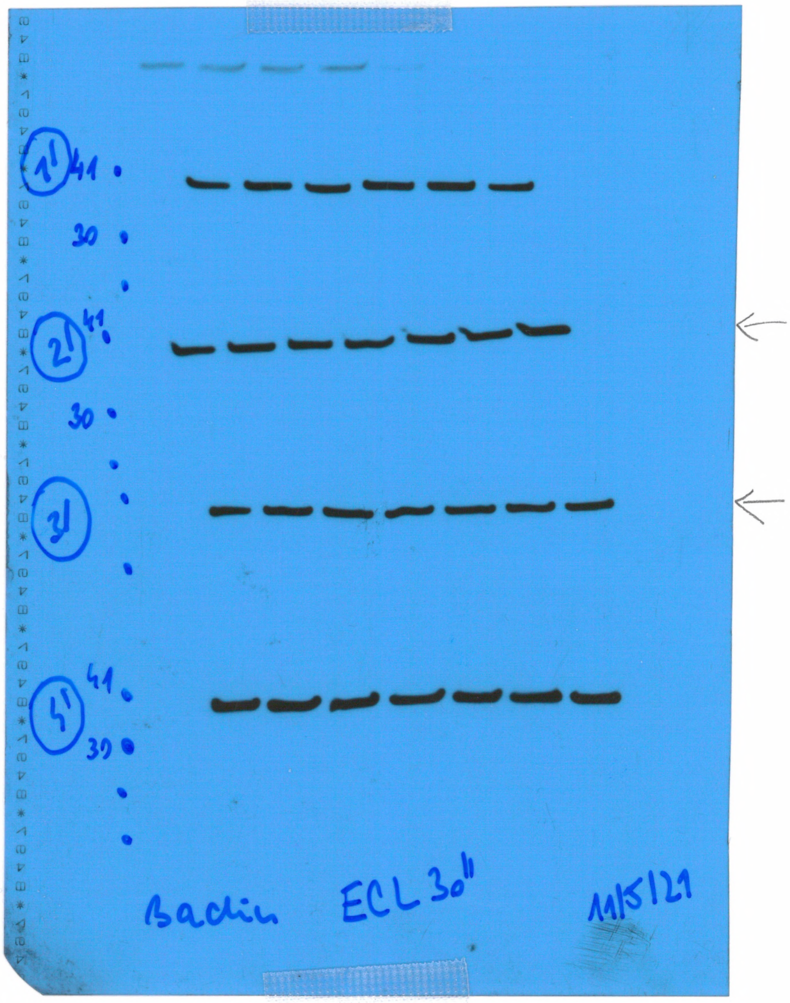

(2') HACAT, RAPAMYCIN BACTIN 1:1000

(3') HACAT, BEZ-235 BACTIN 1:1000

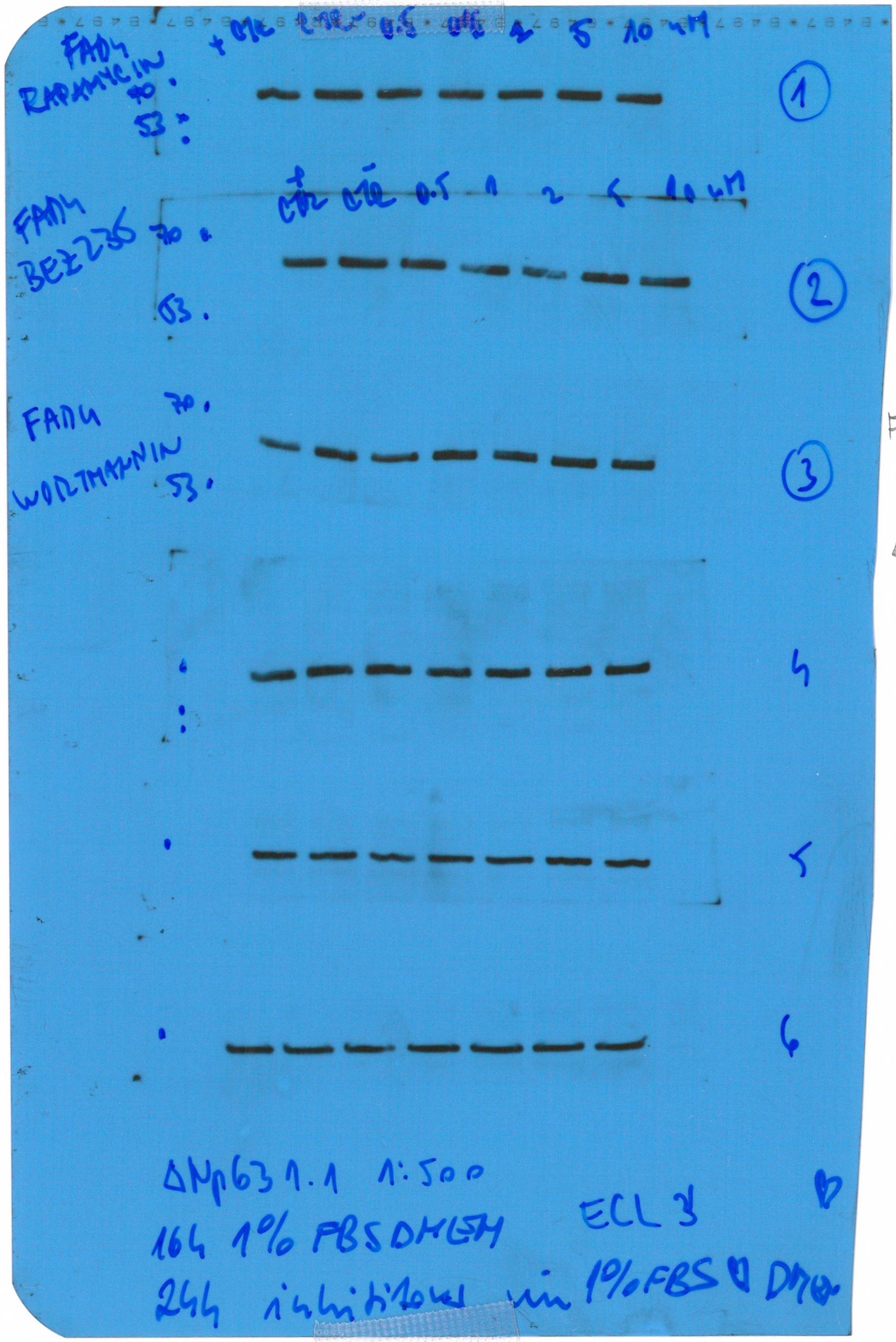

FADU, RAPAMYCIN (24h), 25μg/30μl
ΔNp63 1.1 1:500

FADU, BEZ-235 (24h), 25μg/30μl
ΔNp63 1.1 1:500

FADU, WORTMANNIN (24h)
25μg/30μl
ΔNp63 1.1 1:500

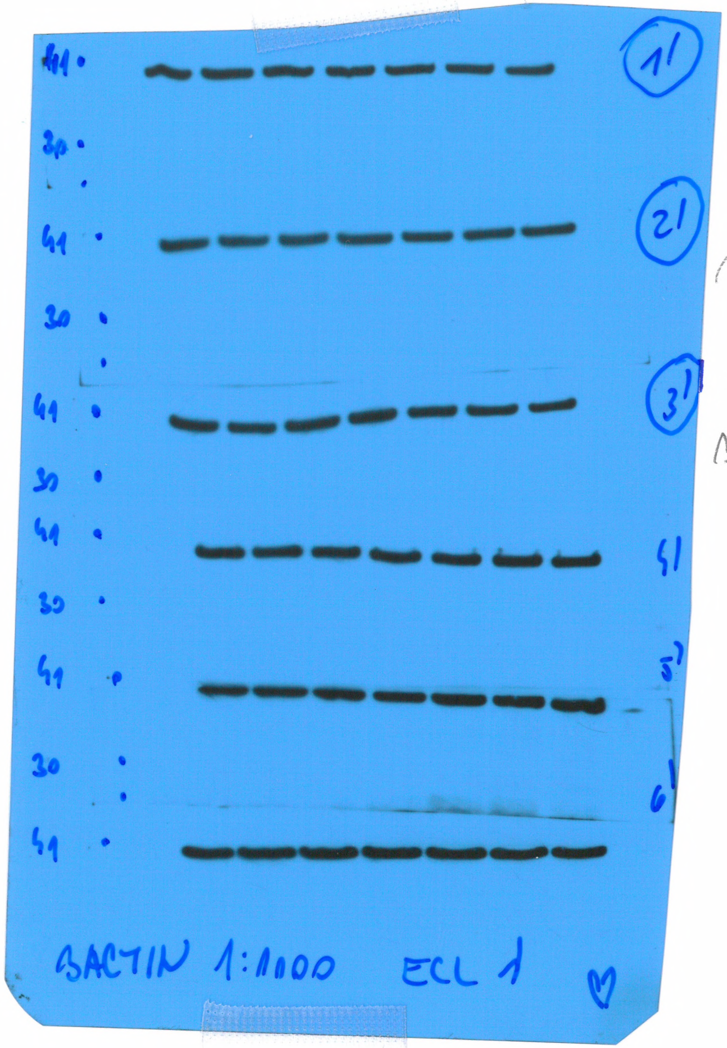

FADU, RAPAMYCIN
BACTIN 1:1000

FADU, BEZ-235
BACTIN 1:1000

FADU, WORTMANNIN
BACTIN 1:1000

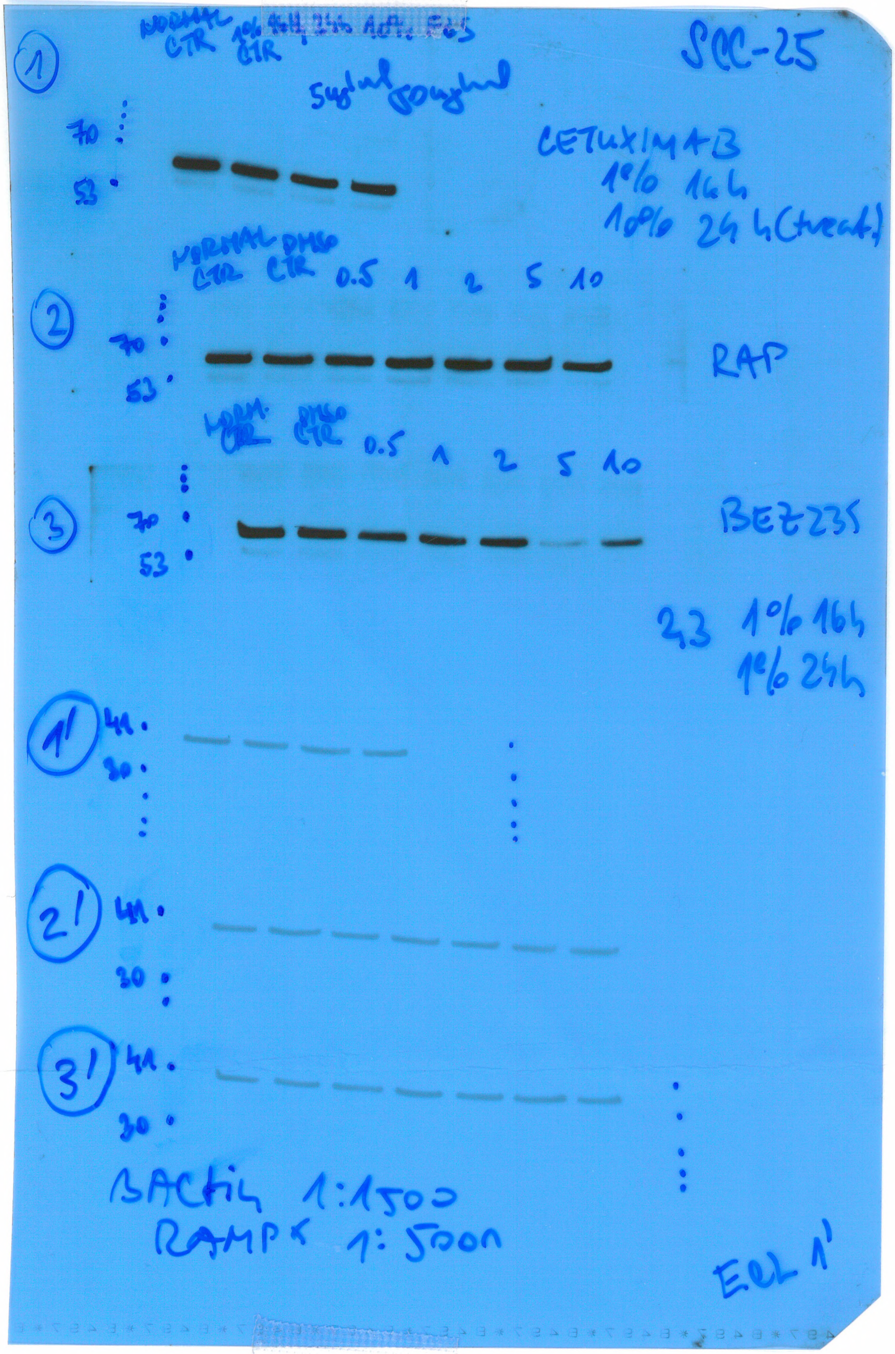

SCC-25 CETUXIMAB (24h) 30μg/μl
ΔNp63 1.1 1:500

SCC-25 RAPAMYCIN (24h) 30μg/μl
ΔNp63 1.1 1:500

SCC-25 BEZ-25 (24h) 30μg/30μl
ΔNp63 1.1 1:500

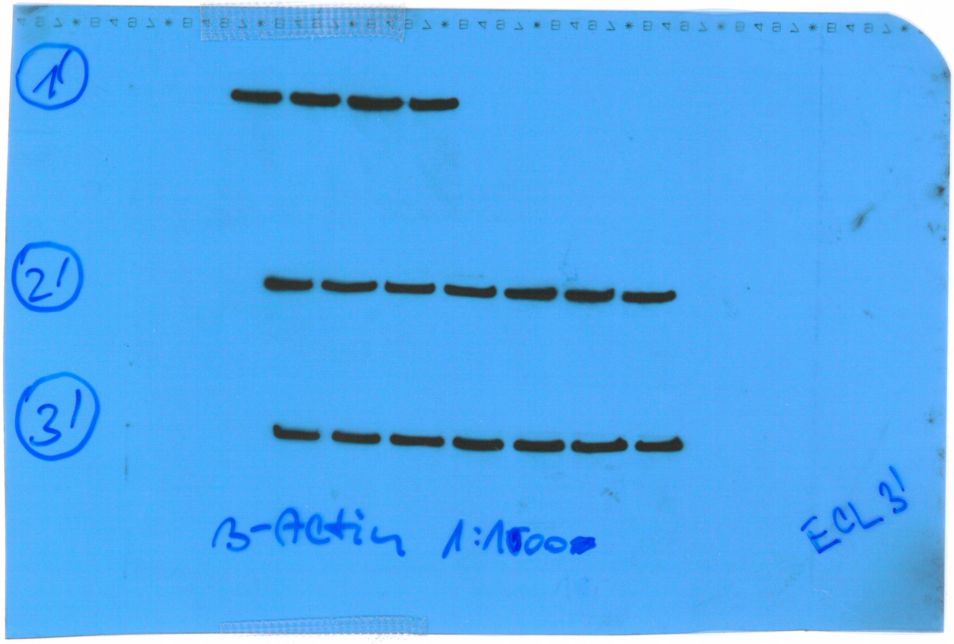

SCC-25 CETUXIMAB
B-ACTIN 1:1000

SCC-25 RAPAMYCIN
B-ACTIN 1:1000

SCC-25 BEZ-25
B-ACTIN 1:1000

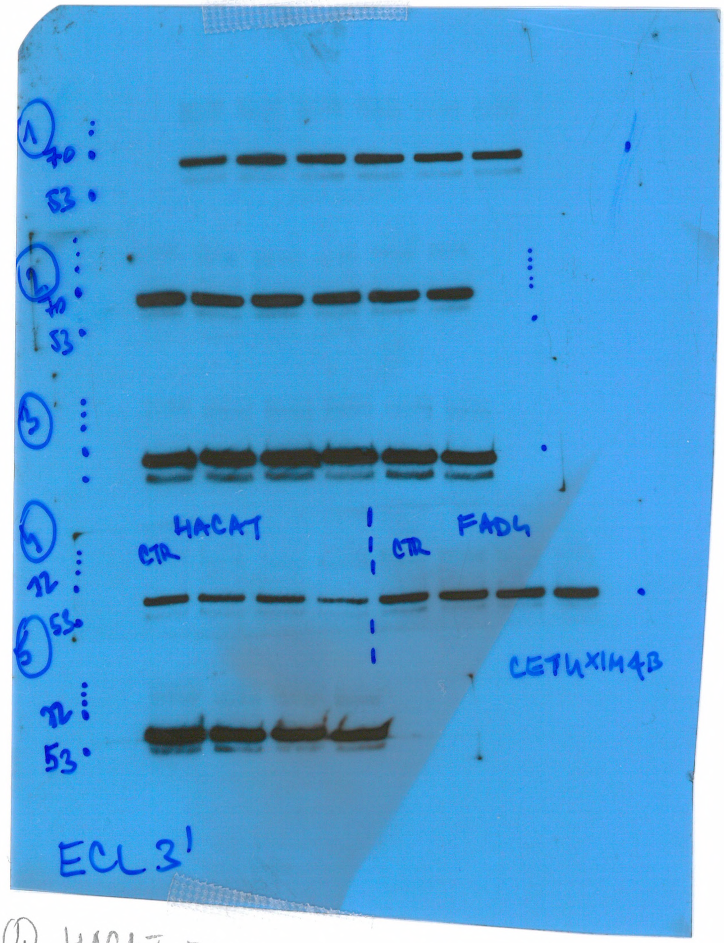

(4) HACAT, FADU CETUXIMAB 25µg/30µl
ΔNp63 1.1 1:500

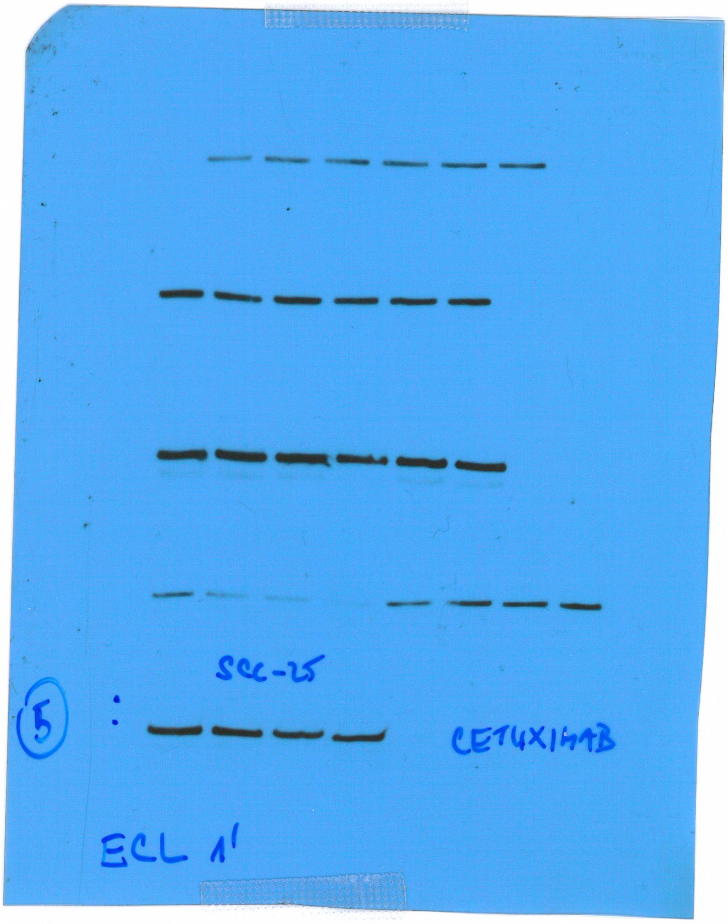

(5) SCC-25, CETUXIMAB 25µg/30µl
ΔNp63 1.1 1:500

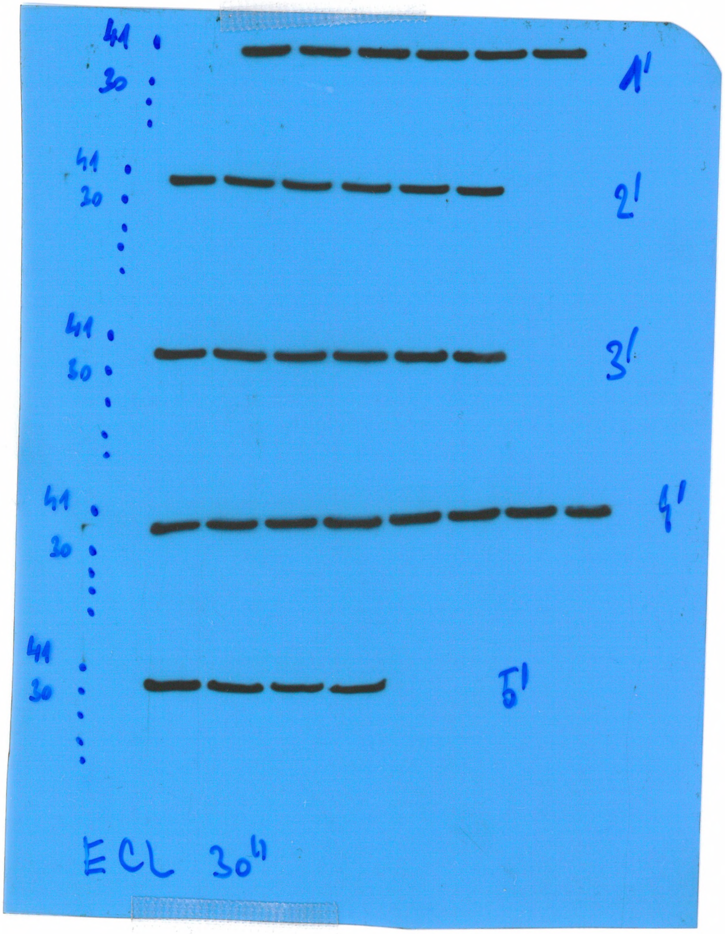

4',5' HACAT, FADU, SCC-25
β-ACTIN 1:1000

3/5/21

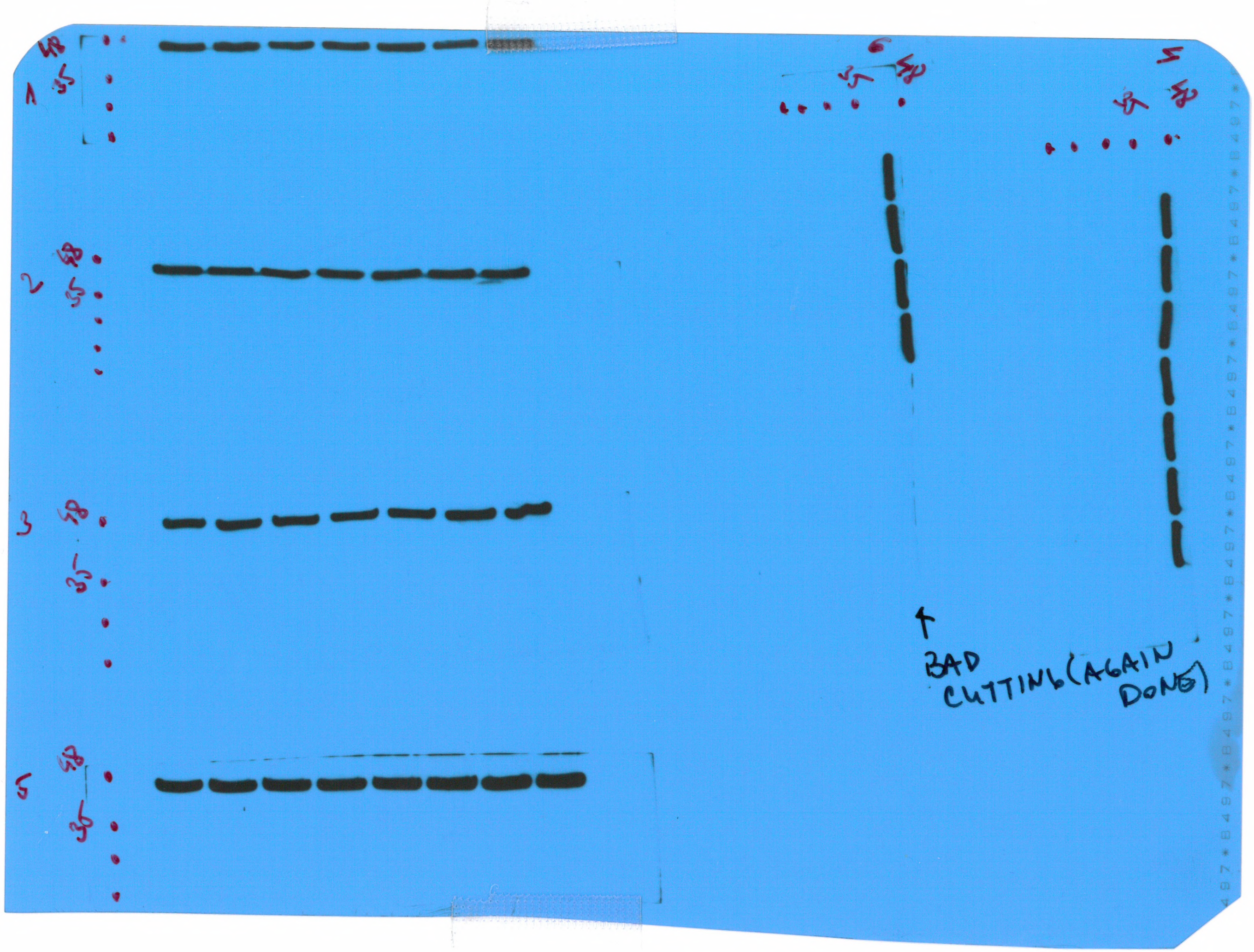

1 FADU SODIUM BUTYRATE
2 FADU NICOTINAMIDE
3 HaCaT SODIUM BUTYRATE
4 HaCaT NICOTINAMIDE
5 HaCaT 5-dAZA
6 FADU 5-dAZA

β-actin - all images
1:1000

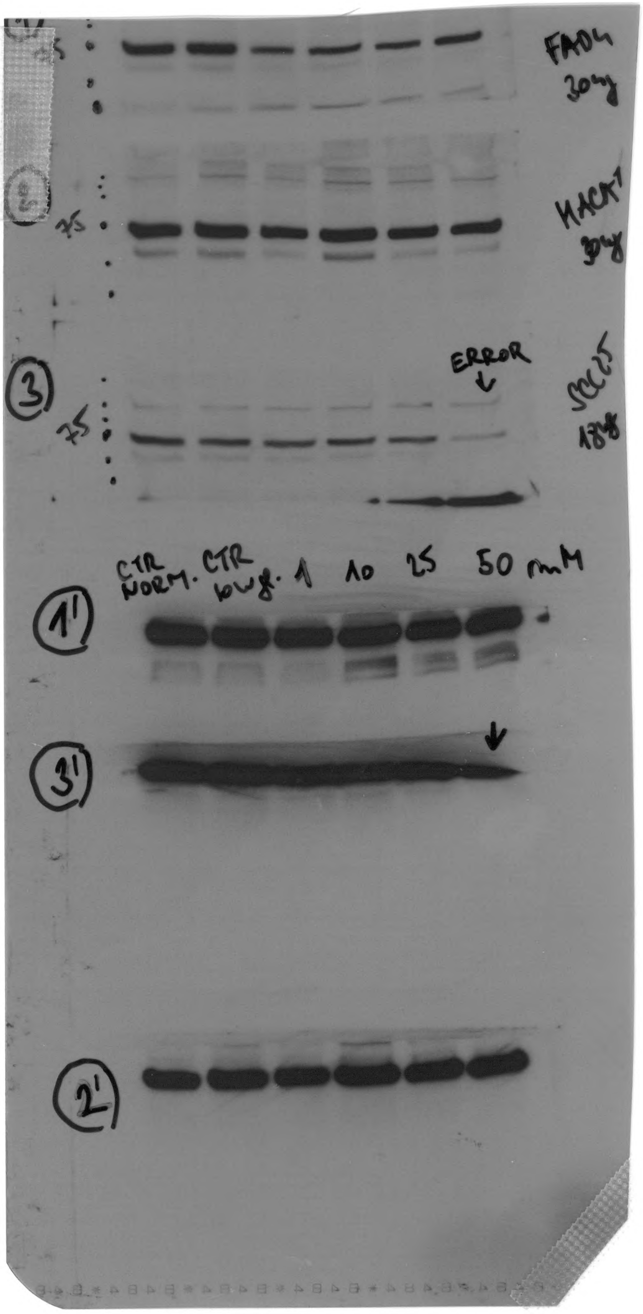

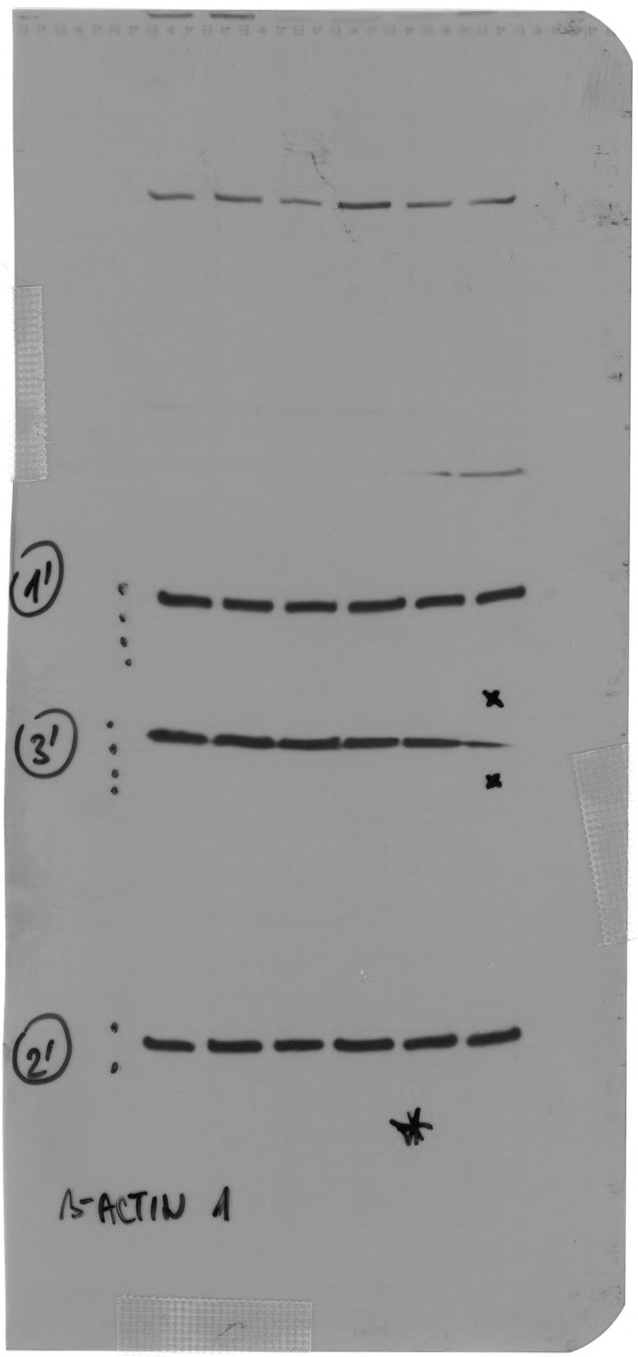

HACAT, FADU, SCC-25   Metformin 1-50 mM
1% SERUM FBS / low glucose medium

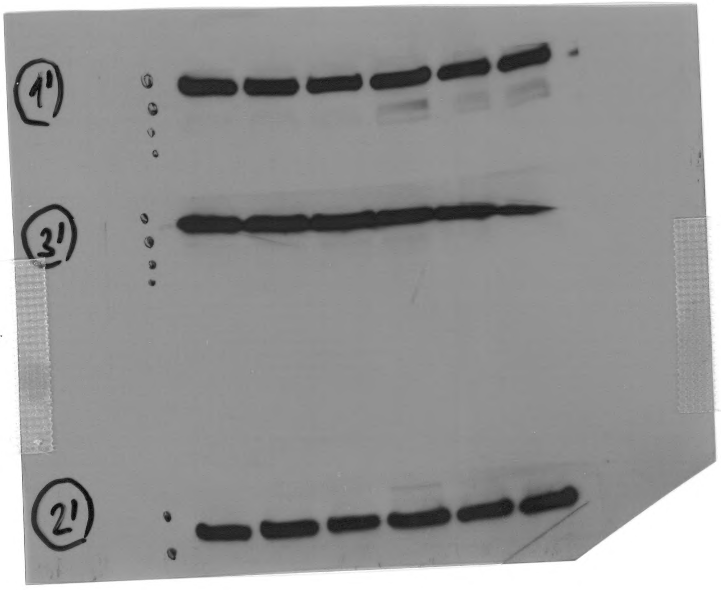

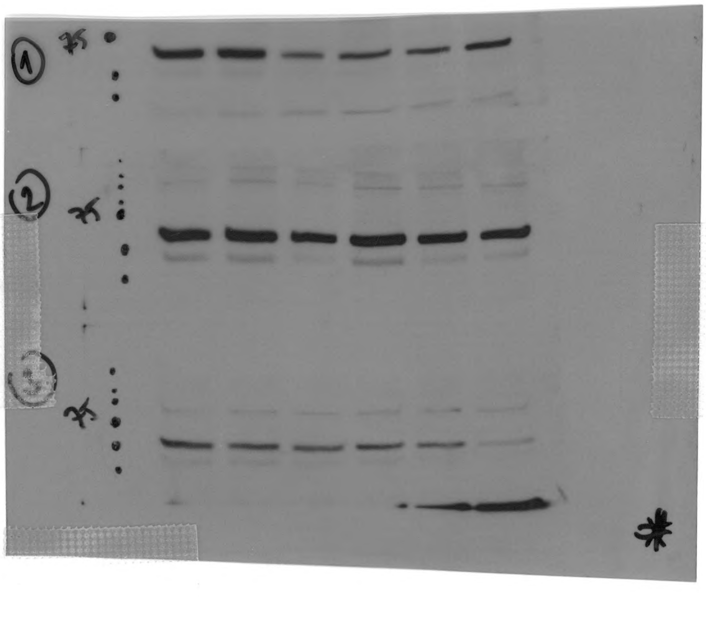

✓ 11/3/21

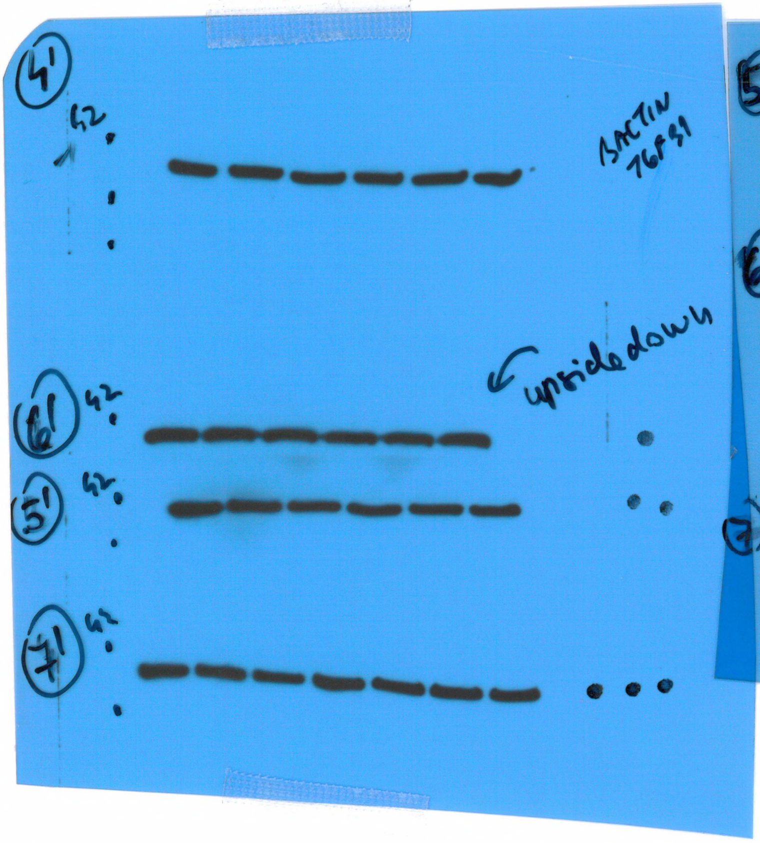

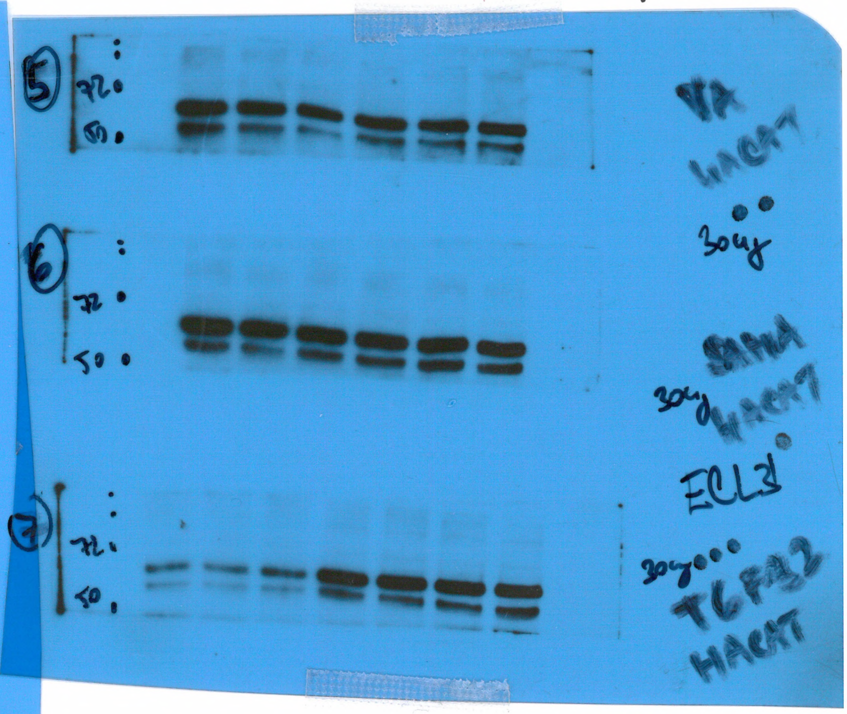

(4') HACAT, TGFB1, B-ACTIN

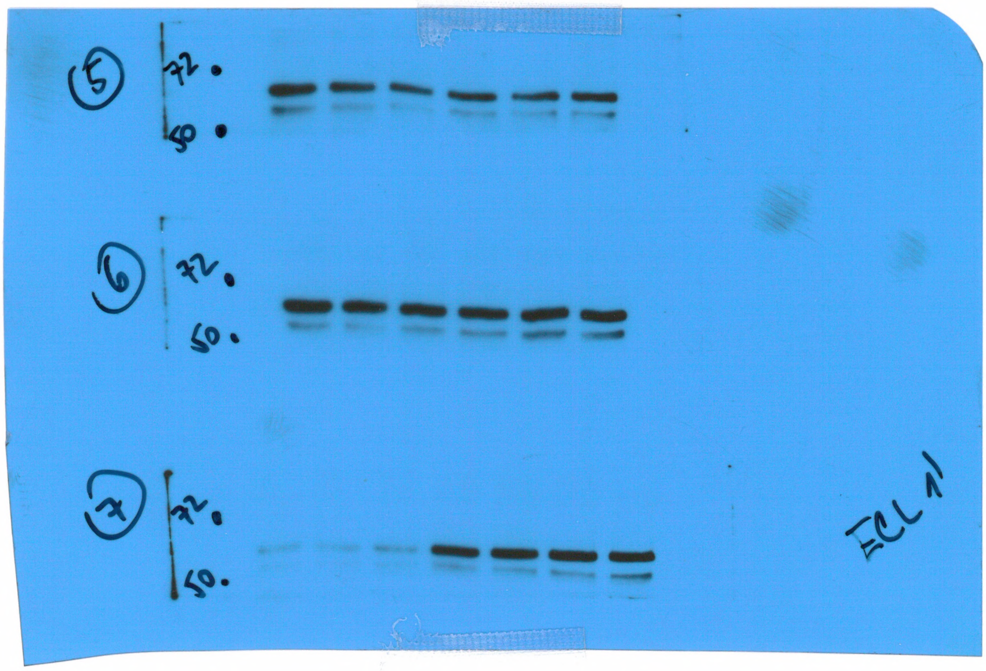

(5) HACAT, VALPROIC ACID, ΔNp63 1.1 1:1000

(6) HACAT, SAHA, ΔNp63 1.1 1:1000

(7) HACAT, TGFB2, ΔNp63 1.1 1:1000

(5') HACAT, VA, B-ACTIN 1:1000

(6') HACAT, SAHA, B-ACTIN 1:1000

(7') HACAT, TGFB2, B-ACTIN 1:1000

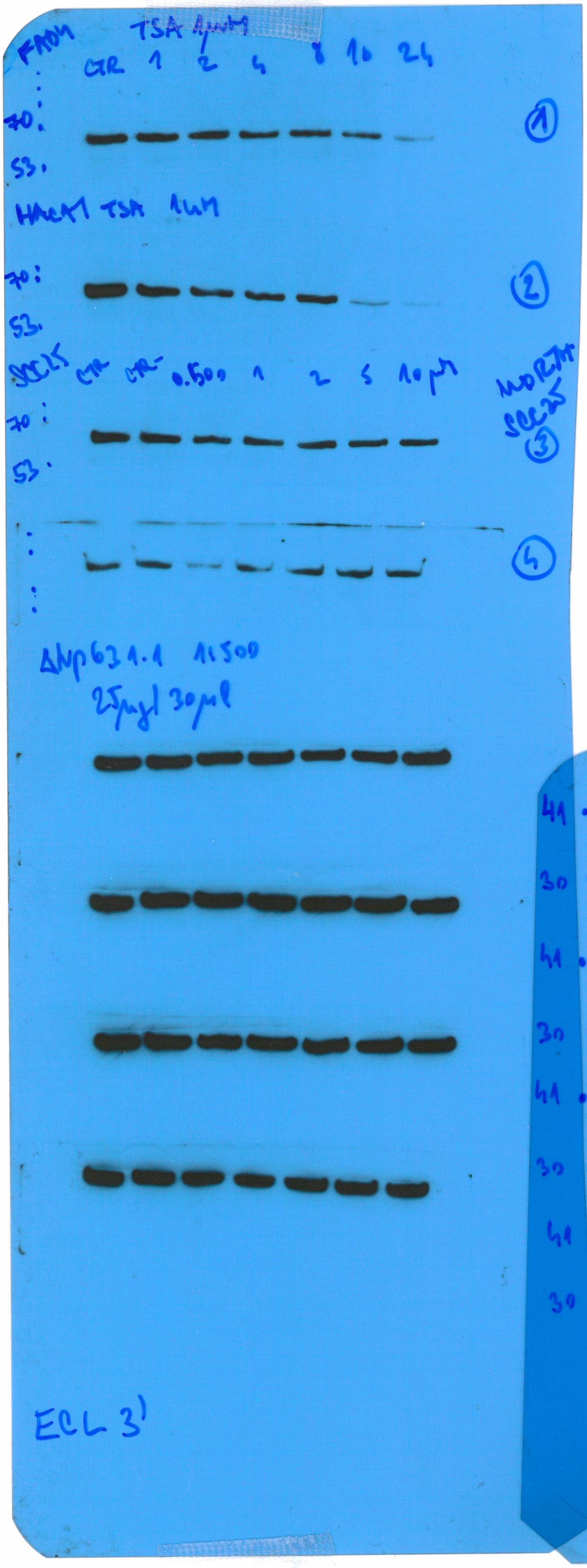

FADU, TRICHOSTATIN (1-24h), 25µg/30µl
ΔNp63 1.1 1:500

HACAT, TRICHOSTATIN (1-24h), 25µg/30µl
ΔNp63 1.1 1:500

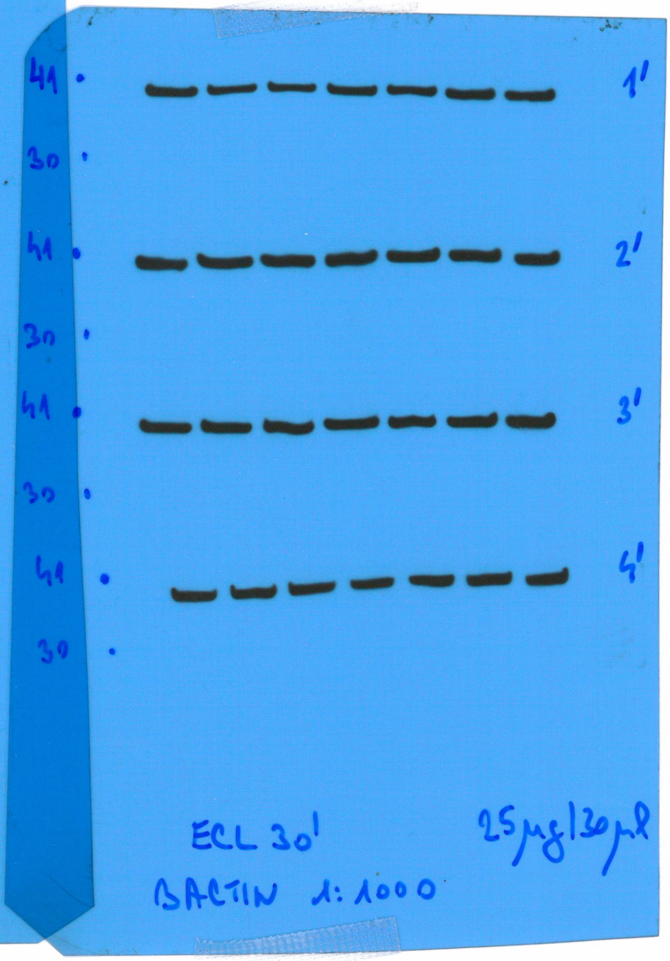

FADU, TRICHOSTATIN
BACTIN 1:1000

HACAT, TRICHOSTATIN
BACTIN 1:1000

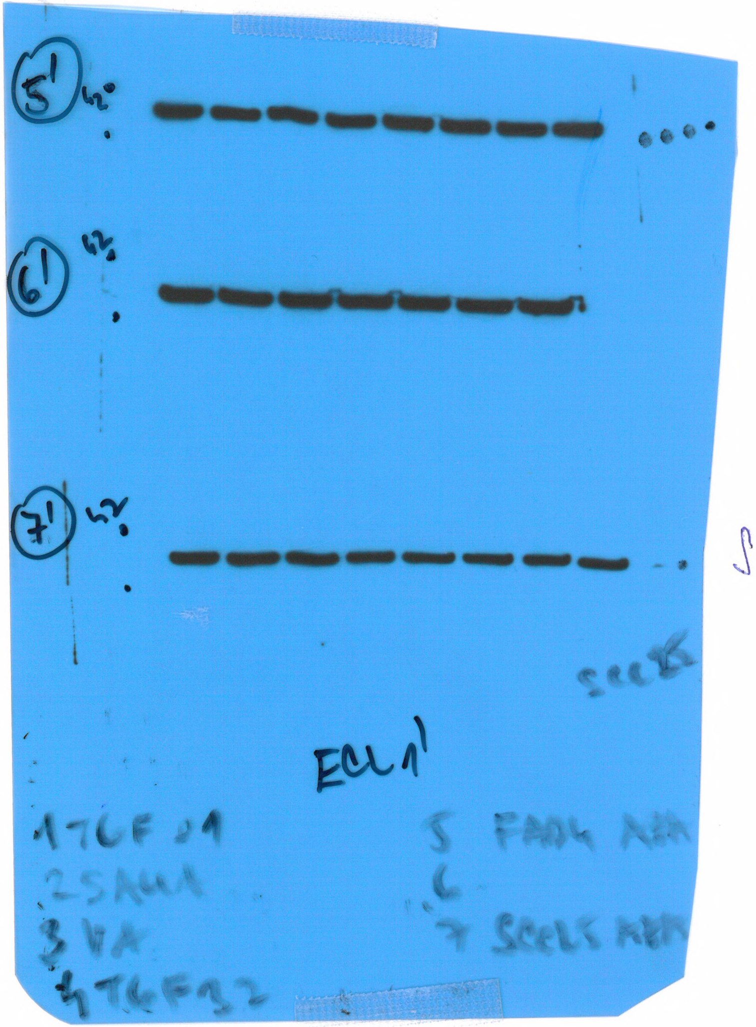

FADU, 5-dAZA, B-ACTIN 1:1000

SCC-25, 5-dAZA, B-ACTIN 1:1000

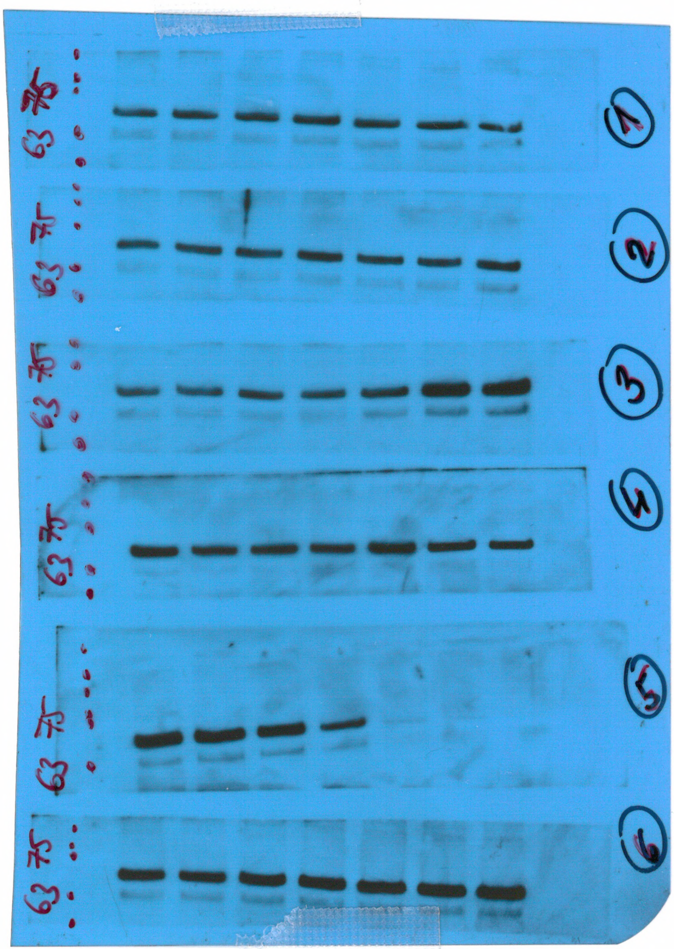

HACAT, LOVASTATIN (24h) ΔNp63 1.1 1:1000

FADU, LOVASTATIN (24h) ΔNp63 1.1 1:1000

SCC-25, TRICHOSTATIN ΔNp63 1.1 1:1000

SCC-25, LOVASTATIN (24h) ΔNp63 1.1 1:1000

ECL 3'

ECL 6'

HACAT, FADU

LOVASTATIN, INHIBITORS (BEZ235, TSA, SB, CAL101) 15/10/2020

24h.

30ug

ΔNp63 1.1 1:1000

β ACTIN 1:1000

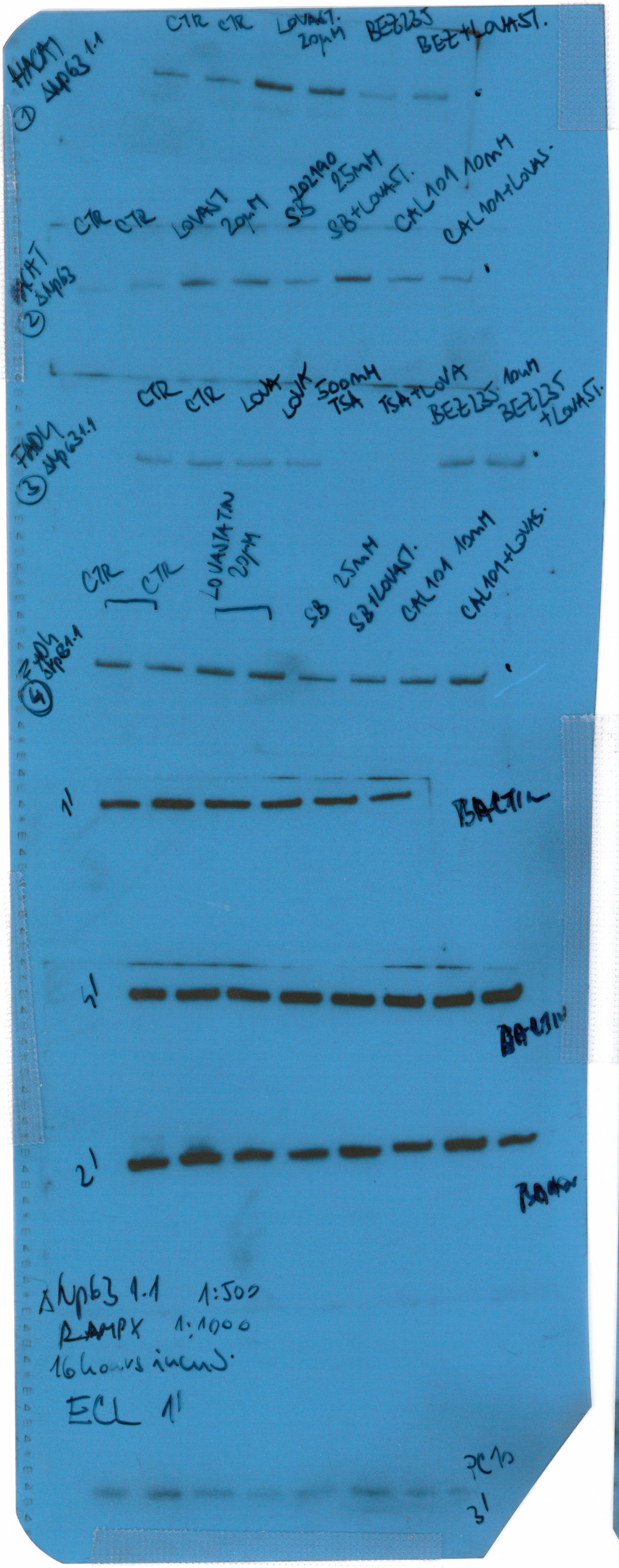

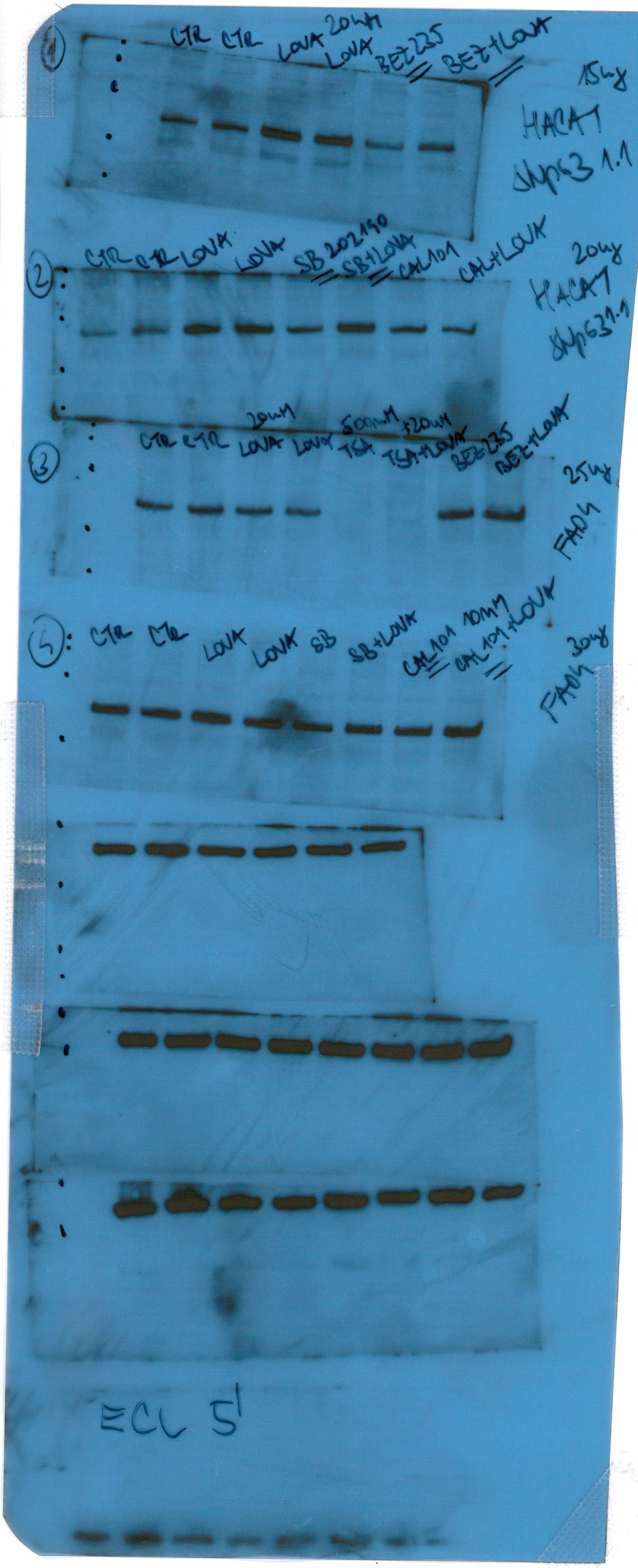

9/5/21

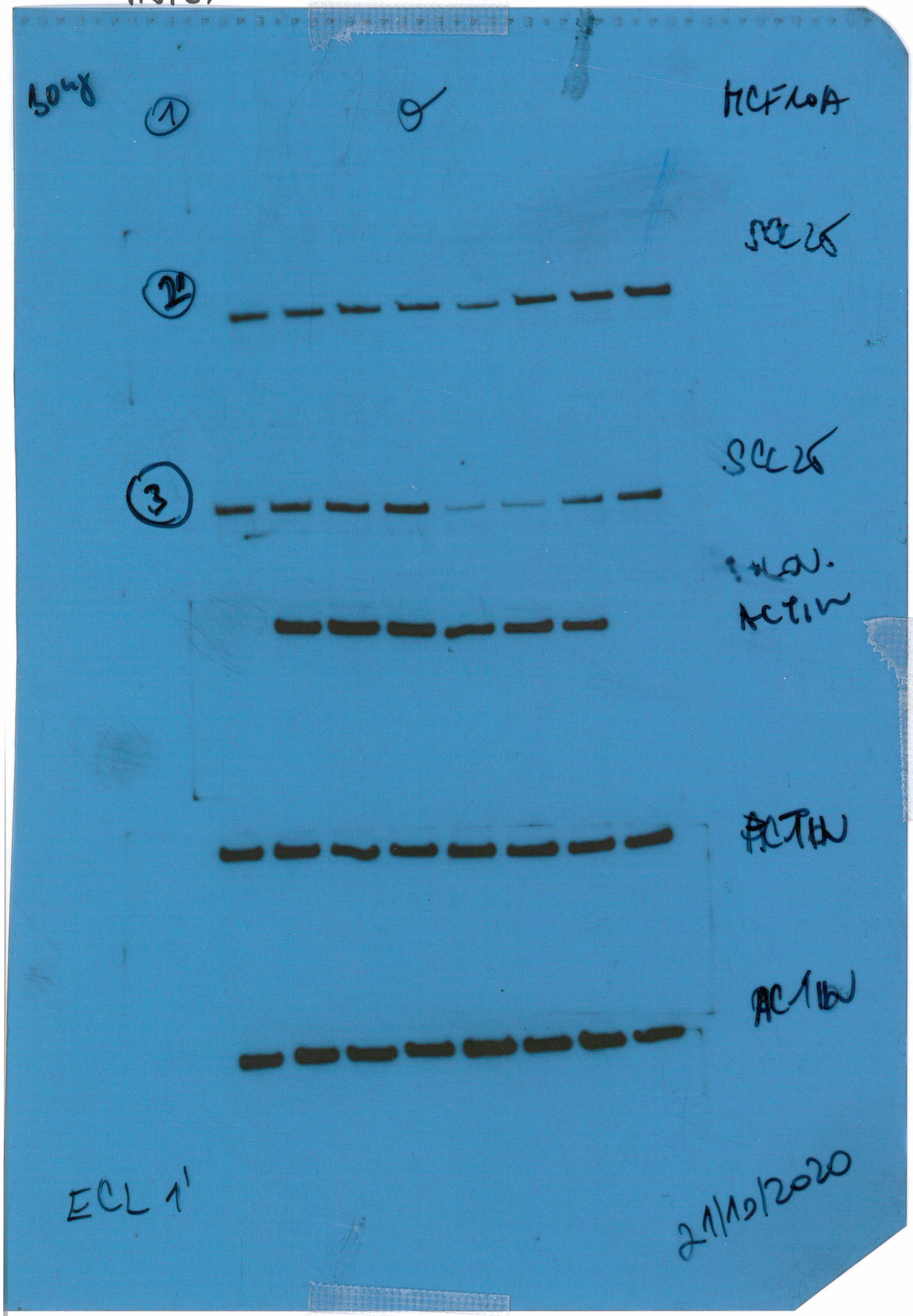

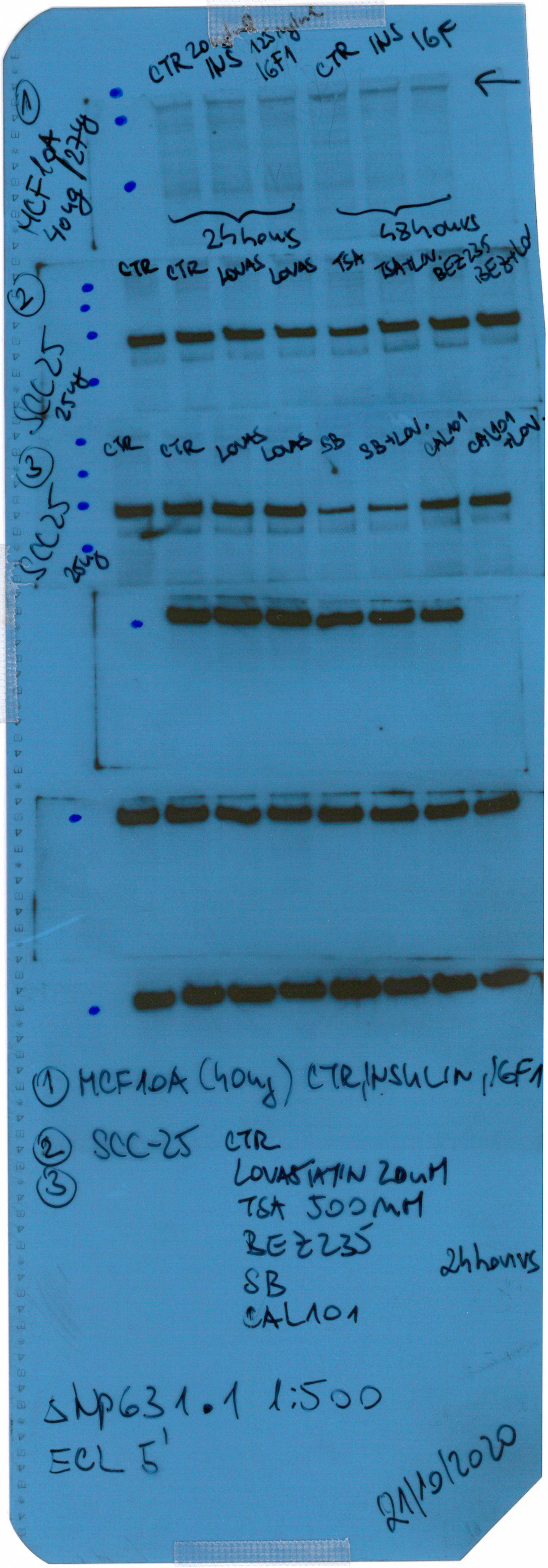

2,3)
SCC-25
LOVASTATIN ± INHIBITORS
(TSA, BEZ235, SB, CAL101)
ΔNp63 1:500

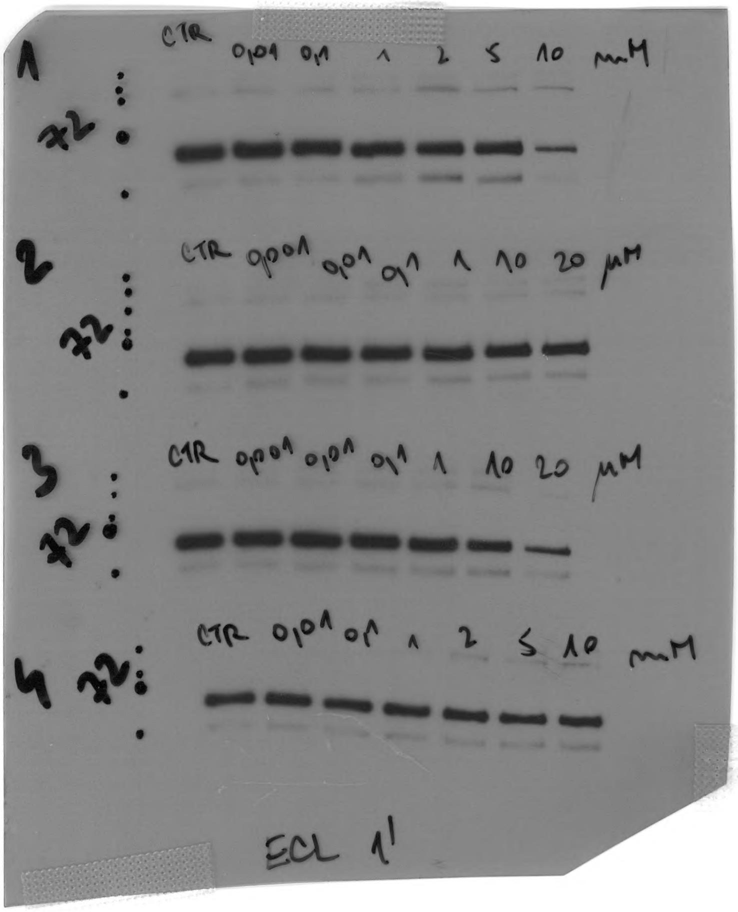

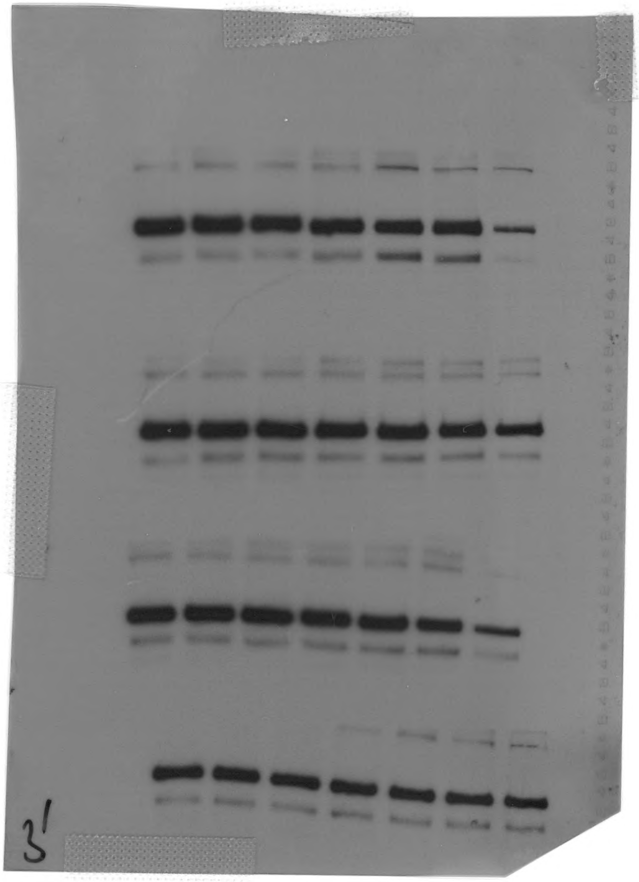

SCC-25

I. antibody ΔNp63 1,1 1:500

1) SODIUMBUTYRATE, 24h
20µg/30µl

2) VALPROIC ACID, 24h
20µg/30µl

3) SAHA, VORINOSTAT, 24h
20µg/30µl

4) NICOTINAMIDE, 24h
20µg/30µl

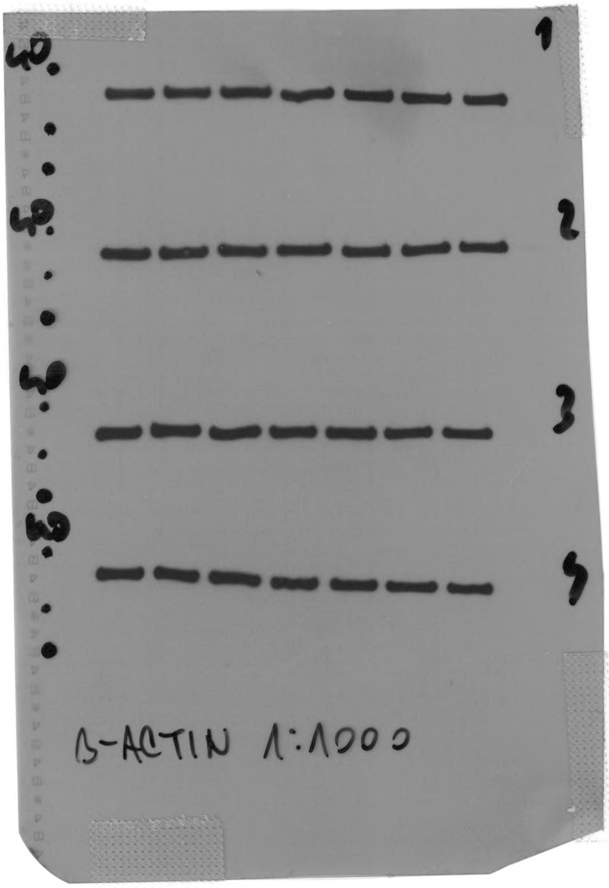

Supplement: Supplementary file 10 — Additional file 10. Full western blot images. [file 11658_2022_323_MOESM10_ESM.pdf]
